# POM‐Based Water Splitting Catalyst Under Acid Conditions Driven by Its Assembly on Carbon Nanotubes

**DOI:** 10.1002/adma.202512902

**Published:** 2025-11-26

**Authors:** Eugenia P. Quirós‐Díez, Melanie Guillén‐Soler, Carlos Herreros‐Lucas, Alejandro López‐Moreno, José Manuel Vila‐Fungueiriño, Antonio Luis Llamas‐Saiz, Karol Strutyński, Manuel Melle‐Franco, María del Carmen Giménez‐López

**Affiliations:** ^1^ Centro Singular de Investigación en Química Biolóxica e Materiais Moleculares (CiQUS) Universidade de Santiago de Compostela Santiago de Compostela 15782 Spain; ^2^ X‐Ray Unit Área Infraestruturas Investigación Universidade de Santiago de Compostela Santiago de Compostela 15782 Spain; ^3^ CICECO—Aveiro Institute of Materials Department of Chemistry University of Aveiro Aveiro 3810‐193 Portugal

**Keywords:** alcohol oxidation reaction, assembly, carbon nanotubes, electrocatalytic water‐splitting, polyoxometalates

## Abstract

Development of efficient and stable bifunctional electrocatalysts for water electrolysis under acidic conditions is essential for sustainable hydrogen production. A novel vanadium polyoxometalate (POM)‐based material, Na_4_(H_2_O)_12_[(CH_2_OH)_3_CNH_3_]_2_[V_10_O_28_]·4H_2_O (1) is presented, incorporating non‐innocent cations and whose electrocatalytic activity can be switched from the production of oxygen to hydrogen through its assembly on carbon nanotubes (CNT). A physical mixture (1/CNT) shows remarkable oxygen evolution reaction (OER) activity, with an overpotential of 0.34 V at 10 mA cm^−^^2^, outperforming commercial IrO_2_ (0.45 V) and approaching Ir/C (0.31 V), with 80% Faradaic efficiency. In contrast, directed assembly (1@CNT) unlocks TRIS ⁺= [(CH_2_OH)_3_CNH_3_]⁺ groups functionality, enabling high hydrogen evolution reaction (HER) efficiency, with an onset potential of −0.07 V, close to Pt/C, and 94% Faradaic efficiency. Mechanistic studies, strongly supported by in‐operando confocal microscopy and theoretical calculations, reveal that the modulation of crystal interactions and the local microenvironment is key to orchestrating the OER/HER tuning. OER is proposed to proceed via an alcohol oxidation reaction (AOR), while HER benefits from TRIS⁺ moieties acting as a “proton sponge”. This work provides a compelling approach for rational design of bifunctional molecular electrocatalysts based on earth‐abundant elements and controlled nanoassembly, with clear relevance for advancing green hydrogen production technologies.

## Graphical abstract

A newly‐engineered POM‐based electrocatalyst incorporating non‐innocent counter cations exhibits fast kinetics for either the OER or HER under strongly acidic conditions (1 m H_2_SO_4_), depending on whether it is assembled on carbon nanotubes (1@CNT) or physically mixed with them (1/CNT). In water‐splitting tests using a two‐electrode setup, these systems demonstrated significantly higher stability than state‐of‐the‐art benchmark electrocatalysts (Pt/C and Ir/C).

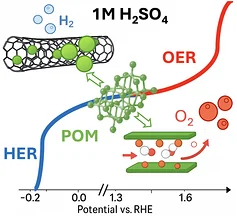

## 1 Introduction

Water splitting devices (2H_2_O → 2H_2_ + O_2_) are considered one of the most promising technologies for clean fuel (H_2_) production when coupled with renewable sources, aiming for a scalable and sustainable carbon‐free energy society.^[^ ^1^, ^2^ ^]^ Large‐scale production of H_2_ is still limited by the lack of cost‐efficient and abundant water splitting electrocatalysts for both the cathodic hydrogen evolution reaction (HER) and the anodic oxygen evolution reaction (OER), as relatively fast HER/OER kinetics are currently limited to the use of noble metals.^[^ ^3^, ^4^, ^5^, ^6^, ^7^, ^8^, ^9^ ^]^ Efforts have been made to overcome such obstacles by developing nanostructured electrocatalyst materials based on nitrides and carbides,^[^ ^10^, ^11^, ^12^ ^]^ phosphides,^[^ ^13^ ^]^ and chalcogenides.^[^ ^14^, ^15^, ^16^ ^]^ In particular, recent studies have demonstrated that tailoring the local reaction environment or introducing multi‐interfacial or nitrogen‐mediated effects can drastically enhance OER and HER activity under practical electrolysis conditions.^[^ ^17^, ^18^, ^19^ ^]^ However, the lack of molecular‐level control limits the development of novel metal‐free bifunctional electrocatalyst materials with technological interest under relevant pH conditions.^[^ ^20^, ^21^ ^]^

Among all the candidates, molecular metal oxide clusters, so‐called polyoxometalates (POMs), are one of the most interesting inorganic negatively‐charged clusters combining tunable sizes, enormous structural diversity,^[^ ^22^, ^23^, ^24^ ^]^ as well as fast reversible multielectron‐transfer reactions catalysis,^[^ ^25^ ^]^ storage,^[^ ^26^, ^27^ ^]^ electrocatalysis,^[^ ^28^, ^29^ ^]^ or magnetism.^[^ ^30^, ^31^ ^]^ Up to now, the strategy for designing active electrocatalysts has been the incorporation of heteroatoms^[^ ^32^, ^33^ ^]^ in the POM framework (mainly transition metals), which has proved to be very beneficial, especially for redox processes with a high number of electrons involved, as in the case of the proton‐coupled 4‐electron OER.^[^ ^34^, ^35^, ^36^ ^]^ On the other hand, the strong metal‐oxo double bonds stabilizing the POM have been proposed as proton transfer sites for water, enabling HER. Despite the excellent progress in the field, the ongoing debate about how to define the true catalyst in some cases (e.g., the formation of metal oxides as degradation products), together with the fact that POMs without water‐binding sites have also been found to act as active catalysts for water oxidation, still leaves some questions open.^[^ ^37^ ^]^

The role of counter cations in different electrocatalysts for fine‐tuning electrochemical processes has been largely under‐researched.^[^ ^38^ ^]^ It is known that alkali metal cations can facilitate the water hydrolysis step (i.e., the dissociation of the O─H bonds). The oxygen‐evolving center in photosystem II, which is characteristic of certain species in nature, contains alkali metals that are directly involved in the OER reaction due to their ability to adsorb and desorb water molecules.^[^ ^39^, ^40^, ^41^, ^42^, ^43^ ^]^ The electrochemical activity of pristine POM is intimately related to the nature of the counter cation (either organic or inorganic), which determines their assembly and crystal structure, together with their intrinsic low conductivity and their tendency to dissolve into the electrolyte.^[^ ^44^ ^]^ Thus, engineering new POM‐electrocatalyst materials with non‐innocent counter cations to tailor microenvironments, by tuning HER/OER water‐splitting kinetics through changes in local pH, mass transport, or active site blocking, is highly promising.

Through deliberate electrocatalyst design, alternative reaction pathways, such as the alcohol oxidation reaction (AOR), have recently emerged as promising strategies to overcome the sluggish kinetics of the anodic OER in water splitting.^[^ ^45^, ^46^, ^47^, ^48^ ^]^ By integrating AOR into water‐splitting systems, researchers aim to bypass the limitations of conventional noble metal‐based catalysts, paving the way for more sustainable and cost‐effective oxygen production, and ultimately enabling cleaner hydrogen generation. POMs, well known for converting alcohols into aldehydes, ketones, or carboxylic acids—often using hydrogen peroxide as a green oxidant that produces water as the main byproduct—represent a highly promising class of AOR mediators. Therefore, engineering POM‐based electrocatalysts that can not only exploit the synergy between non‐innocent counter cations but also enable alternative reaction pathways could open new opportunities in water splitting.

To the best of our knowledge, no examples have been reported of a single, discrete molecular metal cluster capable of catalyzing both HER and OER with fast kinetics, and critically, whose dominant electrocatalytic pathway can be controllably switched by external factors such as its assembly state.^[^ ^49^, ^50^ ^]^ In this regard, the interface with conductive supports is currently a technological demand to exploit POM water splitting properties.^[^ ^51^, ^52^, ^53^, ^54^, ^55^ ^]^ Porous carbon materials are the most widely studied electrocatalyst supports, as they combine large surface areas with high electrical conductivities.^[^ ^56^ ^]^ Highly graphitized carbon materials, such as carbon nanotubes, have been put forward due to their unique corrosion resistance.^[^ ^57^, ^58^, ^59^ ^]^ To achieve this coupling, different covalent functionalization approaches involving both components have been proposed.^[^ ^60^, ^61^ ^]^ While the covalent functionalization of carbon nanotubes limits their charge transport, the functionalization of POM is synthetically challenging, and no unified strategy currently exists.^[^ ^62^ ^]^ Thus, the use of electrostatic and/or van der Waals forces is very appealing.^[^ ^63^, ^64^, ^65^, ^66^ ^]^ It has been shown that molecular dispersion of POM on nanocarbons significantly improves their electrochemical properties.^[^ ^67^ ^]^ Despite the large number of examples, no reports based on non‐innocent organic counter cations tuning POM electrochemical behavior while directing the assembly on carbon nanotubes are known.

**Figure 1:**
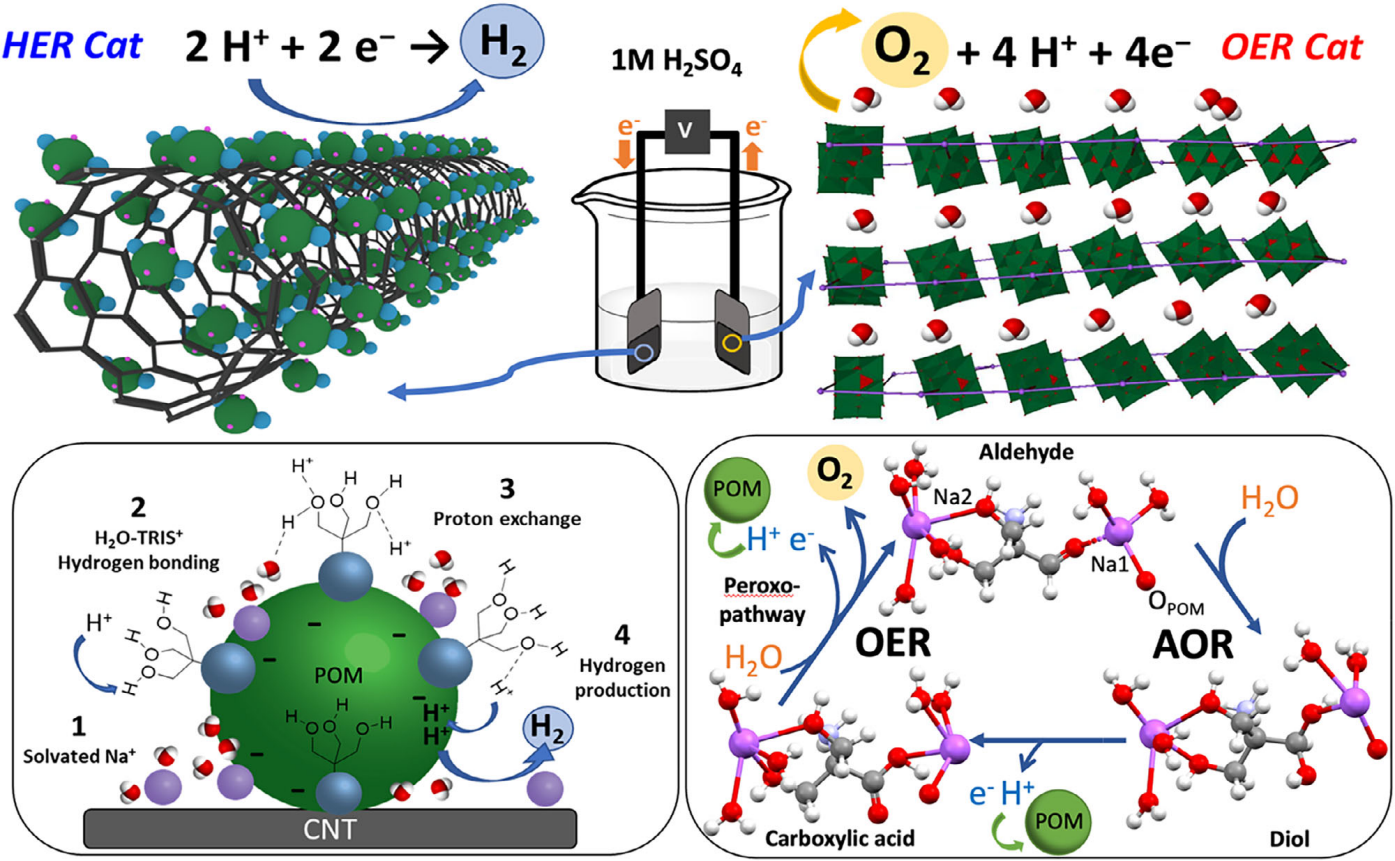
Bifunctional behavior toward water splitting under strong acid conditions (1 m H_2_SO_4_ previously degassed) of a vanadium POM‐based carbon hybrid catalyst incorporating non‐innocent cations depending on its assembly with carbon nanotubes (CNT). On the top left of the figure, a scheme for the assembly of anions ([V_10_O_28_]^6−^, green) and cations (Na^+^(purple) and TRIS^+^ (blue)) on CNT surfaces, and on the bottom, the proposed mechanism for the HER, highlighting the role of the triol groups as proton‐sponge groups via hydrogen bonding. On the top right of the figure, a scheme of **1** on a modified carbon electrode showing its layered structure enabling the diffusion of water molecules, and on the bottom, the proposed mechanism for the OER, highlighting the role of Na dimers (in purple) and TRIS molecules in the water hydrolysis mediated through the AOR.^[^ ^45^, ^46^, ^47^, ^48^ ^]^

Here, we report a new‐engineered vanadium POM‐based electrocatalyst material Na_4_(H_2_O)_12_[(CH_2_OH)_3_CNH_3_]_2_[V_10_O_28_]·4H_2_O (**1**) that incorporates non‐innocent counter cations and can perform either the OER (through an AOR) or the HER kinetically fast under strong acid conditions (1 m H_2_SO_4_ previously degassed), depending on whether it is assembled on (**1@CNT**) or physically mixed with carbon nanotubes (**1/CNT**) (**Figure** **1**). We propose that crystal interactions in **1** enhance water molecule diffusion on the electrode surface, as the O─H bonding dissociation is facilitated by Na^+^–Na^+^ dimer cations, while [V_10_O_28_]^6−^ anions act as local proton sinks assisting the electrocatalyst production of O_2_. Upon assembly on CNT, a proton‐sponge group from the organic cation TRIS^+^ = [(CH_2_OH)_3_CNH_3_]^+^ becomes unlocked, increasing the local concentration of protons around [V_10_O_28_]^6−^ and enabling the HER. **1/CNT** and **1@CNT** were tested simultaneously in a two‐electrode set‐up for water splitting, as anode and cathode, respectively, exhibiting increased stability compared to the state‐of‐the‐art benchmark electrocatalysts (Ir/C and Pt/C) under the same conditions.

## 2 Results and Discussion

### 2.1 Synthesis and Characterization of POMs

**Figure 2:**
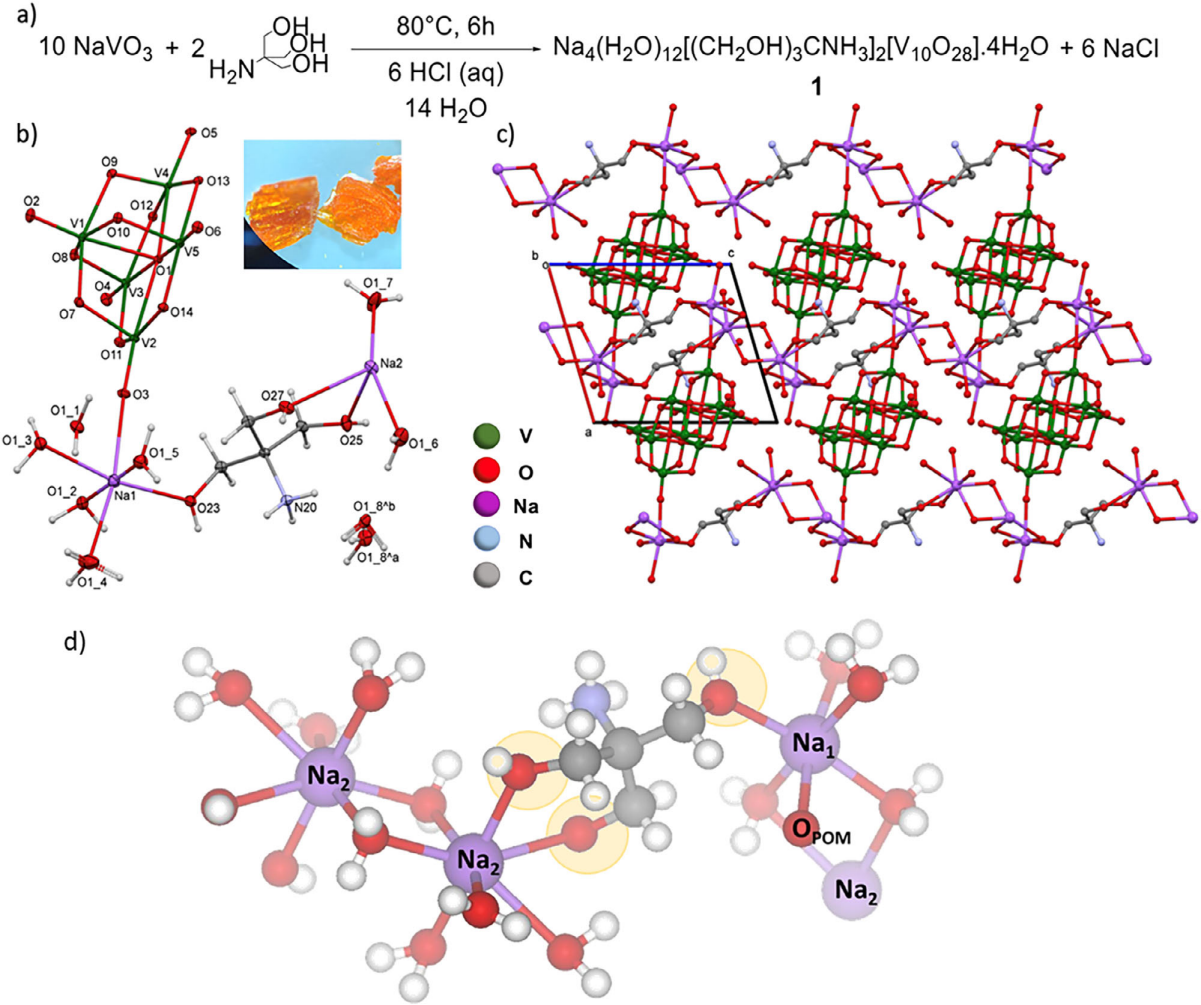
a) Synthetic scheme for the preparation of **1**; b) Oak Ridge Thermal Ellipsoid Plot (ORTEP) representation (50% probability) of the crystallographic asymmetric unit. c) Crystal packing of **1** showing [V_10_O_28_]^6−^ clusters linked to positively charge adjacent Na^+^–TRIS^+^–Na^+^ chains through terminal oxygen atoms. Hydrogen atoms have been omitted in (c) for clarity. d) Ball‐and‐stick representation of the connectivity between the [Na1(µ1‐OTRIS)(µ1‐O POM)(H_2_O)_4_] and [Na2(µ2‐OTRIS)(H_2_O)_5_] moieties in **1**.

To synthesize **1**, NaVO_3_ underwent a condensation reaction in water under acidic conditions upon the addition of aqueous HCl (pH ≈ 4) at 80 °C. Then, tris(hydroxymethyl)aminomethane ligand (TRIS) was added, and after 6 h, the reaction was cooled down. (**Figure** **2**a). Bar‐shaped orange single‐crystals were obtained by slow evaporation (10% yield), as shown by scanning electron microscopy (SEM) and confirming the presence of sodium in **1**, identified by energy dispersive X‐ray diffraction (Figure S1, Supporting Information). Using a variety of different techniques, involving spectroscopic, thermogravimetric, microscopic, and diffraction measurements, **1** was carefully studied. Single‐crystal X‐ray diffraction (SCXRD) (Table S1, Supporting Information) was employed to determine the crystal structure (triclinic, space group $P\bar{1}$) that is composed by positively charged chains parallel to the *c*‐axis formed by two different sodium moieties [Na1(µ_1_‐OTRIS)(µ_1_‐O POM)(H_2_O)_4_] and [Na2(µ_2_‐OTRIS)(H_2_O)_5_] connected by water bridges forming a Na1–Na2–Na2–Na1 chain structure that is joined through TRIS molecules. These chains in turn are linked by the clusters of [V_10_O_28_]^6−^ that bond via an oxygen bridge (V2‐O3‐Na1), giving rise to 2D neutral sheets growing along the (a˗b, c) plane. Interestingly, the sodium moieties are connected by the TRIS ligands through O─Na coordination bonds, blocking the hydroxyl groups. These sheets are brought together through H‐bonding interactions from either the ─NH_3_ group of the TRIS, the water molecules coordinated to the sodium cations, and the two additional non‐coordinated water molecules, giving rise to the complete layered crystal structure (Figure 2c; Figure S2, Supporting Information). Within the [V_10_O_28_]^6−^ unit, the vanadium cations are coordinated in an octahedral conformation with an oxidation state of +5 according to the V─O bond lengths found for the oxygen bridges (V─O─V) (2.3416(13)–1.8005(14) Å) and for the typical terminal oxo groups (V═O) (1.6210(14)–1.6081(14) Å) (Figure S3 and Table S2, Supporting Information). This is in agreement with the obtained Raman and Infrared (IR) spectra (Figure S4, Supporting Information), where the typical peaks of the [V_10_O_28_]^6−^ anion corresponding to V─O─V bridging bending modes (317 cm^−1^), antisymmetric stretching (833 cm^−1^), V═O stretching (952 cm^−1^) and symmetric stretching of (V_10_O_28_)^6−^ (997 cm^−1^) can be observed. (Spectrochimica Acta Part A 61 (2005) 829–834). FT‐IR spectrum of **1** shows the bands in the 3500–2000 cm^−1^ range corresponding to water molecules, and the 1050 (s) cm^−1^ band can be assigned to C─O bonds, which belong to the TRIS component. The peak at 973 (m) belongs to the V─Ot bond, while the peaks at 938 (s), 927 (s), 815 (m), 727 (m), and 592 (m) correspond to V─Ob bonds in the [V_10_O_28_]^6−^ polyoxometalate structure. From UV–vis measurements, the typical peaks (387, 241, and 220 nm) associated with the decavanadate anion can also be identified (Figure S5, Supporting Information). The thermogravimetric analysis (TGA) showed a weight loss of 16% between 260 and 530 °C (Figure S6, Supporting Information), which, together with N─H and C─O vibrations inferred from the IR measurements (Figure S4, Supporting Information), confirms the presence of TRIS in **1**. The Na─O distances observed (≈2.4 Å) for the [Na1(µ_1_‐O TRIS)(µ_1_‐OPOM)(H_2_O)_4_] moiety are close to the one expected for sodium ions with a coordination index of 6 (Figure S7 and Table S3, Supporting Information).^[^ ^68^ ^]^ In contrast to the Na1 center, the Na2 center shows a heptacoordination environment (Na2(µ_2_‐OTRIS)(H_2_O)_5_) where the ─OH groups from the TRIS bridging the sodium centers are blocked (Figure S8 and Table S4, Supporting Information). It is worth noting that two of the water molecules in both Na centers (O1_3 and O1_5 for Na1; O1_6 and O1_6_sym_ for Na2) are involved in bridges with another sodium ion forming a chain of sodium dimers (distance Na1–Na2 of 3.8322(13) Å and Na2–Na2_sym_ of 3.8386(17) Å) (Figure 2d; Figure S9 and Table S5, Supporting Information). Interestingly, one of the Na─O distances observed for one of these bridged water molecules is longer than the rest (2.7744(19) Å) (Table S4, Supporting Information). Another important feature of this structure is the block of ─OH groups in the TRIS molecule, which are fully coordinated to the Na1 and Na2 cations (Figure 2d). We hypothesized that the Na–Na dimers and the TRIS blockage may play a significant role in the POM´s catalytic behavior for the OER, contributing to the O─H bond activation. The purity of the crystals is also confirmed by bulk X‐ray diffraction (XRD) measurements (Figure S10, Supporting Information).

An analogous material to **1**, using potassium instead of sodium, of formula K_4_(H_2_O)_10_[(CH_2_OH)_3_CNH_3_]_2_[V_10_O_28_]•4H_2_O (**2**) was synthesized using a similar method to that of compound **1**. According to their SCXRD structure, this compound hinders the formation of TRIS⁺ bridges and therefore prevents the blocking of the OH groups in the crystal structure, as shown by SCXRD measurements (triclinic, space group $P\bar{1}$) (Figures S11–S16 and Tables S6–S10, Supporting Information). As in the case of **1**, this novel compound **2** is composed of two positively charged chains that grow parallel to the *c*‐axis. These chains consist of two distinct potassium moieties [K1(µ_1_‐O TRIS)(µ_2_‐O POM)(µ_1_‐O POM)_2_(H_2_O)_3_] and [K2(µ_1_‐O TRIS)(µ_1_‐O POM)_3_(H_2_O)_3_] connected by water bridges forming a K1–K2–K1–K2 chain structure that is further linked through TRIS molecules (Figure S12, Supporting Information). **2** was also fully characterized by spectroscopy and microscopy techniques, as well as by TGA (Figures S17–S19, Supporting Information). Additionally, bulk XRD analyses were performed to verify the purity of the sample (Figure S20, Supporting Information).

### 2.2 Electrochemical Characterization of 1

Before focusing on the water splitting cell design, the electrochemical properties of **1** were first investigated using a conventional three‐electrode setup, both in solution and immobilized on a glassy carbon electrode (GCE). To overcome the high intrinsic electrical resistance of the POM and to facilitate dispersion of the electrochemically active material on the electrode surface, **1** was initially physically mixed with carbon black (CB) and mineral oil (Nujol) to form a slurry. This slurry was subsequently deposited onto a square GCE, resulting in a carbon‐modified paste electrode (CMPE) denoted as **1/CB** (see Supporting Information for details). It is worth noting that this procedure maintains the crystal structure and all functional properties of **1** intact.

Cyclic voltammograms (CV) of the resulting CMPE in acidic media (Figure S21, Supporting Information) show that the observed current changes with applied potential can be attributed solely to **1**, as the electrode additives (mineral oil–that is electrochemically inert under the conditions of our experiments–and CB) and the TRIS ligand exhibit no redox processes under the same conditions (Figure S22, Supporting Information). It is worth noting that the experiments shown in Figure S22 (Supporting Information) display only non‐Faradaic processes, which are related to the double‐layer capacitance of the carbon support used. The current observed is distinct from the Faradaic processes associated with the V⁴⁺/V⁵⁺ peaks typically observed in V_10_ POM.^[^ ^69^ ^]^ Furthermore, an increase in current density and stability of the CMPE with increasing proton concentration in the electrolyte is observed (Figure S21, Supporting Information). This is consistent with the electrochemical studies of **1** performed in solution (Figure S23, Supporting Information), where a pH‐dependent multi‐electron redox activity is evident, following the reaction: (POM^m−^ + ne^−^ +nH^+^ ↔ H_n_POM ^(m + n)−^). Thus, the large peak separation observed in the CMPE at low acid concentrations (5 mM) is attributed solely to the high solution resistance under these conditions. It is noteworthy that while IR compensation would be the preferred method to study the system's behavior at low pH, our choice of a 1 m H_2_SO_4_ electrolyte for all main experiments effectively addresses the issue of high solution resistance, enabling a fair and accurate comparison of catalytic performance.

### 2.3 1 Physically Mixed with CNT as Modified Electrode (1/CNT) for the OER at Acid pH

**Figure 3:**
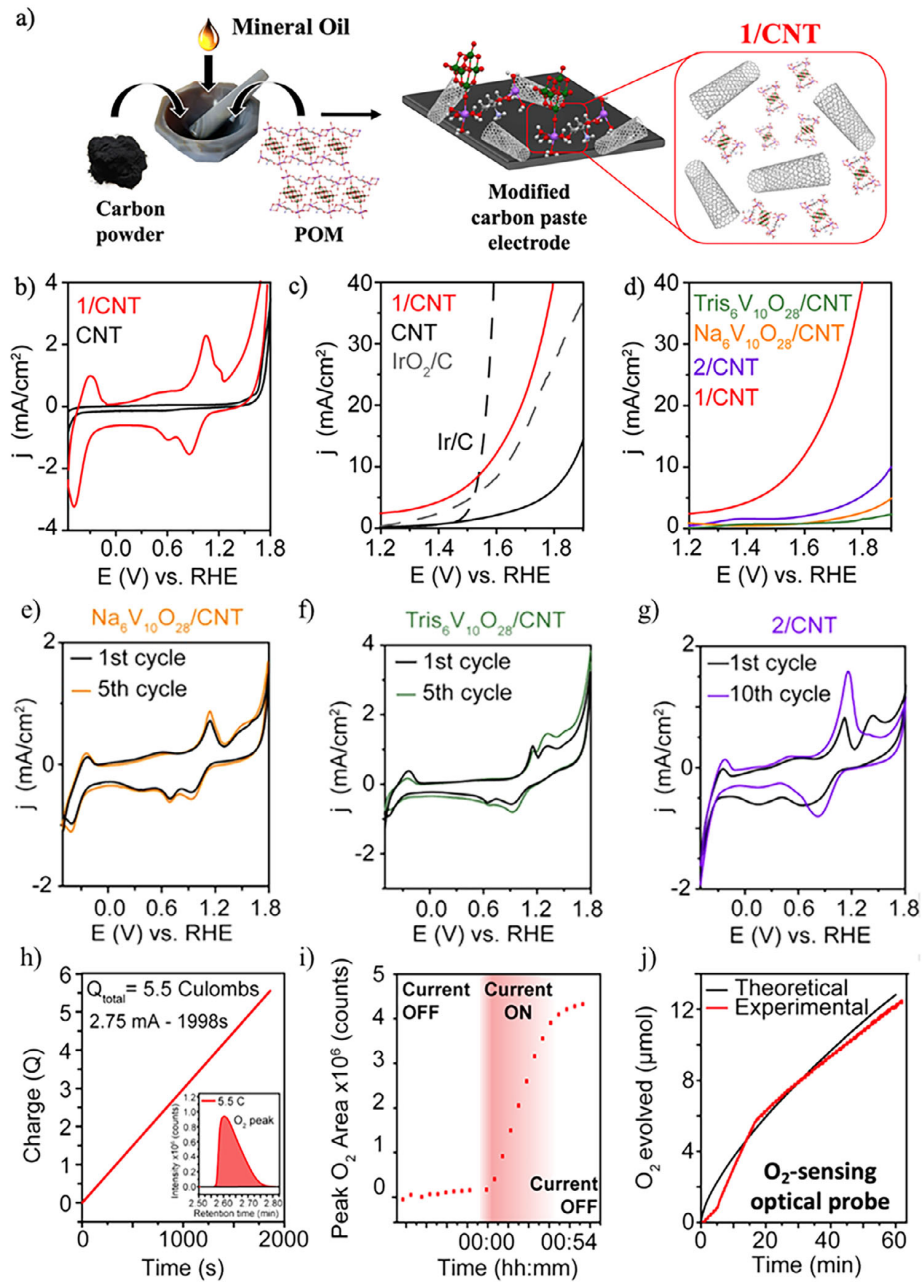
a) Schematic representation of the preparation of the CMPE for the electrochemical studies of **1** using CNT as the carbon source. b) Comparison of the CV measurements under nitrogen atmosphere of **1/CNT** (red) with CNT alone (black) (c) at 50 mV s^−1^. H_2_SO_4_ (1 m) was used as the electrolyte for all cases. c) Comparison of the OER catalytic behavior of 1/CNT (red) with CNT alone (black), using Ir/C (black dashed) and IrO_2_/C as standard electrocatalyst materials. d) OER measurements for the prepared vanadium‐based POMs containing only Na⁺ (orange) or only TRIS⁺ (green) as counter cations. Compound **2/CNT** was also tested for OER and compared with **1/CNT**. CV measurements performed under N_2_ 1 m H_2_SO_4_ for e) Na_6_POM/CNT, f) TRIS_6_POM/CNT, and g) **2/CNT**. h) Increase in the charge during a CA experiment matches with the increase in the O_2_ peak area measured by gas chromatography (GC) using a patented system (PCT/ES2023/070178). i) GC confirms the release of oxygen. j) Oxygen evolved (µmol) during the CA experiment detected with the oxygen sensor. For all the electrochemical measurements, the voltage potential is referred to RHE.

In contrast to CB, hollow carbon nanostructures such as highly graphitized CNT, which combine a well‐defined pore structure with excellent electrical conductivity and a large surface area, can be exploited to improve transport and electrical resistance–important drawbacks when working with carbon‐modified electrodes for technological applications. The **1/CNT** hybrid was prepared by a simple physical mixing approach following the Carbon Paste Modified Electrode (CPME) methodology. Briefly, 2.5 mg of crystalline compound **1** and 23 mg of carbon nanotubes (CNTs) were thoroughly ground in a mortar until a homogeneous mixture was obtained. Subsequently, 50 µL of mineral oil (Nujol) was added to produce a uniform slurry. The Nujol acted solely as an inert, non‐reactive binder required to ensure the mechanical stability of the paste electrode, as confirmed by control cyclic voltammetry (CV) measurements (Figure S22, Supporting Information). The resulting slurry was then deposited onto a glassy carbon electrode (GCE) to obtain the CPME. Thus, after careful optimization of the electrode preparation, CV measurements under acidic conditions (1 m H_2_SO_4_, pH< 0.3, Figure S24, Supporting Information) revealed current density values for **1/CNT** that were ten times higher than those for **1/CB** with the same amount of **1** (Figure S25, Supporting Information). Interestingly, while free‐standing CNT showed only a double‐layer capacitance in the potential region from −0.5 to 1.4 V, **1/CNT** exhibited clear multiple reversible redox processes assigned to the reversible V^4+^/V^5+^ redox couples occurring at the vanadium center of the POM (**Figure** **3**b) with the simultaneous insertion of H^+^.^[^ ^69^ ^]^ The smooth electron transfer between **1** and CNT, combined with a reduced contact resistance, explains the observed differences in current density and redox reversibility compared to **1/CB**.

As shown in Figure 3d and **1/CNT** exhibited an unexpected superior OER activity compared to **1/CB** (Figure S25, Supporting Information) and the rest of POM‐based electrocatalyst electrode materials reported recently, of which only very few are active at acid pH (Table S11, Supporting Information), which is quite remarkable. As expected for a highly active OER electrocatalyst electrode material, the obtained values for the onset and the overpotential at 10 mA cm^−2^ are relatively low (1.45 and 0.34 V, respectively) compared to the values obtained for the iridium standard electrocatalysts Ir/C (1.42 and 0.31 V) and IrO_2_/C (1.35 and 0.45 V) under the same pH and electrochemical conditions (Figure 3c).

To test separately the effect of Na⁺ and TRIS⁺ on the catalytic activity for the OER, we prepared crystals of a vanadium‐based POM containing only Na⁺ as counter cations (Na_6_V_10_O_28_) and POM crystals containing only TRIS⁺ as counter cations (TRIS_6_V_10_O_28_), following previously reported methods (Figures S26–S28, Supporting Information).^[^ ^70^, ^71^ ^]^ The corresponding hybrids were then prepared by mixing the active POMs with CNT, following the same method, to produce Na_6_V_10_O_28_/CNT and TRIS_6_V_10_O_28_/CNT, respectively. In both cases, CV measurements (Figure 3e–g) show the presence of the expected redox peaks of the V_10_ clusters,^[^ ^69^ ^]^ but neither showed activity for the OER. These results suggest that the presence of both counter cations is required to enhance the OER activity of **1**. This crucial finding directly demonstrates that the presence of the active [V_10_O_28_]^6−^ core alone is insufficient to drive the OER, and the catalytic activity is thus a result of the synergy between both the sodium and TRIS cations. This argument is further strengthened by measuring the **2/CNT** electrode for OER, which exhibited a significant decrease in catalytic activity when compared to 1/CNT (Figure 3d). It is hypothesized that the presence of a free ─OH group on the TRIS molecule in the K⁺–TRIS⁺–K⁺ chain of **2**, which is not present in **1**, affects the OER activity (Figure S12, Supporting Information). Moreover, longer K─O distances (∼ 2.9 Å) found in the [K1(µ_1_‐O TRIS)(µ_2_‐O POM)(µ_1_‐O POM)_2_(H_2_O)_3_] and [K2(µ_1_‐O TRIS)(µ_1_‐O POM)_3_(H_2_O)_3_] moieties (Tables S8 and S9, Supporting Information), compared to those observed in **1**, lead to longer K─K (∼4.6 Å) distances as well (Table S10, Supporting Information). These findings highlight the importance of the nature of the counter cations (Na⁺ or K⁺) in OER activity (Table S12, Supporting Information), as has already been demonstrated in other studies.^[^ ^38^ ^]^

To confirm and quantify the O_2_ gas produced, we used an optical oxygen sensor in a closed electrochemical cell to detect the released oxygen, along with GC in a homemade closed circuit. The combined detection of O_2_ of these two techniques allows us to further confirm the selective production of O_2_, as no other peaks are observed in the gas chromatogram. The amount of oxygen detected by GC closely matches the amount of charge produced during the chronoamperometry experiment. Specifically, a total of 5.5 coulombs were produced by applying a constant current while monitoring and quantifying the oxygen peak area, resulting in a faradaic efficiency of 80% for **1/CNT**. (Figure 3h–j).

**Figure 4:**
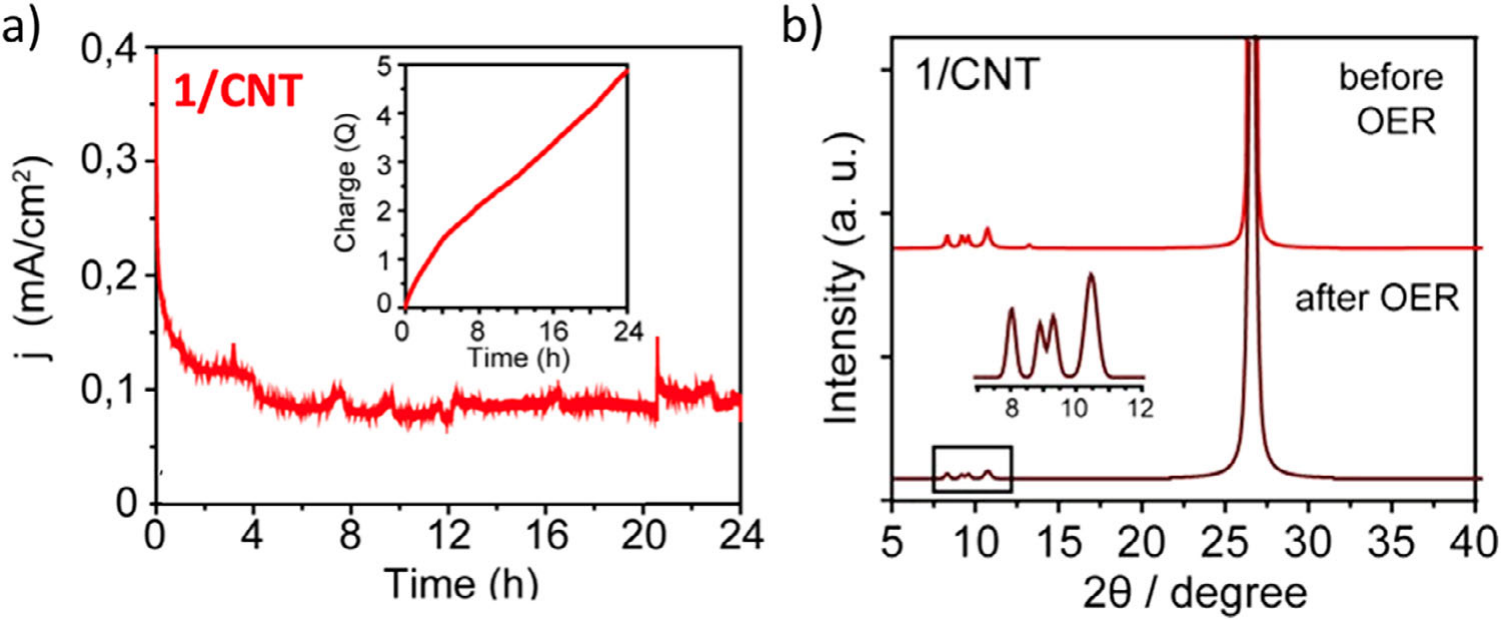
a) 24 h CA measurements of **1/CNT** for OER applied constant potential of 1.48 V. b) XRD measurements before and after the 24h‐CA OER of **1/CNT**.

To understand the stability of **1/CNT** through OER, we performed a 24h‐CA measurements in a half‐cell configuration (**Figure** **4**a). The current density remained stable throughout the 24 h period. Comparative measurements under identical conditions using commercial Ir/C are shown in Figure S29 (Supporting Information). Post‐catalytic SEM analysis for OER revealed no significant morphological changes on the electrode surfaces after the 24 h CA (Figure S30, Supporting Information). Furthermore, EDX spectra show the presence of vanadium and sodium after chronoamperometry experiments, suggesting that the dissolution of the POM is hindered by the formation of the paste. Additionally, the stability of **1/CNT** was corroborated by XRD (Figure 4b; Figure S31, Supporting Information), which reveals that the **1/CNT** not only shows the peak at 26.3° characteristic of CNTs, but also the diffraction pattern of **1** in the region from 7° to 15°, ruling out any chemical transformation of the POM to the oxide during the electrochemical tests. Thus, despite the harsh conditions (low pH and high potential), **1** remains on the surface of the electrode without any change in its structure (i.e., no vanadium oxide formation or leaching).

### 2.4 Assembly of 1 on CNT Surfaces (1@CNT) and its Exploitation as an Electrocatalyst Electrode Material for the HER at Acid pH

**Figure 5:**
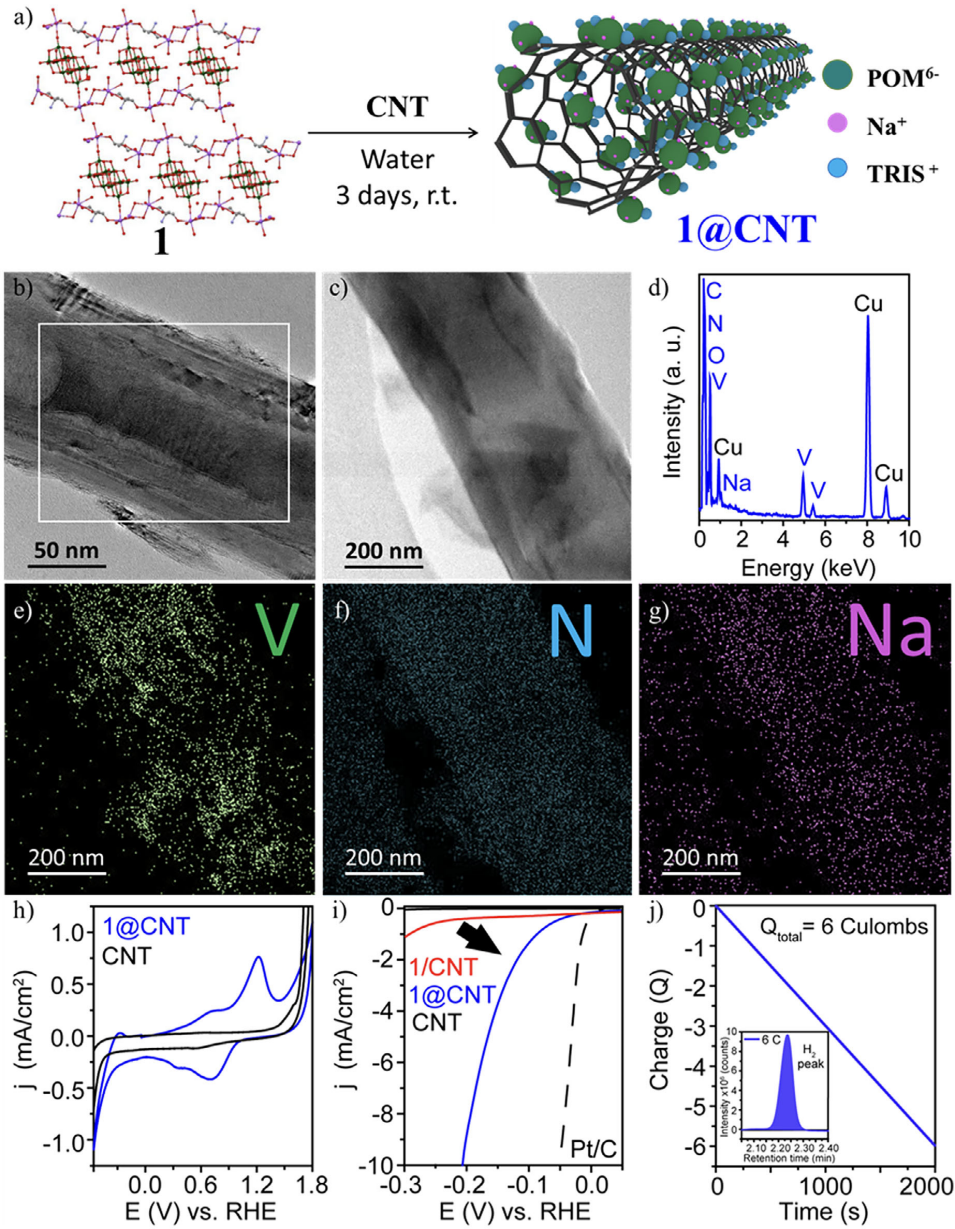
a) Scheme of the assembly process of [V_10_O_28_]^6−^, Na^+^ and TRIS⁺ of which **1** is composed to yield **1@CNT**. b) and c) HR‐TEM images at 200 kV for 1@CNT. d) EDX spectrum of a selected area in (b) and STEM‐EDS elemental mapping of (e) V; (f) N; and (g) Na for **1@CNT**. Note that copper in (d) is due to the copper grids used. h) Cyclic voltammogram under nitrogen atmosphere of **1@CNT** at 50 mV s^−1^ (blue) compared with CNT (black). i) Linear sweep voltammograms of **1/CNT** (red), **1@CNT** (blue), and CNT (black) between each other and the Pt/C standard catalyst for the HER. j) Increment of the charge during a CA experiment corresponds with the increment of the H_2_ peak in the gas chromatogram. Inset of the graph shows the H_2_ peak at 2.2 min in the gas chromatogram. For all the electrochemical measurements, the voltage potential is referred to the reversible hydrogen electrode (RHE).

CNT surfaces in a polar solvent such as water drive the assembly of the anions ([V_10_O_28_]^6−^) and cations (Na^+^ and TRIS^+^), of which **1** is composed, leading to a hybrid material **(1@CNT)** (**Figure** **5**a). The driving force for the assembly is mainly based on electrostatic interactions, considering the nature of CNT (Figure S32, Supporting Information), and no chemical transformation of **1** is observed during the assembly at mild conditions (Figure S33, Supporting Information). HR‐TEM of the hybrid **1@CNT** showed that an amorphous material is assembled on both the external and internal surfaces of CNT (Figure 5b; Figure S34, Supporting Information). Complementary, STEM‐EDS elemental mapping taken on bright‐field revealed that the amorphous material assembled on the CNT surfaces is composed of vanadium, nitrogen, and sodium, proving that CNTs have been internally and externally decorated with [V_10_O_28_]^6−^, Na^+^, and TRIS^+^ (Figure 5c–g; Figures S35 and S36, Supporting Information). It is proposed that this amorphous material is the electrochemically active one due to its large surface area in comparison with the large crystals. TGA of **1@CNT** in air (≈32% residual weight at 1000 °C corresponding to vanadium oxide^−^ presence) was carried out to ascertain the presence of TRIS as observed previously, in which a weight loss in the 260–530 °C temperature range was observed (Figure S37, Supporting Information). On the other hand, the progressive weight loss (<10%) observed below 200 °C can be assigned to the presence in the hybrid of water molecules that are strongly interacting with Na^+^ and [V_10_O_28_]^6−^. In contrast to the physically mixing method for the preparation of modified carbon electrodes, the directed assembly on CNT involves breaking crystal interactions. As shown by the FTIR data in Figure S38 (Supporting Information), the 1@CNT hybrid lacks the characteristic band at 1550 cm^−1^, which corresponds to the Na─O─C stretching mode. This band was a clear signature of the interaction between the sodium dimers and the carbon support in the 1/CNT hybrid. The absence of this key interaction in 1@CNT is expected to have a significant impact on the electrochemical behavior, especially given the crucial role of these non‐innocent ions.

As shown by CV measurements (Figure 5h), the [V_10_O_28_]^6−^ clusters assembled on CNT surfaces are still redox active at acid pH. However, as a consequence of the assembly, the electrochemical behavior of **1@CNT** has turned from exhibiting excellent OER activity to showing HER activity (Figure 5i; Figure S39, Supporting Information). Thus, **1@CNT** exhibits an excellent performance for the HER with an onset potential of −0.07 V approaching that of Pt on carbon (Pt/C) used as a standard electrocatalyst (≈0 V). The Tafel was also calculated from the slope of the overpotential vs log J plot and a 99 mV dec^−1^ Tafel value (Table S13 and Figure S40, Supporting Information). As shown in Figure 5j, a total of 5 coulombs was produced in a chronoamperometry experiment by applying a constant current. During this time, the hydrogen peak detected in the gas phase by GC was monitored and quantified. The peak at 2.2 min of retention time starts to appear when the reaction starts. After the experiment, the area of the peak was quantified and compared with the theoretical amount, giving an excellent faradaic efficiency of 94%.

To study the effect of Na⁺ and TRIS⁺ on the catalytic activity for the HER, the already prepared crystals in this work (Na_6_V_10_O_28_, TRIS_6_V_10_O_28_, and **2**) were also assembled into CNT surfaces in the same manner as **1@CNT**, yielding the materials Na_6_V_10_O_28_@CNT, TRIS_6_V_10_O_28_@CNT, and **2@CNT,** respectively. When we compare the polarization curves for the HER, we can clearly see an enhancement effect for the TRIS⁺ electrode with respect to the one containing only Na, which agrees with the fact that TRIS⁺ can act as a proton sponge, reducing the overpotential toward the HER (Figure S41, Supporting Information).

**Figure 6:**
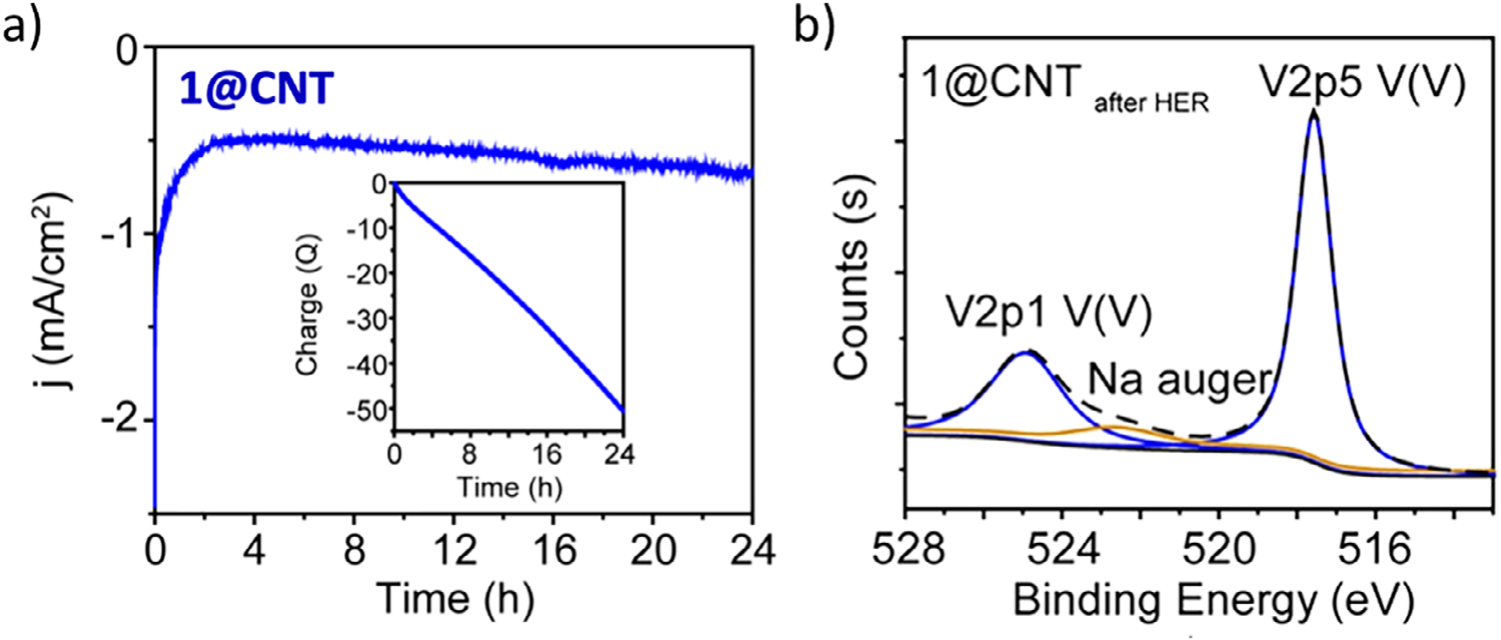
a) 24h‐CA measurements of **1@CNT** for HER applied constant potential of −0.2 V. b) XPS measurements after the 24h‐CA HER of **1@CNT**.

We aimed to perform a 24h‐CA experiment to test the stability of **1@CNT** for HER, benchmarking its performance against commercial Pt/C (Figure S29, Supporting Information). In **Figure** **6**a, it is shown that the current density remained stable over the entire period. Although large aggregates were observed in **1@CNT** by SEM, post‐catalytic analysis (Figure S42, Supporting Information) revealed no significant morphological changes on the electrode surfaces. Furthermore, EDX spectra showed that vanadium and sodium were still present on the electrode surface. X‐ray photoelectron spectroscopy (XPS) analysis after HER operation showed the characteristic peaks at 517 and 525 eV, assigned to V2p3(V) and V2p1(V), respectively, confirming that the oxidation state of the [V_10_O_28_]^6−^ cluster remains unchanged after H_2_ generation (Figure 6). It is worth noting that the 24h‐CA experiment was designed to provide a controlled, comparative assessment of the intrinsic robustness of the hybrid assemblies relative to commercial benchmark catalysts. Although this timescale is sufficient for fundamental‐level benchmarking, the catalysts should be integrated into proton‐exchange membrane water electrolyzer (PEMWE) cells for extended testing under practical operational conditions to fully establish their long‐term durability.

To further demonstrate the ability of **1** to act as a bifunctional electrocatalyst in acid media, the stability of **1/CNT** (as anode) and **1@CNT** (as cathode) was investigated in a 2‐electrode electrolyzer set‐up (Figure S43, Supporting Information). When benchmarked against the state‐of‐the‐art commercial Pt/C//Ir/C system under the same conditions, **1@CNT//1/CNT** exhibited superior initial robustness under coupled anodic and cathodic stress. After 120 s of operation, the current density decreased by 41%, compared to an 80% decrease observed for the Pt/C//Ir/C benchmark system. This short‐term stress test, together with the 24 h stability data, supports the promising durability and potential of the POM‐based bifunctional electrocatalyst for acid‐driven water splitting

### 2.5 Mechanistic Insights and Water‐Splitting Performance

To gain deeper insight into local microenvironmental changes during the HER and OER, we performed *in‐operando* confocal microscopy to visualize pH variations at the electrode surface. This technique enabled us to demonstrate the so‐called “microenvironment effect,” wherein the region surrounding the POMs becomes less acidic compared to the bulk electrolyte.^[^ ^72^ ^]^

Prior to these *in‐operando* experiments on **1@CNT** and **1/CNT** electrodes, we assessed the pH‐dependent fluorescence behavior of compound **1** using eosin Y as a pH‐sensitive probe. Under strongly acidic conditions (1 m H_2_SO_4_), no fluorescence emission was observed for either compound **1** or the **1** + eosin Y mixture. In contrast, both systems exhibited clear fluorescence at neutral pH (in water), confirming that emission increases with rising pH (pH >3) (Figure S44, Supporting Information). We then carried out *in‐operando* pH mapping of **1@CNT** and **1/CNT** using 10 µM eosin YS, which fluoresces at pH ≥3 and is non‐fluorescent at pH≈0.^[^ ^73^ ^]^

**Figure 7:**
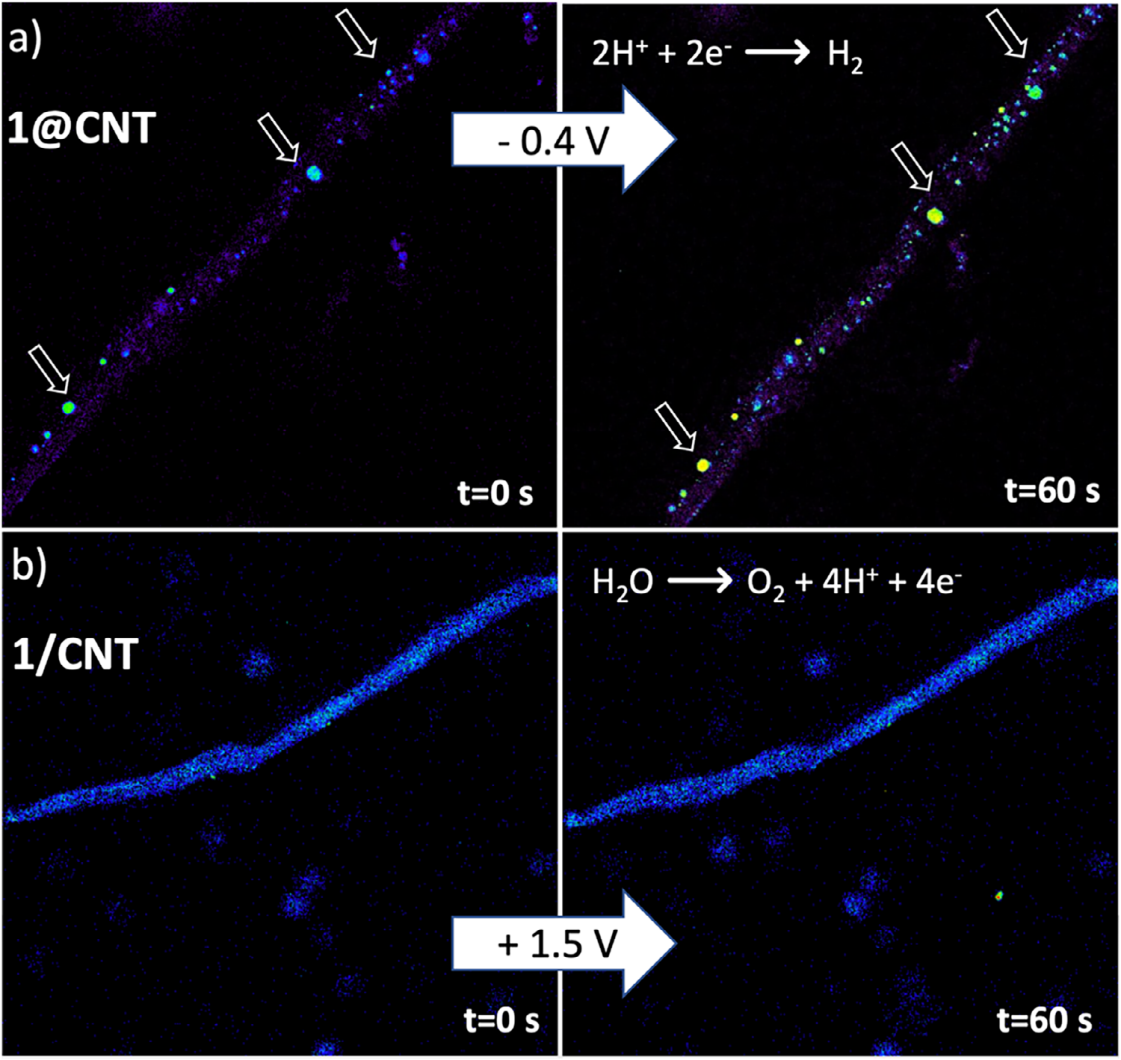
In‐operando visualization through confocal microscopy of a) **1@CNT** performing the HER, showing a change in the fluorescence, and b) **1/CNT** performing the OER with no change in the fluorescence, therefore no change in pH. Measurements were performed on a Leica SP2 confocal microscope (excitation: 488 nm; emission: 510–680 nm).

Upon applying a negative potential of −400 mV for 60 s, **1@CNT** showed increased fluorescence, consistent with local proton consumption expected during HER (**Figure** **7**a). In contrast, **1/CNT** showed no fluorescence change under positive bias, indicating that OER proceeds without a significant local pH increase—supporting our proposed mechanism involving an alcohol oxidation reaction (AOR), in which proton exchange at the interface is less pronounced than in direct water oxidation (Figure 7b). Upon applying a negative potential of −400 mV for 60 s, 1@CNT showed increased fluorescence, consistent with local proton consumption expected during HER (Figure 7a), thereby confirming the probe's sensitivity to local pH shifts. In contrast, 1/CNT showed no fluorescence change under positive bias (Figure 7b). This observation is critical because it confirms that the OER process, operating via AOR, achieves structurally enforced internal proton management. As hypothesized, protons generated during O─H activation are spontaneously sequestered by the adjacent [V_10_O_28_]^6−^ anion (the local proton sink), preventing their diffusion into the interfacial layer. This mechanism results in a markedly weaker rate of interfacial proton exchange compared to direct water oxidation, providing strong evidence for the AOR pathway. Note that these observations are qualitative, as we cannot provide an exact pH value for these experiments.

While a comprehensive investigation of the electrochemical mechanistic pathways lies beyond the scope of this work, we hypothesize that the key to tuning electrochemical responses and promoting bifunctional catalysts lies in two complementary strategies: i) controlling changes in crystal interactions through deliberate assembly, and ii) tailoring the microenvironment to modulate water‐splitting kinetics. This can be achieved by designing POM‐based carbon electrode materials incorporating non‐innocent cations with defined roles.

As illustrated in Figure 1, our hypothesis is that the cationic sodium dimers within compound **1** serve as the active sites for the OER. This role is facilitated by the unique crystal packing, which forms a water‐layered structure that reduces mass transport resistance. This structural arrangement enhances the diffusion of water molecules to the catalytic centers, thereby favoring O─H bond activation.^[^ ^37^ ^]^ Our SCXRD data provide the physical basis for this hypothesis by revealing Na─O bond distances of 2.36 and 2.50 Å, which are relatively long for a metal‐oxygen bond. This weakened interaction makes the bridging water molecule more susceptible to deprotonation by a nearby base, such as the [V_10_O_28_]^6−^ anion. The large [V_10_O_28_]^6−^ anion acts as a local proton sink, accepting the proton and facilitating the formation of a hydroxyl group on the sodium dimer, which enables efficient electron transfer to complete the catalytic cycle.

In contrast, when the crystal interactions are lost in the **1@CNT** electrode, the TRIS^+^ cations are released from the crystal lattice. We hypothesize that these unlocked TRIS^+^ cations, with their free triol groups, are electrostatically drawn to the POM surface and act as “proton sponges”. This increases the local concentration of protons around the [V_10_O_28_]^6−^ anion, facilitating its protonation to [H_n_V_10_O_28_]^(6‐n)−^),^[^ ^74^ ^]^ thereby reducing the overpotential toward the HER.^[^ ^75^, ^76^ ^]^ Recent studies highlighting the effect of the alkaline cations on the water oxidation and the weakening O─H bond, as well as the suppression of the HER acting as a competitive reaction, support this logical consideration.^[^ ^77^, ^78^ ^]^

**Figure 8:**
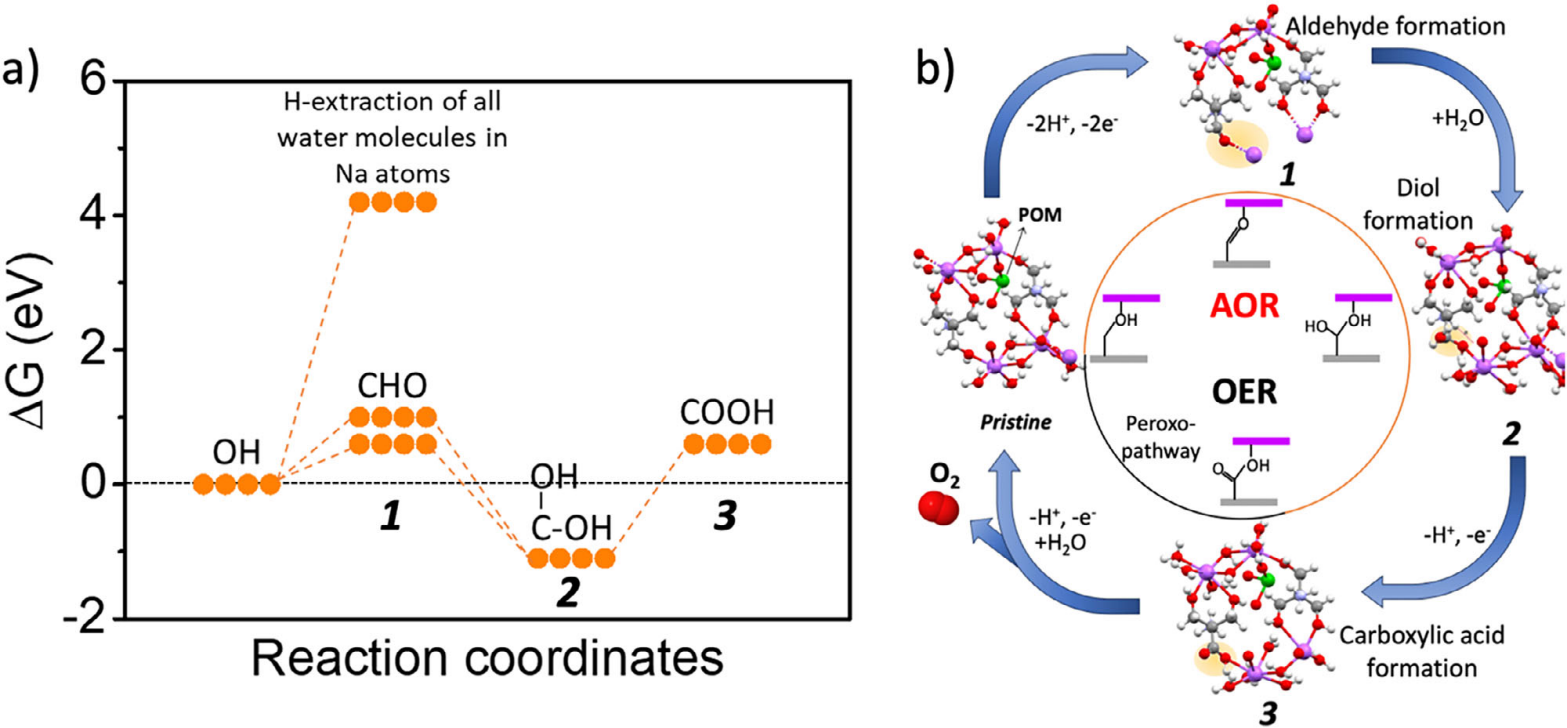
a) The Gibbs free energy profile of the reaction mechanism for AOR for compound **1**. b) A hypothesis mechanism for water splitting in **1**, featuring an OER mediated by an AOR. Sodium, vanadium, oxygen, carbon, and hydrogen atoms are shown in purple, green, red, grey, and light grey, respectively.

This mechanistic framework is well supported by combined experimental evidence, as shown above, and theoretical results proving that a more conventional OER mechanism is not energetically feasible. Our theoretical calculations using the xtb‐GFN1 tight methodology (see Experimental Section for details) directly support the O─H bond activation event triggered by the sodium dimers. The calculations demonstrate that the oxidation of the TRIS alcohol group is a key step, and appears to be triggered by the dissociation of O─H bonding of a water molecule bridging the sodium cations (Figure S45, Supporting Information for the proposed AOR mechanism mediating the OER).^[^ ^63^, ^64^, ^65^, ^66^ ^]^ The model provides a clear energetic profile for this AOR pathway, with key transformations from alcohol to aldehyde to diol to carboxylic acid, showing a spontaneous and exothermic proton transfer to the POM cluster. This alcohol‐aldehyde formation in TRIS molecules was found to be 0.4–0.6 eV per H / OH and 1 eV / OH / H for the full transformation. Then, the so‐formed aldehydes were found to react exothermically with a water molecule with an average energy of −1.1 eV, to form a diol. After that, the diol transformation to carboxylic acid, where the resulting carboxylic group was in the proximity of a vanadate, occurs spontaneously, and polyoxometalate adsorbs a carboxylic proton in an exothermic reaction, releasing up to 0.6 eV (**Figure** **8**). The proposed mechanism aligns with the big difference in the Tafel slope value observed for our system with respect to the Ir/C catalyst (Figure S46, Supporting Information). Altogether, the computer simulation results point to an active, non‐innocent behavior of the TRIS molecules, which, probably helped by the presence of the neighboring sodium dimers, through an AOR process involving aldehydes and carboxylic groups, mediate the OER facilitated by the proton‐sponge behavior of the nearby vanadate moieties (Figure 1).^[^ ^57^, ^58^, ^59^ ^]^

This AOR pathway, which involves the oxidation of the non‐innocent TRIS cation as a mediating step, also provides a direct quantitative explanation for the observed Faradaic efficiency. We measured a Faradaic efficiency of 80% for O_2_ production from 1/CNT. The 20% deviation is not attributed to degradation but to the intrinsic charge consumed by the mandatory intermediate oxidation steps (Alcohol → Aldehyde → Diol → Carboxylic Acid) necessary to sustain the AOR catalytic cycle. These steps utilize current that is diverted away from the direct 4e^−^ process producing O_2_, thereby confirming the AOR intermediate oxidation as a functional, quantitative signature of this unique catalytic mechanism.

## 3 Conclusion

We have demonstrated that controlling crystal interactions via assembly and designing new strategies for locking/unlocking electrochemical functional features of non‐innocent counter cations are key to developing POM‐based electrocatalyst materials capable of performing both OER and HER kinetically fast. This is of utmost importance for achieving bifunctional electrocatalysts under technologically relevant acid conditions, while increasing electrolyzers durability for water‐splitting. We have shown here, for the first time, the possibility of tuning water‐splitting kinetics in an orchestrated manner, when tailoring microenvironments around active centers ([V_10_O_28_]^6−^ for the HER and Na^+^–Na^+^ dimer for the OER) by either a change in the local concentration of protons and reactants, or both. Importantly, the superior durability and activity of our system over the state‐of‐the‐art Pt/C//Ir/C benchmark under acidic conditions further highlight its potential as a robust POM‐based platform for the design of next‐generation bifunctional electrocatalysts for acid‐driven water splitting. On the other hand, computational and experimental results consistently indicate that the OER in **1** proceeds AOR‐mediated pathway: i) As shown by a detailed and extensive state‐of‐the‐art computational study (Figure 8), the typical OER mechanisms are energetically inaccessible for the crystalline structure of **1**, whereas oxidation of the TRIS alcohol group—triggered by O─H bond dissociation in a water molecule bridging the Na⁺–Na⁺ dimer—is thermodynamically favored; ii) OER activity appears only in the intact Na–TRIS–Na framework; analogues lacking this motif show markedly reduced activity; iii) The large Tafel slope (360 mV·dec^−1^) contrasts with that of Ir/C (73 mV·dec^−1^), indicating a complex, multi‐electron oxidation step consistent with AOR. iv) The 80% efficiency reflects charge consumption by the oxidation of the TRIS intermediate, necessary to sustain catalysis; v) Confocal microscopy detected no pH change during OER, inconsistent with conventional pathways but compatible with internal proton buffering by the [V_10_O_28_]^6−^ cluster. The strategies proposed herein can be broadly applied to other POM systems and redox‐active molecular clusters, shedding light on current debates in the field and advancing the development of sustainable, cost‐effective OER materials based on abundant elements such as sodium.

## 4 Experimental Section

### Materials and Techniques

Sodium metavanadate, typically 96% and HCl (37%) were purchased from Alfa Aesar. Tris(Hydroxymethyl)aminomethane (TRIS) ultrapure (>99%) was purchased from Molekula. All these reagents and solvents were used as received. Wide carbon nanotubes (CNT) were purchased from Pyrograf Products Inc., USA. Carbon Black XC‐72 (CB) was purchased from FuelCellStore. Mineral oil (Nujol) and Iridium Oxide (IrO_2_) were purchased from Sigma–Aldrich. 20% Ir on Vulcan XC‐72 (Ir/C) was purchased from Premeter Co. Platinum, nominally 20% on carbon black (Pt/C), HISPEC 3000 was purchased from Alfa Aesar.

Infrared spectra were measured using a Bruker Alpha FTIR spectrometer with a platinum ATR module. UV–vis spectra (190–900 nm) were measured using a V‐750 JASCO spectrophotometer. TGA analysis was performed on a TA Instruments TGA‐SDTQ500 analyzer. Samples for TGA analyses were heated in air up to 1000 °C with a heating scan rate of 5 °C min^−1^. Scanning electron microscopy (SEM) and energy‐dispersive X‐ray spectroscopy (EDX) analysis were performed on a ZEISS EVO LS 15 with EDX module (Oxford Inca x‐act with 129 eV of resolution and WD 8.5). High‐resolution transmission electron microscopy (HR‐TEM) was performed on a JEOL JEM F200 microscope equipped with a cold field‐emission gun (Cold‐FEG) operated at 200 kV with an ultra‐high‐resolution pole piece. TEM images were acquired using a Gatan OneView camera. Energy Dispersive X‐ray Spectroscopy (EDS) was performed with a Centurio Large Angle Silicon Drift Detector (SDD) that collects X‐rays from a detection area of 100 mm^2^. Copper grids were used for HR‐TEM measurements. The Raman spectra were performed with a RENISHAW Raman microscope with laser Ion Ar (514 nm). Single‐crystal X‐ray diffraction of 1 was measured on a Bruker D8 Venture Photon 100 CMOS κ–geometry diffractometer system equipped with an Incoatec high brilliance IµS microsource (MoKα, λ = 0.71073 Å) and an Incoatec HeliosTM multilayer optics monochromator. Single‐crystal X‐ray diffraction of 2 was measured on a Bruker D8 Venture PHOTON‐III C14 κ–geometry diffractometer system equipped with an Incoatec high brilliance IµS 3.0 microfocus (CuKα, λ = 1.54178 Å) and a multilayer mirror monochromator. X‐ray Photoelectron Spectroscopy (XPS) analysis of the samples was performed using a Thermo Scientific K‐Alpha ESCA/XPS instrument equipped with aluminium Kα monochromatized radiation at 1486.6 eV X‐ray source. To guarantee a homogeneous surface conductivity, a charge compensation (electron flood gun) was used to minimize surface charging. Surface charge compensation was performed by using both a low‐energy flood gun (electrons in the range 0 to 14 eV) and a low‐energy Argon ions gun. The XPS measurements were carried out using monochromatic Al‐Ka radiation (hn = 1486.6 eV). Photoelectrons were collected from a take‐off angle of 90° relative to the sample surface. The measurement was done in a Constant Analyzer Energy mode (CAE) with a 100 eV pass energy for survey spectra and 30 eV pass energy for high‐resolution spectra. Surface elemental composition was determined using the standard Scofield photoemission cross sections. Binding energies (BE) were determined using the C 1s peak at 284.8 eV as a charge reference. The CasaXPS software was used to analyze the high‐resolution spectra. The background was fitted through a Shirley‐type function. Ball‐milling was carried out using a high‐energy Retsch MM400 ball mill instrument.

### Materials and Techniques—Synthesis of Na_4_ (H_2_O)_12_[(CH_2_OH)_3_CNH_3_]_2_[V_10_O_28_].4H_2_O (**1**)

In a 50 mL round‐bottom flask, 1.56 g of NaVO_3_ (12.36 mmol, 3 equiv.) was dissolved in 10 mL of water under stirring at 80 °C. After dissolving NaVO_3_, 4.12 mL (8.24 mmol, 2 equiv.) of an aqueous HCl solution (2 m) was added dropwise. The initial yellow solution became dark red upon the addition of the acid. Then, 0.5 g of TRIS [(CH_2_OH)_3_CNH_2_] (4.12 mmol, 1 equiv.) was added to the solution, and the reaction was stirred at 80 °C for 6 h. After that, the reaction was cooled down gradually to room temperature. Orange crystals of compound **1** were obtained after slow evaporation in air for three days. (10% of yield of Na_4_(H_2_O)_12_[(CH_2_OH)_3_CNH_3_]_2_[V_10_O_28_].4H_2_O **(1)**).

### Materials and Techniques—Synthesis of K_4_(H_2_O)_10_[(CH_2_OH)_3_CNH_3_]_2_[V_10_O_28_].4H_2_O (**2**)

In a 50 mL round‐bottom flask, 1.70 g of KVO_3_ (12.36 mmol, 3 equiv.) was dissolved in 10 mL of water under stirring at 80 °C. After dissolving NaVO_3_, 4.12 mL (8.24 mmol, 2 equiv.) of an aqueous HCl solution (2 m) was added dropwise. The initial yellow solution became dark red upon the addition of the acid. Then, 0.5 g of TRIS [(CH_2_OH)_3_CNH_2_] (4.12 mmol, 1 equiv.) was added to the solution, and the reaction was stirred at 80 °C for 6 h. After that, the reaction was cooled down gradually to room temperature. Orange crystals of compound **2** were obtained after slow evaporation in air for three days. (11% of yield of K_4_(H_2_O)_10_[(CH_2_OH)_3_CNH_3_]_2_[V_10_O_28_] **(2)**

### Materials and Techniques—Synthesis of 1@CNT, 2@CNT, Na_6_V_10_O_28_@CNT and TRIS_6_V_10_O_28_@CNT

A total of 100 mg of the material (**1**, **2**, Na_6_V_10_O_28_ or TRIS_6_V_10_O_28_) was dissolved in 1.5 mL of ionized water and added to a suspension of short CNT (5 mg in 1.5 mL of acetone) dropwise under sonication. The mixture was stirred at room temperature for 3 days, and then filtered through a PTFE membrane, washed repeatedly and copiously with water to collect a black solid (20 mg). Synthesis of Na_6_V_10_O_28_ and TRIS_6_V_10_O_28_ is available in the Supporting Information.

### Voltammetric Measurements

The electrochemical experiments were performed in a typical three‐electrode set‐up with an electrochemical Potentiostat AUTOLAB 302N using a glassy Carbon Electrode (GCE), a Hydrogen Electrode (RHE), and a carbon rod as working, reference, and counter electrode, respectively. Cyclic Voltammetry (CV) was performed in nitrogen‐saturated 1 m H_2_SO_4_ at scan rates of 50 mV s^−1^ between −0.5 and 1.8 V.

### Electrocatalytic Measurements

Electrocatalytic activities of the as‐prepared catalyst electrodes were examined by polarization curves using linear sweep voltammetry (LSV) at a scan rate of 5 mVs^−1^ conducted in 1 m H_2_SO_4_ solution. For OER, the electrolyte was saturated previously with oxygen, and the material was compared with the commercial Iridium on carbon (Ir/C) and Iridium Oxide (IrO_2_), whereas for HER, the electrolyte was saturated with hydrogen prior to the measurements, and the material was compared with the commercial Platinum on carbon (Pt/C) under the same conditions. Chronoamperometry experiments were carried out for 24 h in a 3‐set‐up cell using a carbon counter electrode and RHE, in a 1 m H_2_SO_4_ solution. For the electrodes **1/CNT** and Ir/C for OER it was applied a static overpotential of 250 mV (potential of 1.48 V) was applied for 24 h. For the electrodes **1@CNT** and Pt/C for HER, it was applied a static overpotential of 200 mV (potential of −0.2 V) was applied for 24 h. To evaluate the water splitting behavior, a typical water splitting cell (two electrode system) was constructed using **1/CNT** at the positive electrode, while 1@CNT was at the negative electrode. Chronoamperometry test was carried out in 1 m H_2_SO_4_ solution applying a potential of 3.1 V for 120 s. Background experiment of Pt/Ir was carried out under the same conditions by applying a voltage of 1.8 V.

### Gas Chromatography (GC) Measurements

Before each measurement, the electrochemical cell equipped with the electrodes (Ag/AgCl as reference and carbon as counter electrodes), 50 mL of electrolyte, and a stirrer was sealed and purged with nitrogen gas (N_2_) while stirring for at least 30 min. All experiments were performed at 298 K. Using a mass flow controller (Brooks Instrument SLA‐5850A, calibrated to N2/150NMLM/1), N_2_ was continuously pumped into the cell at a flow rate of 20 sccm. The electrochemical cell was connected to a gas chromatograph (Shimadzu Nexis GC‐2030) in a patented closed circuit for periodical sampling (PCT/ES2023/070178). A thermal conductivity detector (TCD) was used for detecting the targeted gas (O_2_ or H_2_). The carrier gas for the TCD was He.

In the GC experiment, after the cleaning period with nitrogen, several aliquots were taken into the GC for the background sampling. Then, during the electrochemical experiment where a constant current is applied for more than 30 min, several gas aliquots (100 µL) were automatically injected into the GC every 4 min. The current and dissolved oxygen concentrations were monitored during and after the electrochemical experiment with stirring.

The GC data collected in this work were translated to current efficiencies. It is assumed that the detected hydrogen and oxygen gases originated solely from electrochemical reactions, and that two electrons are required to form one H_2_ molecule and four electrons to form one O_2_ molecule.

### Confocal Microscopy Measurements

The microscope was a Leica SP2 using 488 nm to excite eosin YS, taking emission at 510–680 nm. The photomultiplier setting was kept constant so that the arbitrary fluorescence scale was constant. In this study, in‐operando visualization of the pH distribution was performed using 10 µM eosin YS (syn. tetrabromofluorescein, Fluka C.I. 45380, MW 692, stock 1 mM aqueous solution filtered on 0.45 µm) in the appropriate sulfuric acid. The solution and the electrodes were mounted in one chamber of the µ‐Slide 4 Well chambered coverslips. Due care was taken to protect the oil immersion objective from acid. Potentials are quoted against an Ag/AgCl reference electrode. The counter electrode was a platinum wire. The glassy carbon working electrode had a surface area of 0.35 cm^2^.

### Computer Simulations

A large number of different oxidation pathways were explored computationally, aided by the xtb‐GFN1 tight methodology.^[^ ^79^ ^]^ xtb‐GFN1 has been successfully applied in previous studies to study the OER of other 2D inorganic materials^[^ ^80^ ^]^ and has shown the spontaneous migration of acid protons in organic–inorganic hybrids.^[^ ^81^ ^]^ In addition, the computational efficiency of this methodology allowed for the explicit exploration of over 100 different reaction products here. 2D periodic models were used within the DFTB module of the Amsterdam Modeling Suite, version 2021.1, which allows for true 2D periodic models.^[^ ^82^ ^]^

### X‐Ray Crystallographic Data

Deposition Numbers CCDC‐1986505 and CCDC‐2062669 contain the supplementary crystallographic data for compounds **1** and **2**. These data are provided free of charge by the joint Cambridge Crystallographic Data Centre and Fachinformationszentrum Karlsruhe Access Structures service www.ccdc.cam.ac.uk/structures.

## Conflict of Interest

The authors declare no conflict of interest.

## Supporting information



Supporting Information

## Data Availability

The data that support the findings of this study are available in the supplementary material of this article.
