# Supplementary material for: POM‐Based Water Splitting Catalyst Under Acid Conditions Driven by Its Assembly on Carbon Nanotubes
Source: Adv Mater. 2025 Nov 26;38(51):e12902. doi: 10.1002/adma.202512902 (PMC13568918; doi:10.1002/adma.202512902)
Supplement: Supplementary file 1 [file ADMA-38-e12902-s001.pdf]

Supporting Information

POM-based Water Splitting Catalyst Under Acid Conditions Driven by its Assembly on Carbon Nanotubes

Eugenia P. Quirós-Díez, [a]§ *Melanie Guillén-Soler,* [a]§ *Carlos Herreros-Lucas,* [a] *Alejandro López-Moreno,* [a] *José Manuel Vila-Fungueiriño,* [a] *Antonio Luis Llamas-Saiz,* [b] *Karol Strutyński,* [c] *Manuel Melle-Franco,* [c] *María del Carmen Giménez-López**[a]

[a] E. P. Quirós-Díez, M. Guillén-Soler, C. Herreros-Lucas, A. López-Moreno, J.M. Vila-Fungueiriño, M. C. Giménez-López. Centro Singular de Investigación en Química Biolóxica e Materiais Moleculares (CiQUS), Universidade de Santiago de Compostela, 15782 Santiago de Compostela, Spain.
[b] A. L. Llamas-Saiz. X-Ray Unit, Área Infraestruturas Investigación, Universidade de Santiago de Compostela, 15782 Santiago de Compostela, Spain.
[c] K. Strutyński, Dr. M. Melle-Franco. CICECO—Aveiro Institute of Materials, Department of Chemistry, University of Aveiro, 3810-193 Aveiro, Portugal.
§These authors contributed equally to the work.

E-mail corresponding author: maria.gimenez.lopez@usc.es

Synthesis

Synthesis of $[(CH_2OH)_3CNH_3]_6[V_{10}O_{28}]$ ($TRIS_6V_{10}O_{28}$):

We followed a previous described method in literature.[1]
A 40 mL aqueous suspension of V_2O_5 (0.460 g, 2.50 mmol) received 10 mL of an aqueous solution of TRIS $[(CH_2OH)_3CNH_2]$ (0.360 g, 3.00 mmol). After stirring at room temperature for 2 days, the initial suspension turned into an orange solution that was then stored at 4°C with an overlayer of isopropanol. Orange crystals formed after 2 days were dried in air (7% of yield of $[(CH_2OH)_3CNH_3]_6[V_{10}O_{28}]$). SCXRD measurements confirm the same unit cell than the reported one (**reported:** a: 10.3934(6) Å, b: 16.3523(11) Å

and c:15.9974(10) Å; **experimental:** a: 10.393(4)Å, b: 16.352(3)Å and c: 15.997(8)) Å).

Synthesis of $Na_6[V_{10}O_{28}].18H_2O$ ($Na_6V_{10}O_{28}$):

We followed a previously described method in literature.[2,3]

A mixture of 50 g (0.182 mol) of commercial sodium (ortho)vanadate hydrate ($Na_3VO_4 \cdot nH_2O$) and 100 mL of water is stirred with a magnetic bar for 15 minutes, or until nearly all the solid is dissolved, at about 30'. Glacial acetic acid (50 mL total, 1.142 mol) is added with vigorous stirring over a period of 15-20 minutes, keeping the temperature in the 30-35°C region. The solution becomes orange, and toward the end an orange crystalline product is deposited. The solution is stirred for 1 hour at 25°C and the crystalline product is collected on a filter, washed four times with 50 mL portions of acetone, and air dried. The yield of crude product is approximately 25 g. Purification is accomplished by crystallization from a solution prepared from the crude product and 100 mL of water at a maximum temperature of 50°C (the time at this temperature should be kept minimal). Filtration of the nearly saturated solution through filter paper removes impurities of low solubility. At 0°C the solid product crystallizes slowly; 12 hours are required for complete deposition. The orange solid is collected on a filter and dried under atmospheric conditions (not vacuum). Yield: 4%.

Shortening of CNT:

CNT were shortened by mechanical ball milling. In a typical experiment, 50 mg of CNT were placed into a stainless-steel container (5 mL) with a stainless-steel ball (10 mm diameter) and milled in air during 90 min at 10 Hz.

Single-crystal X-Ray Diffraction (SCXRD) measurements

COMPOUND 1:

A dark yellow prism-like specimen of $Na_4(H_2O)_{12}[(CH_2OH)_3CNH_3]_2[V_{10}O_{28}].4H_2O$ with approximate dimensions of 0.073 mm x 0.131 mm x 0.209 mm, was used for the X-ray crystallographic analysis. The X-ray intensity data were measured on a Bruker D8 Venture Photon 100 CMOS κ--geometry diffractometer system equipped with a high brillance IμS microsource (MoKα, λ = 0.71073 Å) and a Incoatec HeliosTM multilayer optics monochromator. A total of 3538 frames were collected. The total exposure time was 4.71 hours. All crystals examined were twinned. The selected one for

data collection was indexed using CELL_NOW 2008/4 software (Sheldrick, G. M. (2008). *CELL_NOW*. University of Göttingen, Germany) giving rise to two twin domains related by the following reciprocal space matrix (0.976 -0.008 0.062 / -0.030 0.987 0.070 / -0.055 -0.058 1.030), i.e., a rotation of 4.6 degrees about reciprocal axis (1.000 -0.799 0.286). The frames were integrated with the Bruker SAINT software package (Bruker (2012). *APEX3, SAINT*. Bruker AXS Inc., Madison, Wisconsin, USA) using a narrow-frame algorithm and taken into consideration both twin domains simultaneously. The integration of the data using a triclinic unit cell yielded a total of 168552 reflections to a maximum θ angle of 33.83° (0.64 Å resolution), of which 9235 were independent (average redundancy 18.24, completeness = 97.9%, R_{int} = 5.64%, R_{sig} = 3.17%) and 7676 (83.12%) were greater than 2σ(F^2). The final cell constants of a = 10.7242(7) Å, b = 10.9128(6) Å, c = 11.9130(7) Å, α = 73.509(2)°, β = 68.228(2)°, γ = 66.779(2)°, volume = 1174.28(12) $Å^3$, are based upon the refinement of the XYZ-centroids of 9604 reflections above 20 σ(I) with 4.809° < 2θ < 67.48°. Data were corrected for twin and absorption effects using the Multi-Scan method implemented in TWINABS 2012/1 (Sheldrick, G. M. (1996). *TWINABS*. University of Göttingen, Germany). The ratio of minimum to maximum apparent transmission was 0.772. The calculated minimum and maximum transmission coefficients (based on crystal size) are 0.6720 and 0.8640. The structure was solved with SHELXT 2014/5 (Sheldrick, G. M. (2015). "SHELXT--Integrated space-group and crystal-structure determination", Acta Cryst. A71, 3-8.) and refined with SHELXL-2017/1 (Sheldrick, G. M. (2015) "Crystal structure refinement with SHELXL", Acta Cryst., C71, 3-8) software, using the space group P-1, with Z = 2 for the formula unit, $C_4H_{28}NNa_2O_{25}V_5$. The final anisotropic full-matrix least-squares refinement on F^2 with 406 variables converged at R1 = 3.74%, for the observed data and wR2 = 8.10% for all data. The goodness-of-fit was 1.131. The largest peak in the final difference electron density synthesis was 0.792 $e^-/Å^3$ and the largest hole was -0.777 $e^-/Å^3$ with an RMS deviation of 0.139 $e^-/Å^3$. Based on the final model, the calculated density was 2.237 g/cm^3 and F (000), 792 e^-. The final refined twin domains proportions were 87.7(1)% and 12.3(1)%.

COMPOUND 2:

A clear dark orange block-like specimen of $C_4H_{22}K_2NO_{22}V_5$, approximate dimensions 0.119 mm x 0.137 mm x 0.195 mm, was used for the X-ray crystallographic analysis. The X-ray intensity data were measured on a Bruker D8 VENTURE PHOTON-III C14 κ-geometry diffractometer system equipped with a Incoatec IμS 3.0 microfocus sealed tube (Cu Kα, λ = 1.54178 Å) and a multilayer mirror monochromator. A total of 5739 frames were collected. The total exposure time was 4.73 hours. The frames were integrated with the Bruker SAINT software package using a narrow-frame algorithm. The integration of the data using a triclinic unit cell yielded a total of 45978 reflections to a maximum θ angle of 79.08° (0.79 Å resolution), of which 4610 were independent (average redundancy 9.974, completeness = 99.2%, R_{int} = 4.80%, R_{sig} = 2.25%) and 4457 (96.68%) were greater than $2\sigma(F^2)$. The final cell constants of a = 9.60790(10) Å, b = 11.0420(2) Å, c = 11.3028(2) Å, α = 109.2809(6)°, β = 104.8997(6)°, γ = 95.8404(6)°, volume = 1070.66(3) $Å^3$, are based upon the refinement of the XYZ-centroids of 9654 reflections above 20 σ(I) with 8.716° < 2θ < 159.3°. Data were corrected for absorption effects using the Multi-Scan method (SADABS). The ratio of minimum to maximum apparent transmission was 0.334. The calculated minimum and maximum transmission coefficients (based on crystal size) are 0.0990 and 0.1780. The structure was solved and refined using the Bruker SHELXTL Software Package, using the space group P -1, with Z = 2 for the formula unit, $C_4H_{22}K_2NO_{22}V_5$. The final anisotropic full-matrix least-squares refinement on F^2 with 364 variables converged at R1 = 2.67%, for the observed data and wR2 = 7.24% for all data. The goodness-of-fit was 1.041. The largest peak in the final difference electron density synthesis was 0.563 $e^-/Å^3$ and the largest hole was -0.869 $e^-/Å^3$ with an RMS deviation of 0.120 $e^-/Å^3$. On the basis of the final model, the calculated density was 2.386 g/cm^3 and F(000), 764 e^-.

Electrochemical measurements

General procedure for the electrode preparation for the Oxygen Evolution Reaction (OER) and 24 hours chronoamperometry (CA).

Carbon paste modified electrode (CPME) was prepared as followed. In a typical experiment, 23 mg of the carbon source powder (*i. e.* CB or short CNT) and 2.5 mg of the material (**1, 2, $TRIS_6V_{10}O_{28}$ or $TRIS_6V_{10}O_{28}$)** were nicely mixed in a mortar to obtain a homogeneous mixture and 50 μL of Nujol (mineral oil) was added to form a slurry. Then,

it was spread on a Glassy Carbon Electrode (GCE) with a surface area of 1 cm^2. The electrode was left to dry prior to electrochemical measurements. The same procedure was followed for the 24h-CA experiment. Note that Nujol additive is inert according to electrochemical experiments (Figure S13).

For TON calculations, the electrodes were weighted after drying and it is considered only an amount of 5.2 ± 0.5 mg of the paste material, being a 10% loading of the catalyst. For Ir/C electrode, the same procedure was followed, and the metal loading was 1 mg.

General procedure for the electrode preparation for the Hydrogen Evolution Reaction (HER) and 24 hours chronoamperometry.

1 mg of the material (**1@CNT, 2@CNT, $TRIS_6V_{10}O_{28}$@CNT or $TRIS_6V_{10}O_{28}$@CNT**) was nicely dispersed in 0.5 mL of isopropanol. 50 µL of the suspension were drop casted to form a film in the glassy carbon electrode, being the final loading 0.1 mg of the material on the GCE (surface area = 0.196 cm^2). Then, 30 µL of Nafion solution (1%) was added. The electrode was left to dry prior to electrochemical measurements.

For 24h-CA experiment, the electrode was prepared as follows: 5 mg of the material (**1@CNT**) was nicely dispersed in a mixed solution of 2 mL of isopropanol and 40 µL of Nafion (1%) and sonicated for 15 minutes. Then, 800 µL of the suspension were drop casted to form a film in the glassy carbon electrode (surface area = 1 cm^2), being the final loading 2 mg of **1@CNT** on the GCE. The electrode was left to dry prior to electrochemical measurements. For Pt/C electrode, the metal loading was 1 mg.

Computational details.

The modelled system was built from a 2D monolayer cut out from the crystalline structure onto which 20 extra water molecules were added to approximately mimic the effect of the solvent, **Figure S47**. First, we explored the possible reaction pathways, by H subtraction, on each water molecule in the direct vicinity of a Na ion. This pathway was found to be unlikely energetically with unbiased potentials between 2 and 4.2 eV. Interestingly, during this process, we observed that, in a few cases, the reduction step induced the dehydrogenation of an alcohol group, with the subsequent spontaneous loss of an α-H to a neighbouring vanadate, forming an aldehyde and involving up to three hydrogen transfers, **Figure S47**, b). This prompted us to evaluate explicitly the possibility of alcohol-aldehyde formation in TRIS molecules which was found to be 0.4-0.6 eV per

H / OH and 1 eV / OH / H for the full transformation. Then, the so-formed aldehydes were found to react exothermically with a water molecule with an average energy of -1.1 eV, to form a diol. All this indicates that, under oxidizing conditions, the alcohol groups from the TRIS molecules will transform into diols. Note, that for both, a direct interaction with the Na dimers and the oxygen atoms of the oxidised moieties was also observed, see Figure S47, c) and d)). The diols transformation to carboxylic groups through subtraction of an α-H revealed potentials of 1-1.31 eV in four positions, while in the other two cases, where the resulting carboxylic group was in the proximity of a vanadate, the latter, through a barrierless process, spontaneously and adsorbed a carboxylic proton in an exothermic reaction releasing up to 0.6 eV, Figure S47, e) and f).

Characterization

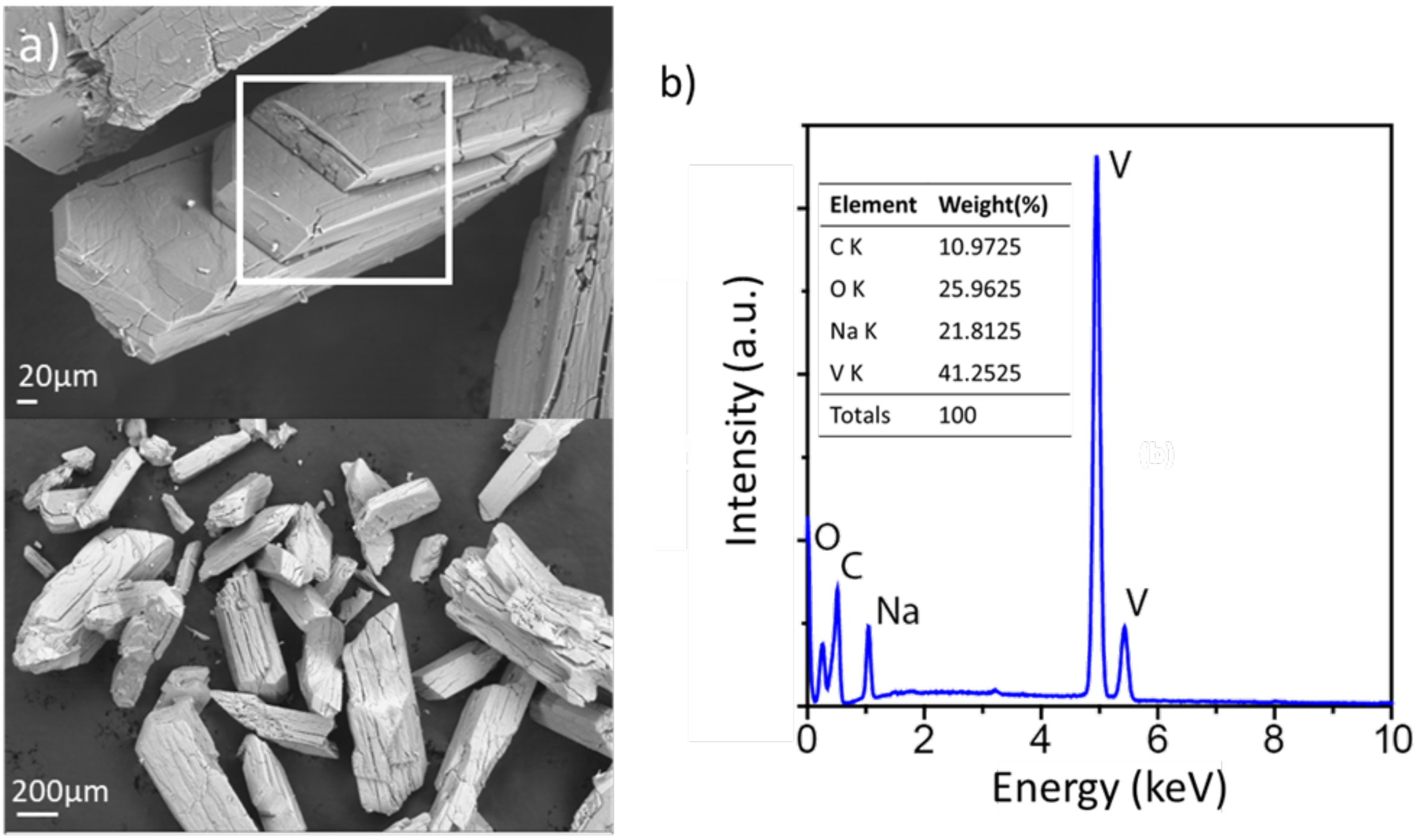

Figure S1. a) SEM images acquired at 20 kV and b) EDX spectrum of a selected area in (a) showing the crystals formed during the reaction of **1**.

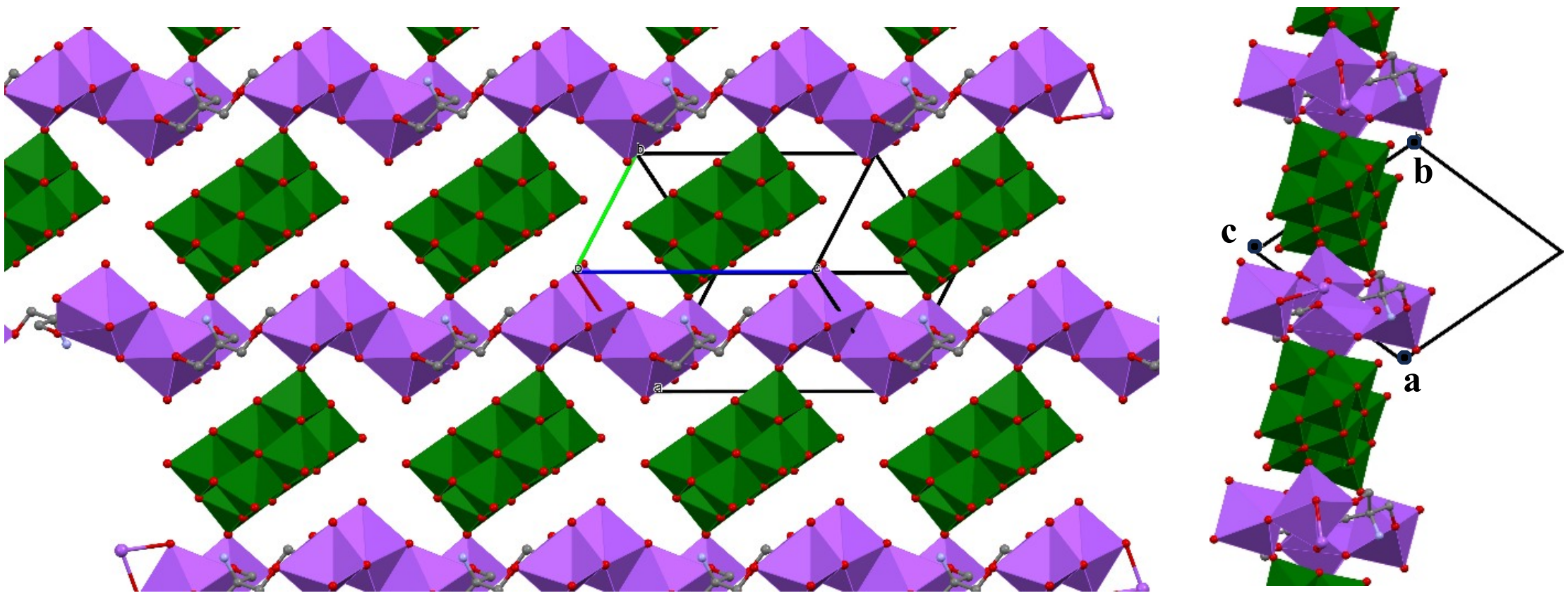

Figure S2. Two perpendicular views of the polyhedral representations of a single layer of **1**. All hydrogen atoms and the non-coordinated water molecules have been omitted in the figure for clarity.

Table S1. The structure of **1** was determined by single-crystal X-ray diffraction (SCXRD). The crystallographic data and structure refinement are shown in this table.

	**1**
**Chemical Formula**	$C_4H_{28}NNa_2O_{25}V_5$
**Size (mm)**	0.209 x 0.131 x 0.073
**Formula weight (amu)**	790,95
**Crystal system**	Triclinic
**Space group**	$P\bar{1}$
**a [Å]**	10.7242(7)
**b [Å]**	10.9128(6)
**c [Å]**	11.9130(7)
**α [°]**	73.509(2)
**β [°]**	68.228(2)
**γ [°]**	66.779(2)
**V [Å^3]**	1174.28(12)
**Z**	2
**Dx [mg/m^3]**	2.237
**Temperature [K]**	100
**Tmax/Tmin**	0.67/0.86
**Absortion coeff. [mm^{-1}]**	2.07
**F (000)**	792
**Θ range [°]**	2.4 to 33.8
**Reflections collected**	168552
**Independent reflections**	9235 [R(int) = 0.0564]
**GOF on F2**	1.131
**Final R indices [I > 2σ(I)]**	R_1=0.0374; wR_2= 0.0737
***R* indices (all data)**	R_1=0.0561; wR_2=0.0810

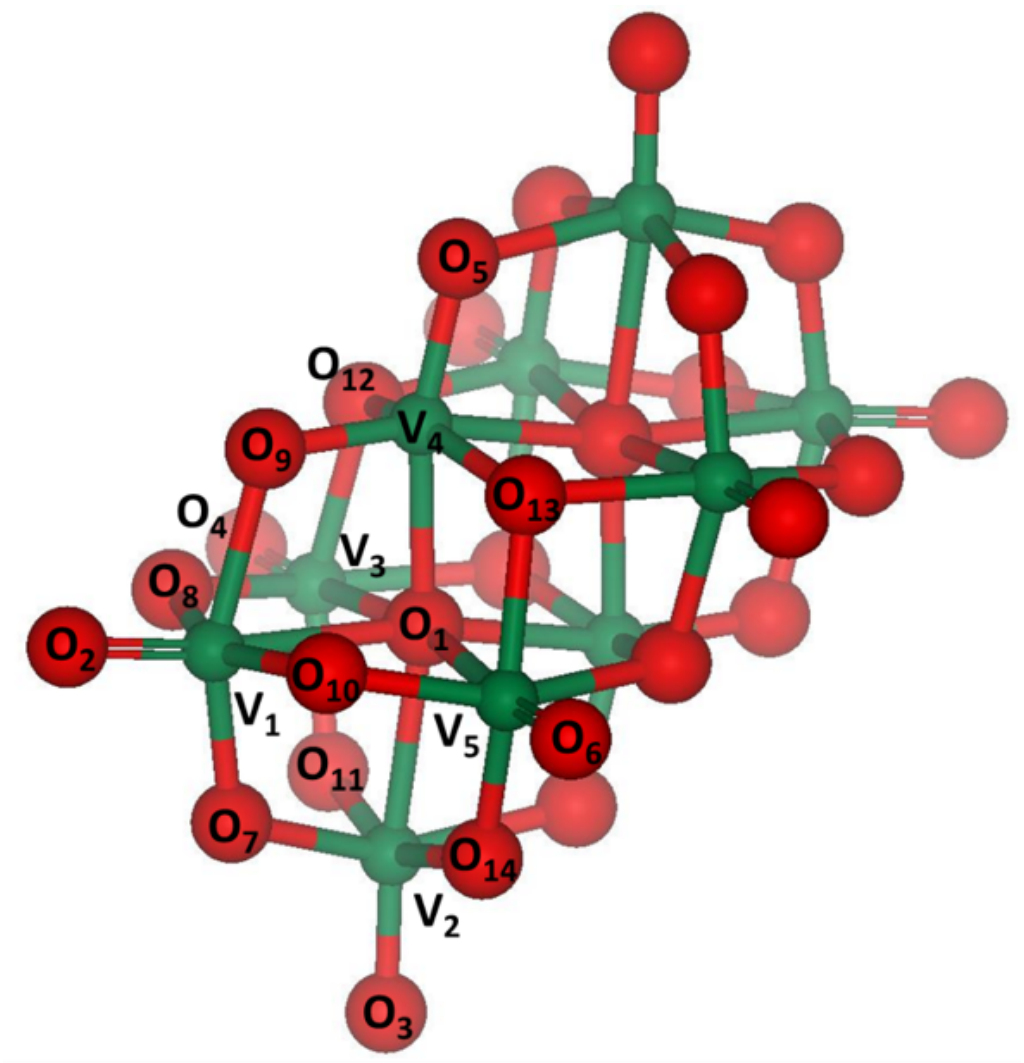

Figure S3. Ball-and-stick representation of $[V_{10}O_{28}]^{6-}$ anion cluster in **1**.

Table S2. V–O bonds lengths in **1**

Bond	Length [Å]
V1-O1	2.2955 (13)
V1-O2	1.6132 (14)
V1-O7	1.8005 (14)
V1-O8	1.8941 (14)
V1-O9	2.0751 (14)
V1-O10	1.8690 (14)
V2-O1	2.3416 (13)
V2-O3	1.6081 (14)
V2-O7	1.8578 (14)
V2-O11	1.8194 (14)
V2-O14	1.9230 (14)
V3-O1	2.2553 (13)
V3-O4	1.6102 (14)
V3-O8	1.8088 (14)
V3-O11	1.8564 (14)
V3-O12	1.9737 (13)
V4-O1	2.0835 (13)
V4-O5	1.6977 (14)
V4-O9	1.6795 (13)

V4-O12	1.9506 (14)
V4-O13	1.9407 (13)
V5-O1	2.2413 (13)
V5-O6	1.6210 (14)
V5-O10	1.8592 (13)
V5-O13	2.0304 (13)
V5-O14	1.7824 (14)

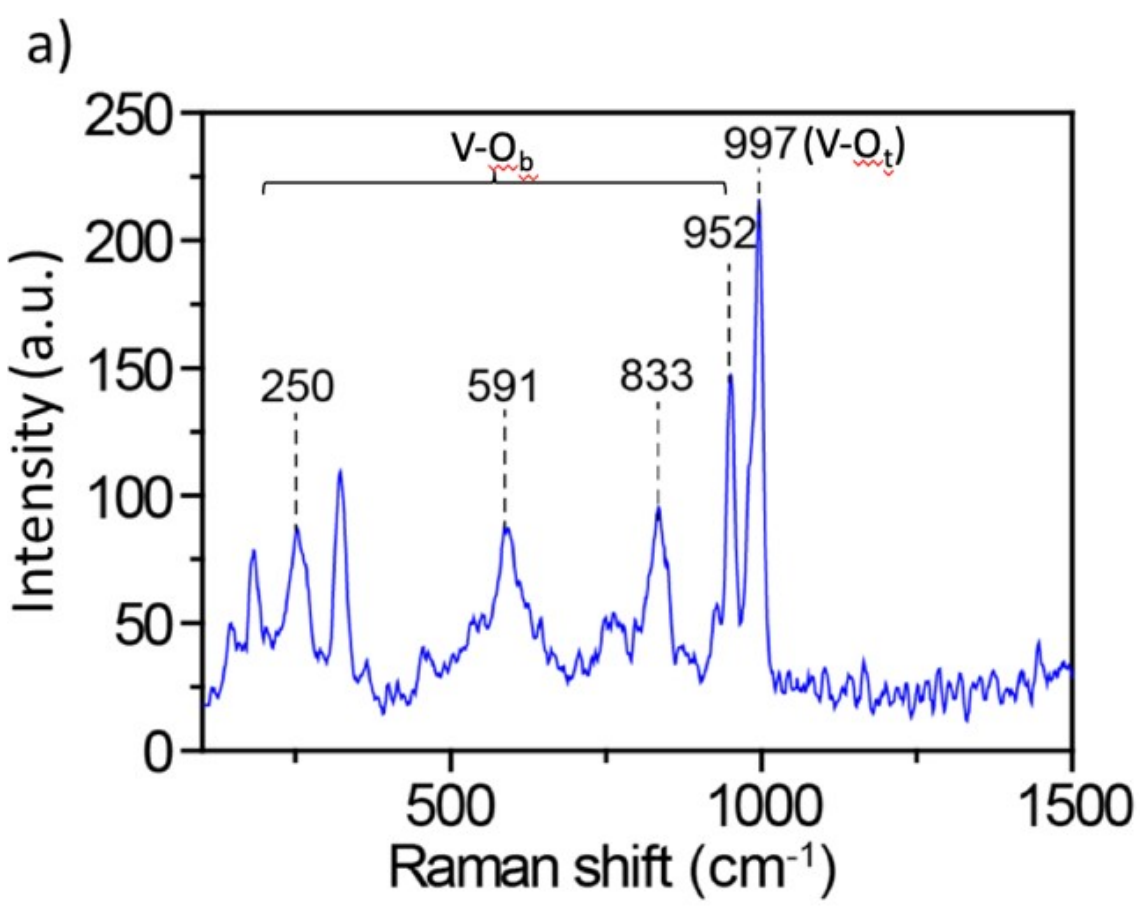

Figure S4. a) Raman spectrum of **1** showing the typical peaks of the $[V_{10}O_{28}]^{6-}$ anion corresponding to V–O vibrations.[4] b) FT-IR measurements of **1**. The bands in the 3500-2000 cm^{-1} range correspond to water molecules and the 1050 (s) cm^{-1} band can be assigned to C-O bonds, which belong to the TRIS component. The peak at 973 (m) belongs to V-O$_t$ bond, while the peaks at 938 (s), 927 (s), 815 (m), 727 (m) and 592 (m) correspond to V-O$_b$ bonds in the $[V_{10}O_{28}]^{6-}$ polyoxometalate structure. [4,5]

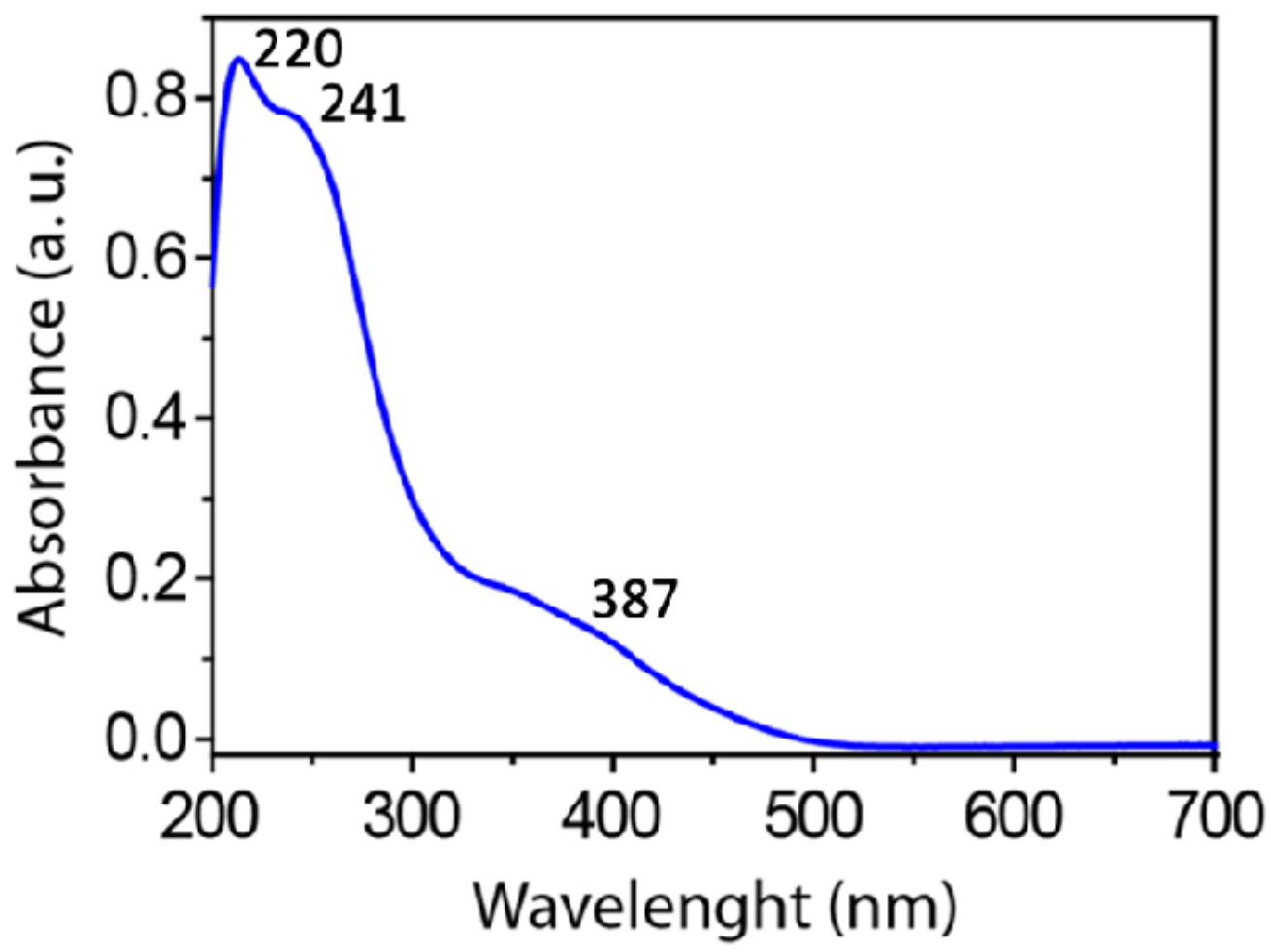

Figure S5. UV-Vis measurements of **1** in water at room temperature. The bands at 387, 241 and 220 nm can be assigned to the decavanadate anion ($[V_{10}O_{28}]^{6-}$) and have been related to the O→V charge transfer transitions.[6]

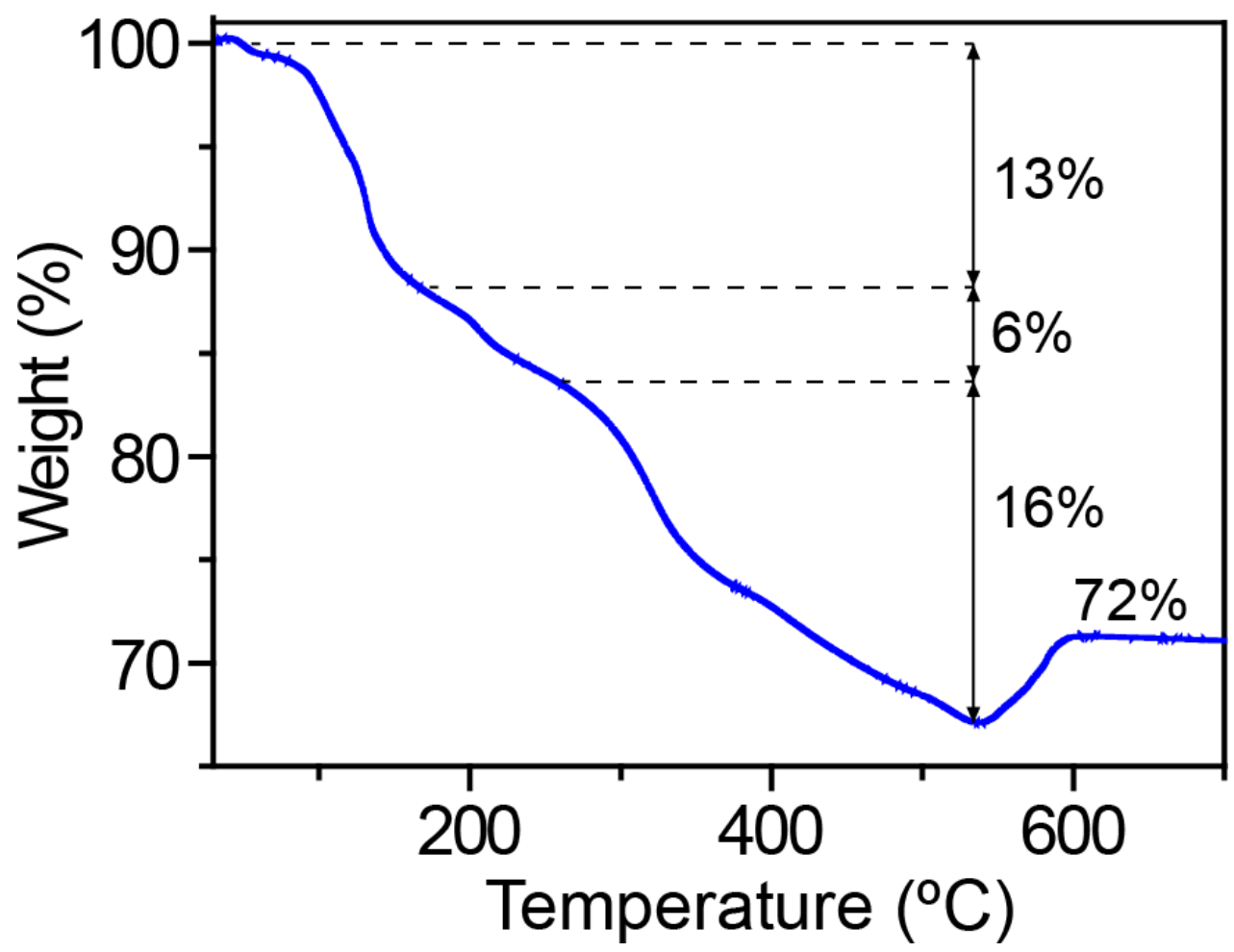

Figure S6. TGA of **1** (5°C/min in air). The weight loss observed below 150°C is attributed to water molecules, while the weight loss between 160°C and 260°C corresponds to Na^+ moieties. The weight loss observed of 16% between 260° and 530°C confirms the presence of the organic moiety ($TRIS^+$). Above 530°C, the residual degraded decavanadate accounts for approximately 72% of the sample.[6]

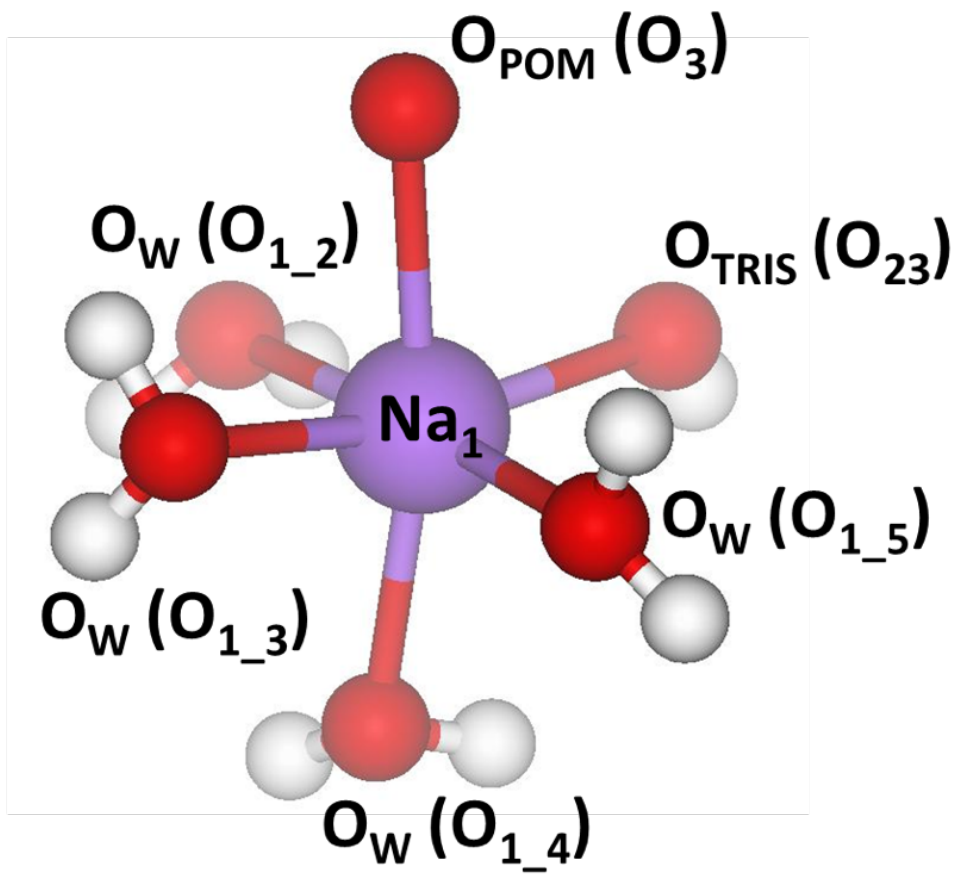

Figure S7. Ball-and-stick representation of the [Na1(μ_1-O TRIS)(μ_1-O POM)($H_2O)_4$] moiety in **1**.

Table S3. Na1–O bond lengths for [Na1(μ_1-O TRIS)(μ_1-O POM)($H_2O)_4$] moiety in **1**.

**Bond**	**Length [Å]**
O3-Na1	2.4863 (17)
O23-Na1	2.4067 (17)
Na1-O1_2	2.4303 (18)
Na1-O1_3	2.4085 (18)
Na1-O1_4	2.376 (2)
Na1-O1_5	2.3725 (18)

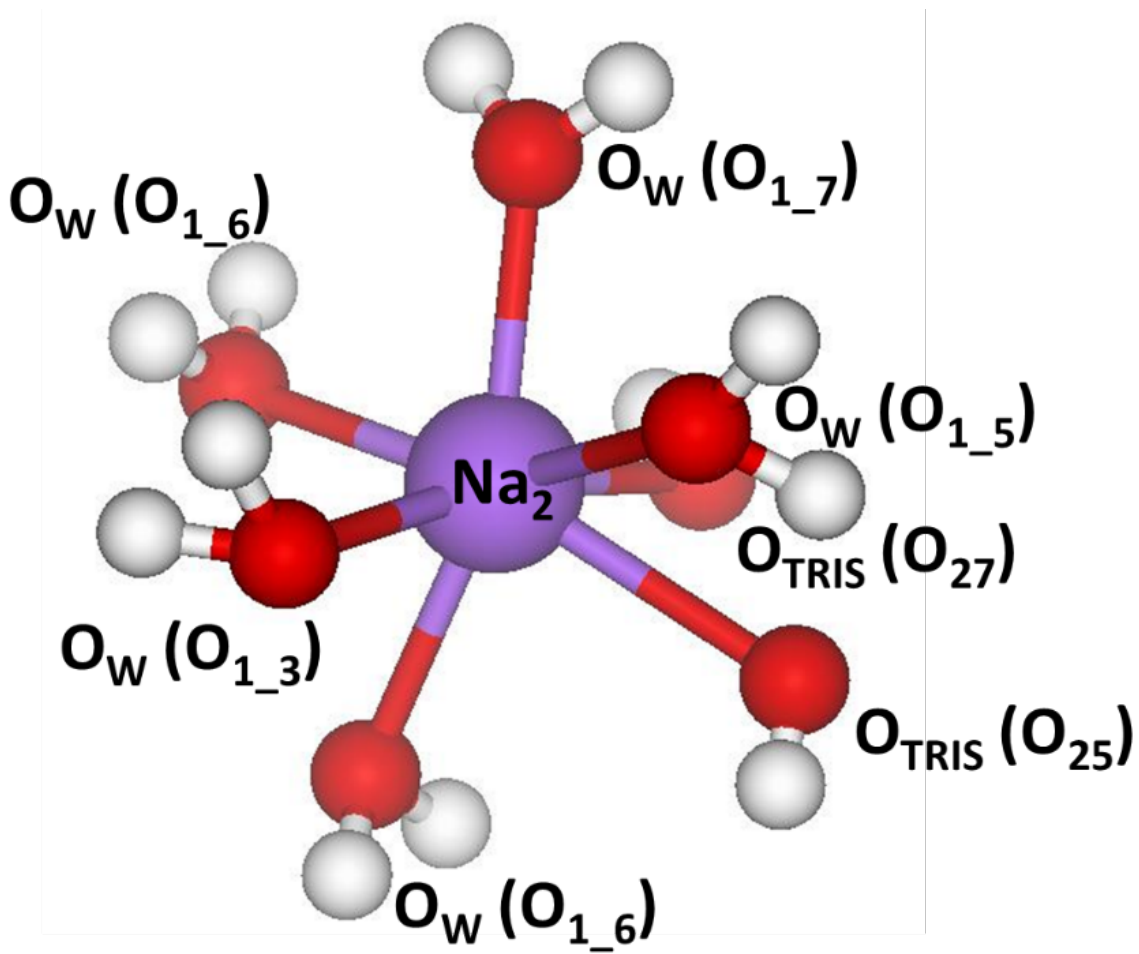

Figure S8. Ball-and-stick representation of [Na2(μ$_2$-O TRIS)(H_2O)$_5$] moiety in **1**.

Table S4. Na2–O bond lengths of [Na2(μ$_2$-O TRIS)(H_2O)$_5$] moiety in **1**.

Bond	Length [Å]
O25-Na2	2.3972 (18)
O27-Na2	2.6491 (17)
Na2-O1_6	2.4408 (19)
Na2-O1_7	2.4121 (18)
Na2-O1_3	2.4683 (18)
Na2-O1_5	2.7744 (19)

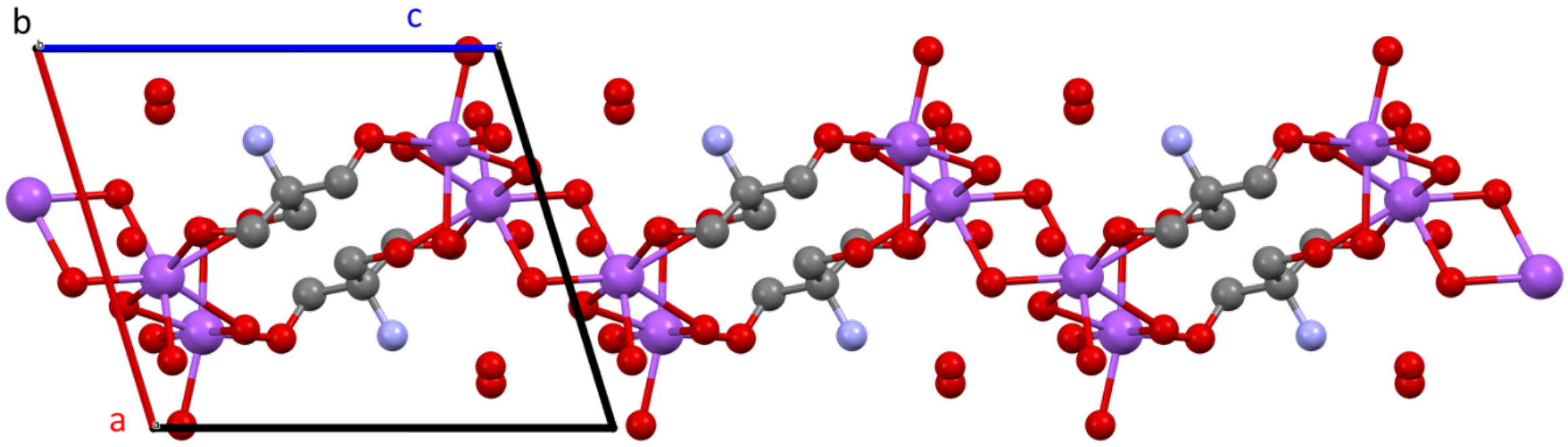

Figure S9. Ball-and-stick representation of a Na-TRIS-Na chain in **1**. Hydrogen atoms are omitted in the figure for clarity.

Table S5. Short distances between Na1 and Na2 within the dimer structure in **1**.

Atom1	Atom2	Distance [Å]
Na1	Na2_i	3.8322 (13)
Na2	Na2_ii	3.8386 (17)

Symmetry operations:
(i) 1-x,-y,1-z; (ii) 1-x,-y,2-z

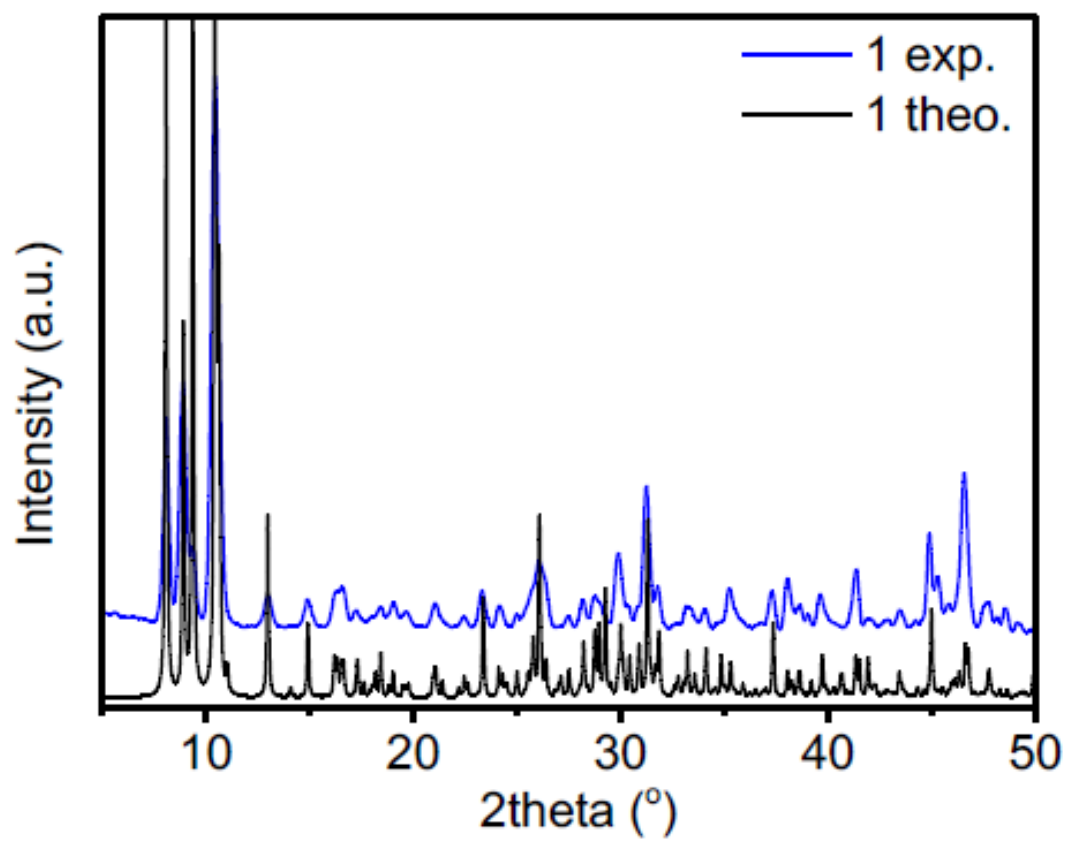

Figure S10. XRD measurements at 100K of a sample of **1**. Blue line corresponds of the experimental XRD measurements of crystals of compound **1**, and black line correspond to the theoretical simulation of the crystals of **1**.

Table S6. The structure of compound **2** was determined by SCXRD. The crystallographic data and structure refinement are shown in this table.

	**2**
**Chemical Formula**	$C_4H_{22}K_2NO_{22}V_5$
**Size (mm)**	0.119 x 0.137 x 0.195
**Formula weight (amu)**	769.12
**Crystal system**	Triclinic
**Space group**	$P\bar{1}$
**a [Å]**	9.60790(10)
**b [Å]**	11.0420(2)
**c [Å]**	11.3028(2)
**α [°]**	109.2809(6)
**β [°]**	104.8997(6)
**γ [°]**	95.8404(6)
**V [Å3]**	1070.66(3)
**Z**	2
**Dx [mg/m3]**	2.386
**Temperature [K]**	100
**Tmax/Tmin**	0.1780/0.0990
**Absorption coeff. [mm^{-1}]**	22.142

**F (000)**	764
**Θ range [°]**	4.33 to 79.08
**Reflections collected**	45978
**Independent reflections**	4610 [R(int) = 0.0480]
**GOF on F2**	1.041
**Final R indices [I > 2σ(I)]**	R1 = 0.0267, wR2 = 0.0719
***R* indices (all data)**	R1 = 0.0275, wR2 = 0.0724

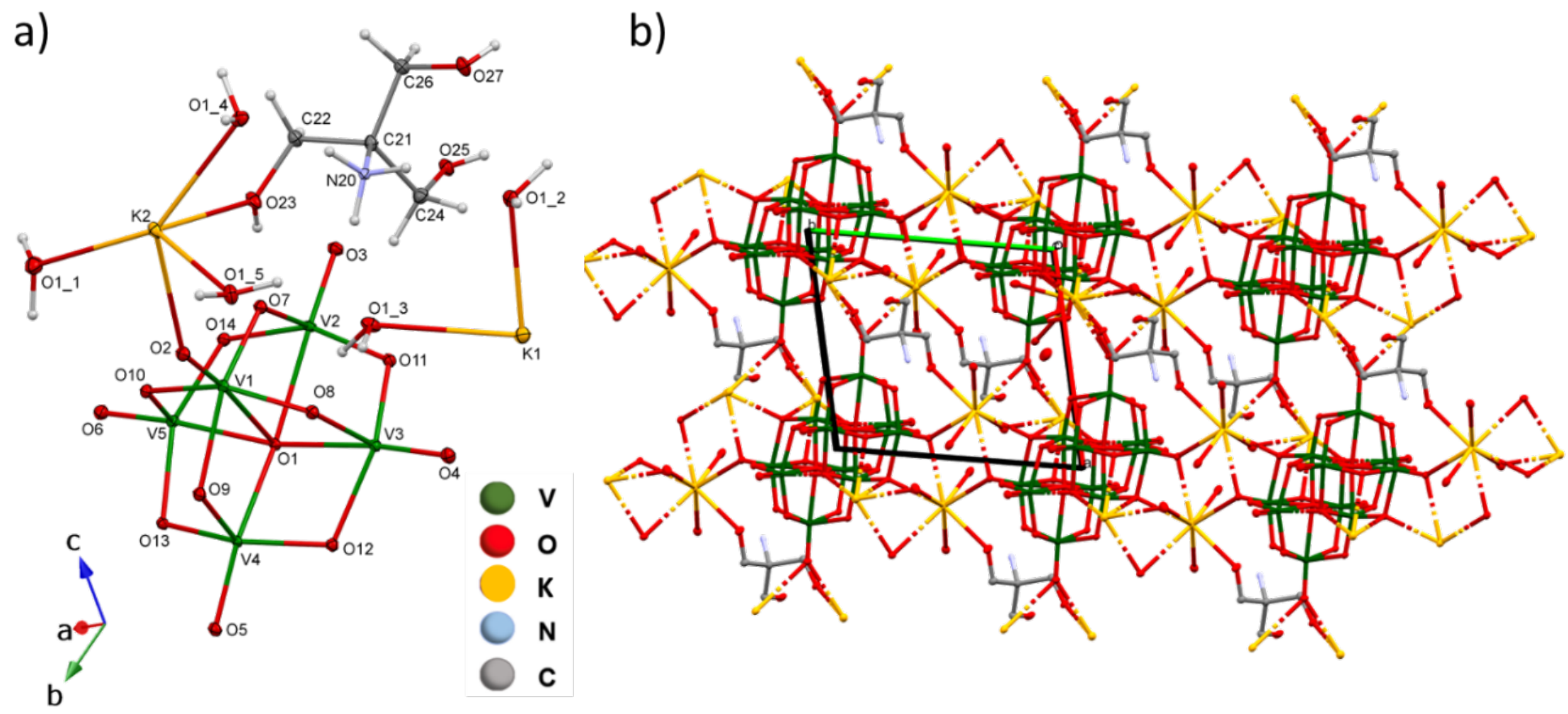

Figure S11. a) ORTEP representation (50% probability) of the crystallographic asymmetric unit of **2**; c) crystal packing of **2** showing $[V_{10}O_{28}]^{6-}$ clusters linked to positively charge adjacent K–TRIS–K chains through oxygen terminal atoms. Hydrogen bonding has been omitted in (c) for clarity.

Table S7. V-O bonds lengths in **2**. Within the $[V_{10}O_{28}]^{6-}$ unit, the vanadium cations are coordinated in an octahedral conformation with an oxidation state of +5 according to the V-O bond lengths found for the oxygen bridges (V-O-V) (2.2913(14)-1.8040(14) Å) and for the typical terminal oxo groups (V=O) (1.7036(14)-1.6072(14) Å).

**Bond**	**Length [Å]**
V1-O1	2.2913(14)
V1-O2	1.6080(14)
V1-O7	1.8245(14)
V1-O8	1.8692(14)
V1-O9	2.0751(14)
V1-O10	1.8867(14)
V2-O1	2.3596(14)
V2-O3	1.6072(14)
V2-O5#2	2.0283(14)
V2-O7	1.8352(14)
V2-O11	1.9008(15)

V2-O14	1.8581(15)
V3-O1	2.2152(14)
V3-O1	2.2152(14)
V3-O8	1.8348(14)
V3-O11	1.7943(14)
V3-O12	2.0276(14)
V4-O1	2.0975(14)
V4-O1#2	2.1418(14)
V4-O5	1.7036(14)
V4-O9	1.6914(14)
V4-O12	1.8852(14)
V4-O13	1.9423(14)
V5-O1	2.2414(14)
V5-O6	1.6134(15)
V5-O10	1.8040(14)
V5-O12#2	2.0140(14)
V5-O13	1.9954(14)

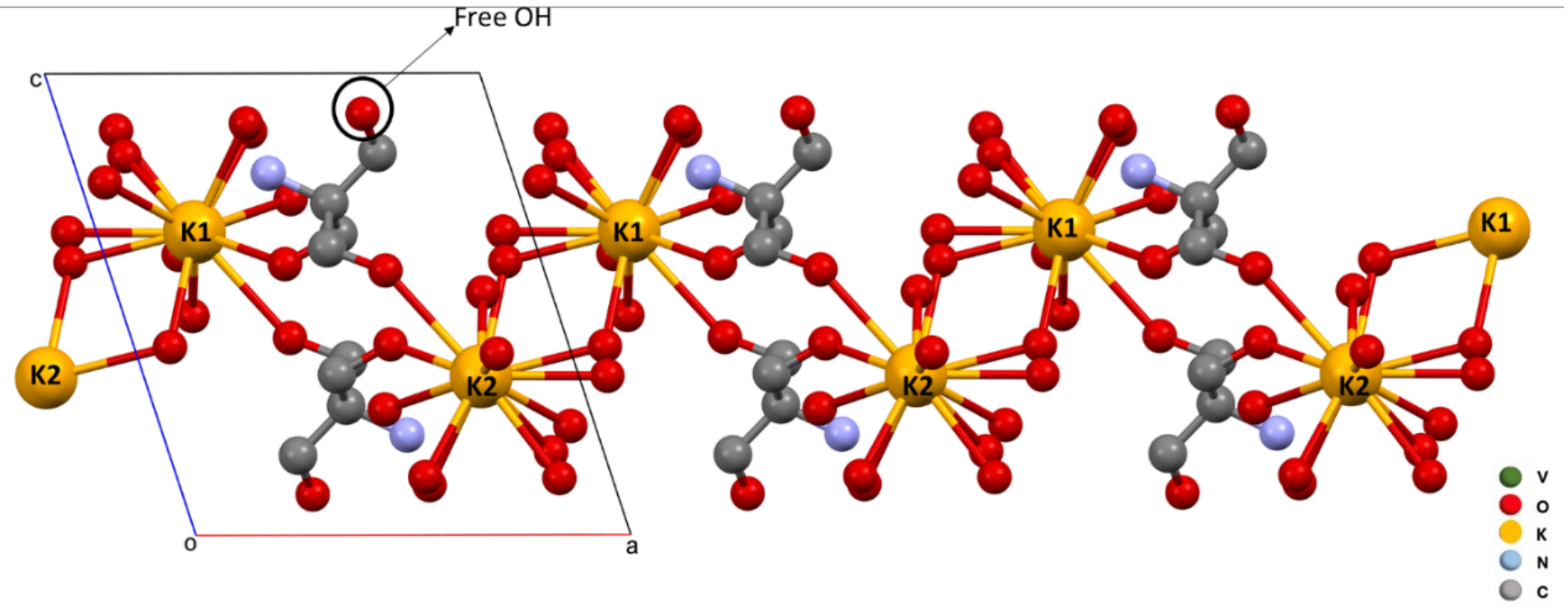

Figure S12. Ball-and-stick representation of a K-TRIS-K chain in **2**. Hydrogen atoms are omitted in the figure for clarity. A free OH molecule can be observed in the TRIS ligand that is not present in compound **1**.

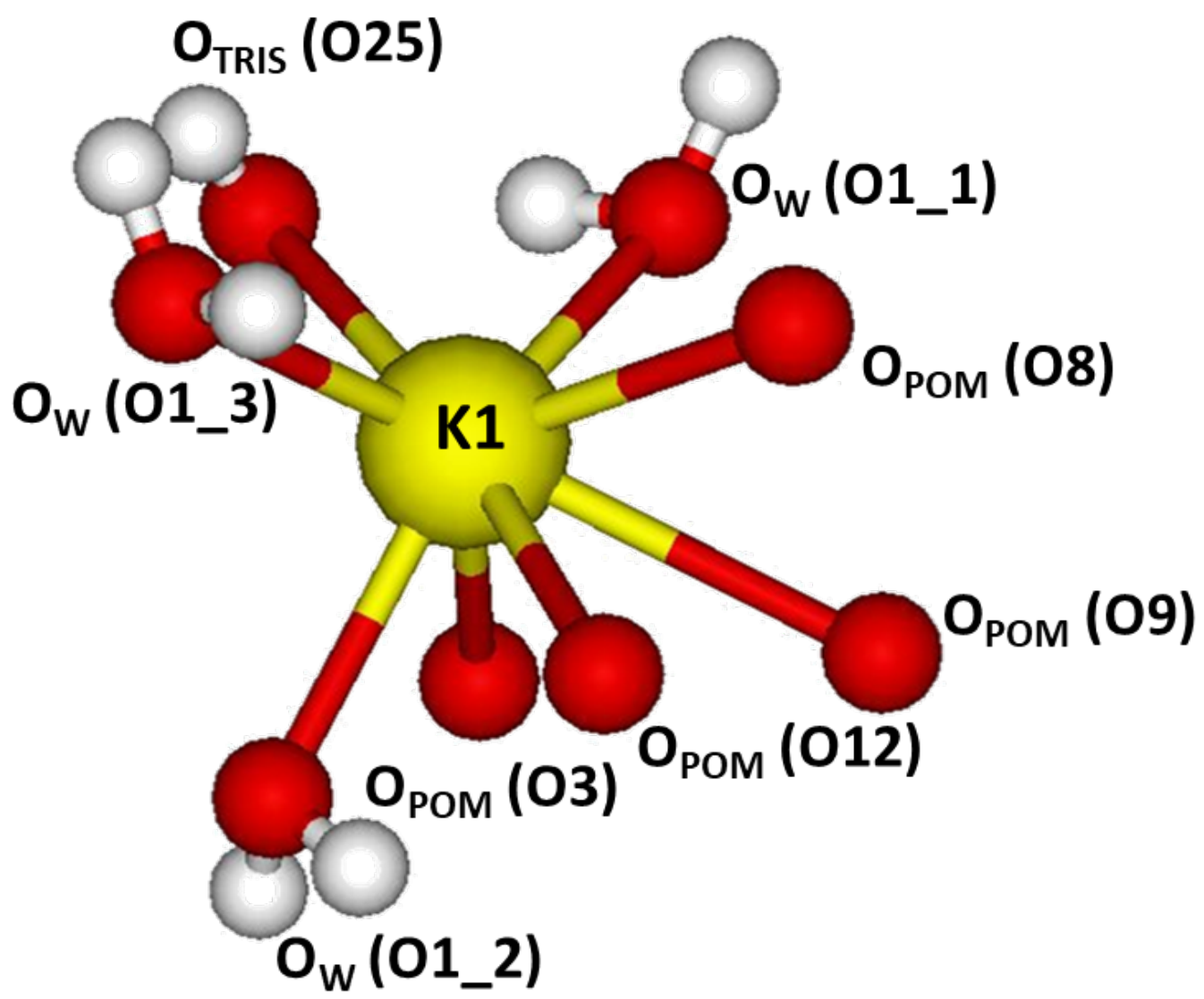

Figure S13. Ball-and-stick representation of the [K1(μ_1-O TRIS)(μ_2-O POM)(μ_1-O POM)$_2$(H_2O)$_3$] moiety in **2**.

Table S8. K1-O bond lengths for [K1(μ_1-O TRIS)(μ_2-O POM)(μ_1-O POM)$_2$(H_2O)$_3$] moiety in **2**.

Bond	Length [Å]
O12-K1#3	2.8860(14)
O3-K1#5	2.8869(15)
O8-K1#3	2.8270(14)
O9-K1#3	3.1041(15)
O25-K1#5	3.1332(17)
K1-O1_1#1	2.7038(17)
K1-O1_2	2.7007(16)
K1-O1_3	2.8201(17)

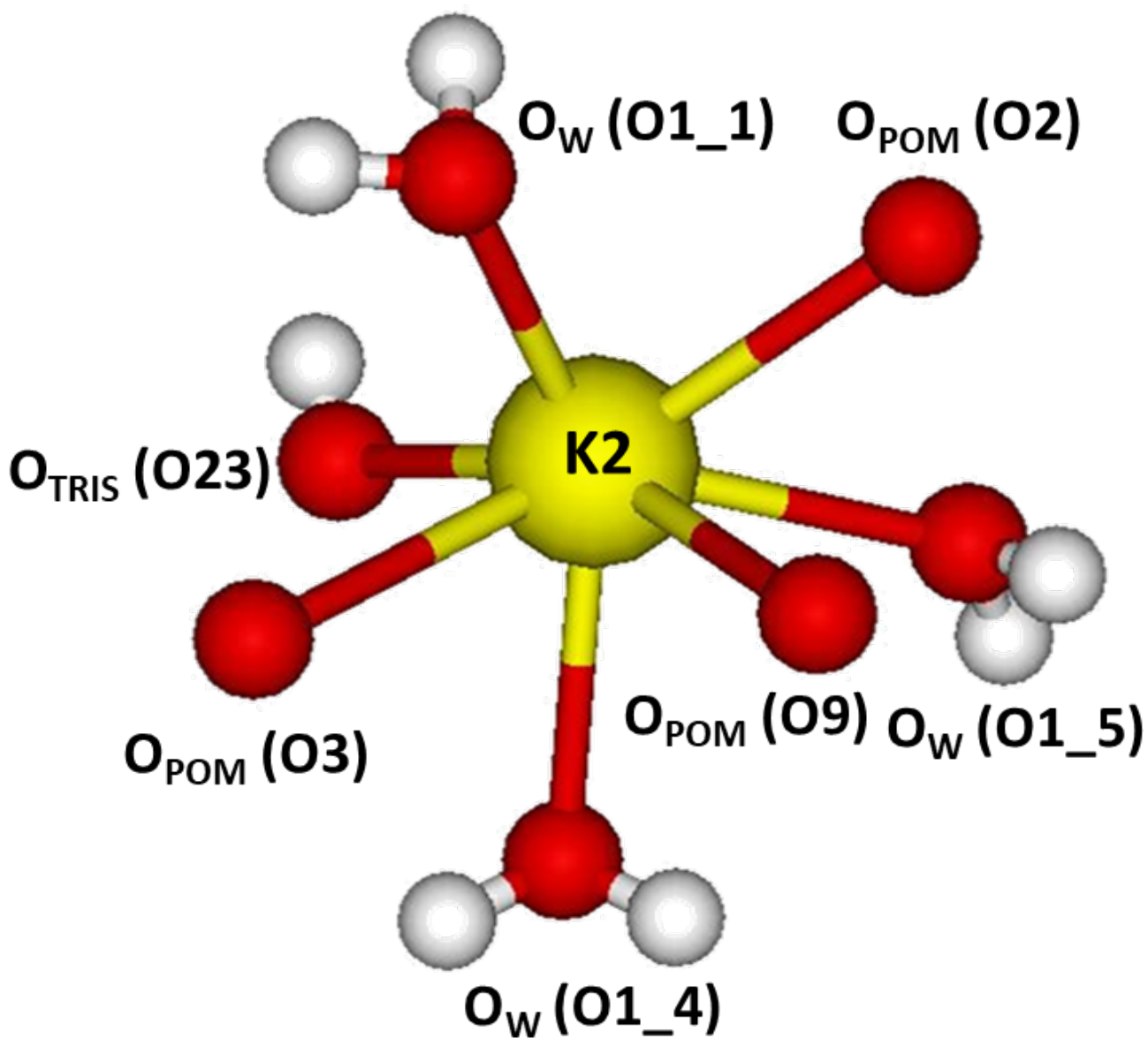

Figure S14. Ball-and-stick representation of the [K2(μ_1-O TRIS)(μ_1-O POM)$_3$(H_2O)$_3$] moiety in **2**.

Table S9. K2-O bond lengths for [K2(μ_1-O TRIS)(μ_1-O POM)$_3$(H_2O)$_3$] moiety in **2**.

Bond	Length [Å]
O3-K2#6	3.0867(15)
O9-K2#4	2.9156(14)
K2-O1_5	2.7646(16)
O2-K2	2.8404(15)
O23-K2	2.6861(15)
K2-O1_1	2.6965(17)
K2-O1_4	2.7886(16)

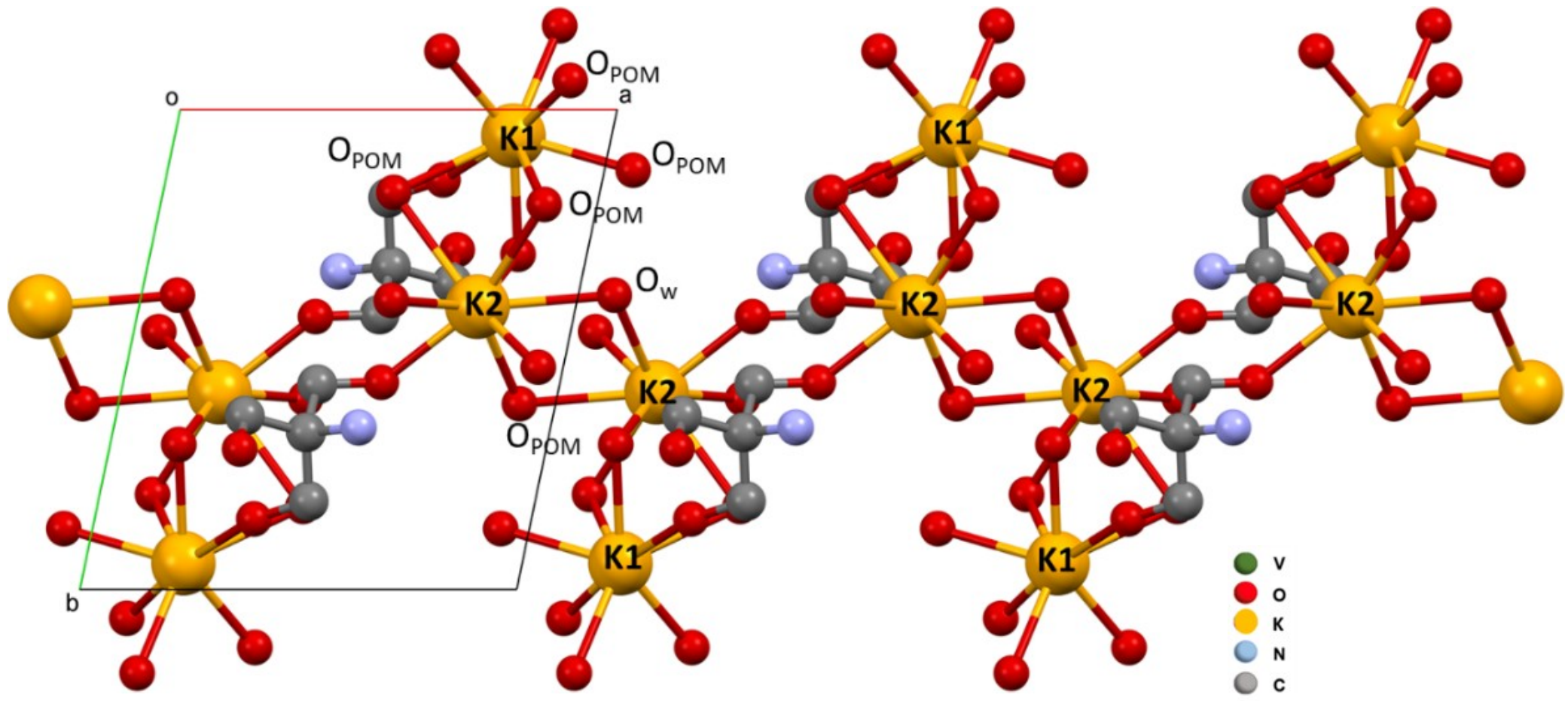

Figure S15. Ball-and-stick representation of a K-TRIS-K chain in **2**. Hydrogen atoms and POM are omitted in the figure for clarity.

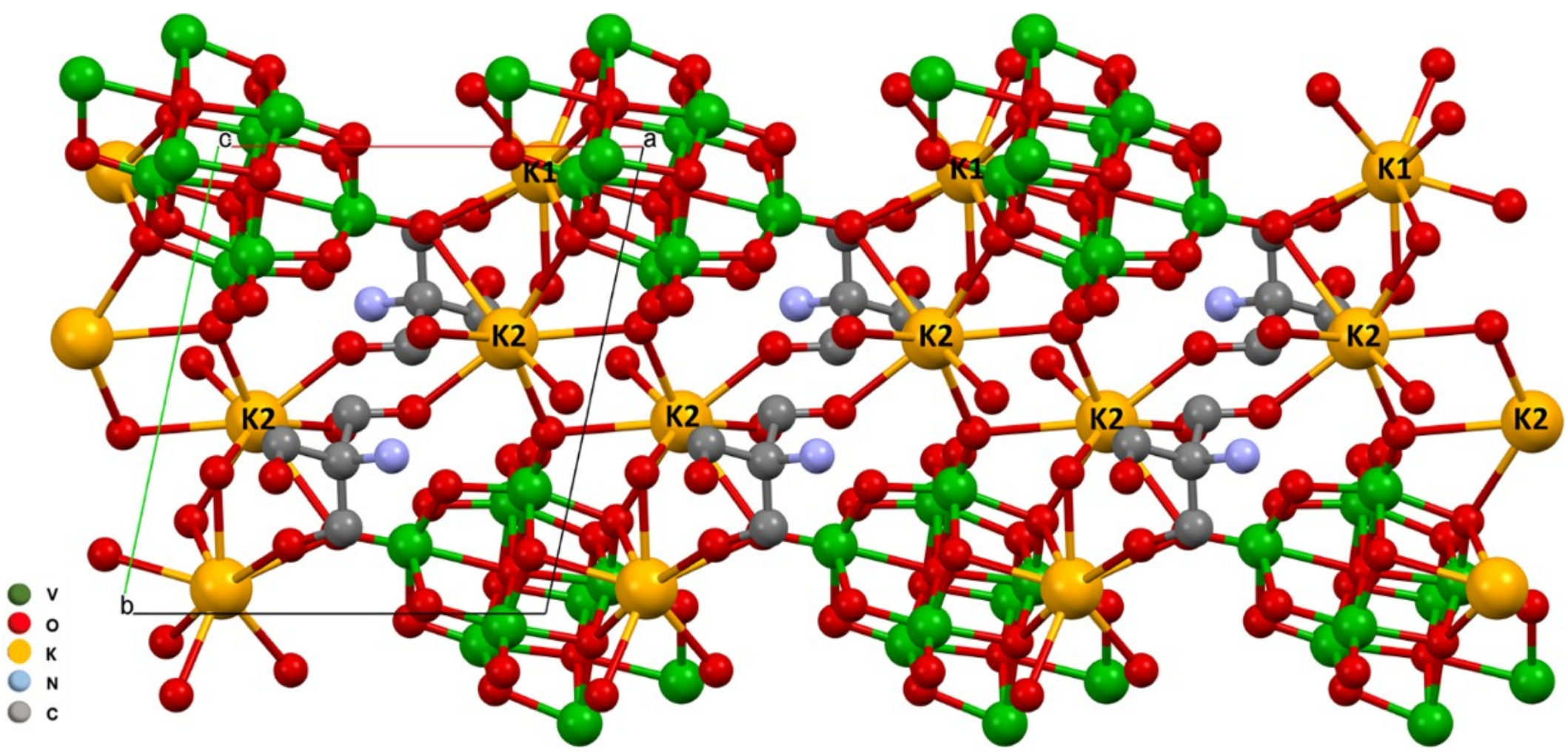

Figure S16. Ball-and-stick representation of a K-TRIS-K chain in **2**. Hydrogen atoms are omitted in the figure for clarity.

Table S10. Short distances between K1 and K2 within the dimer structure in **2**. It can be noted that the distances are longer than in compound **1**.

Atom1	Atom2	Distance [Å]
K1	K2#2	3.9485 (6)
K2	K2#4	4.5885 (9)

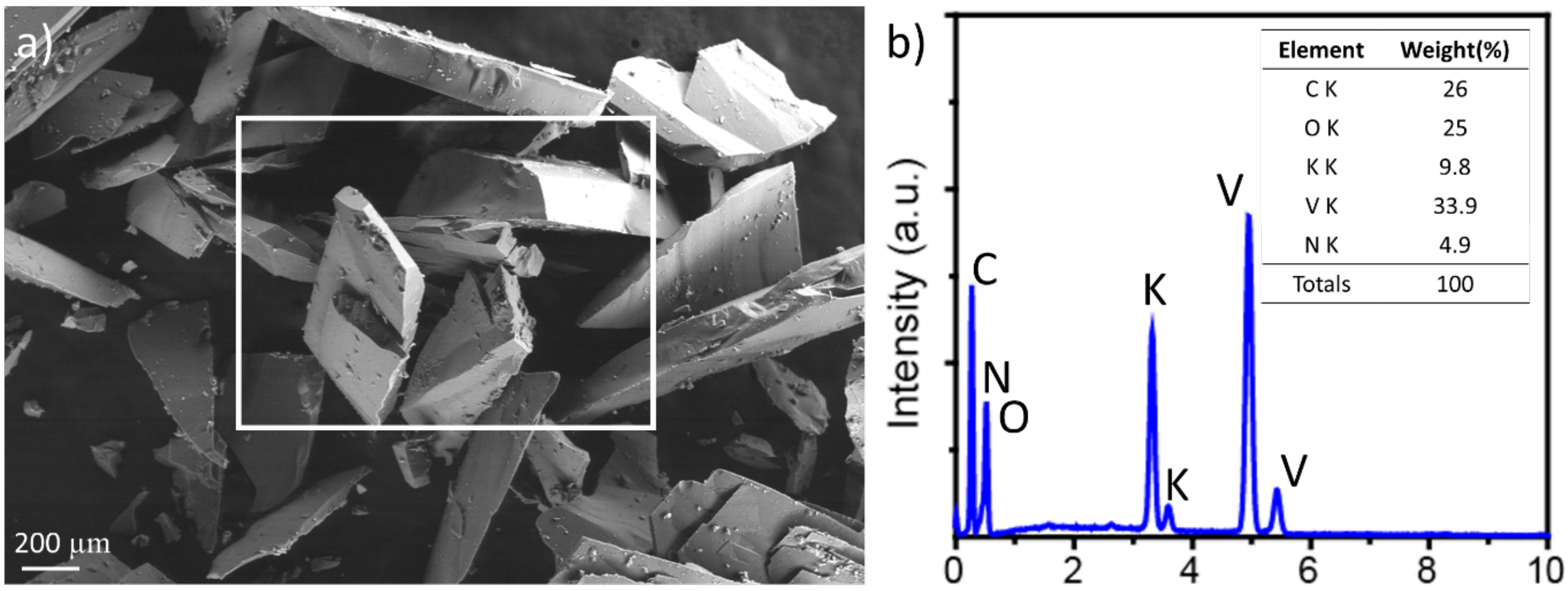

Figure S17. a) SEM images acquired at 20 kV and b) EDX spectrum of a selected area from (a) for the crystals obtained in the synthesis of compound **2**.

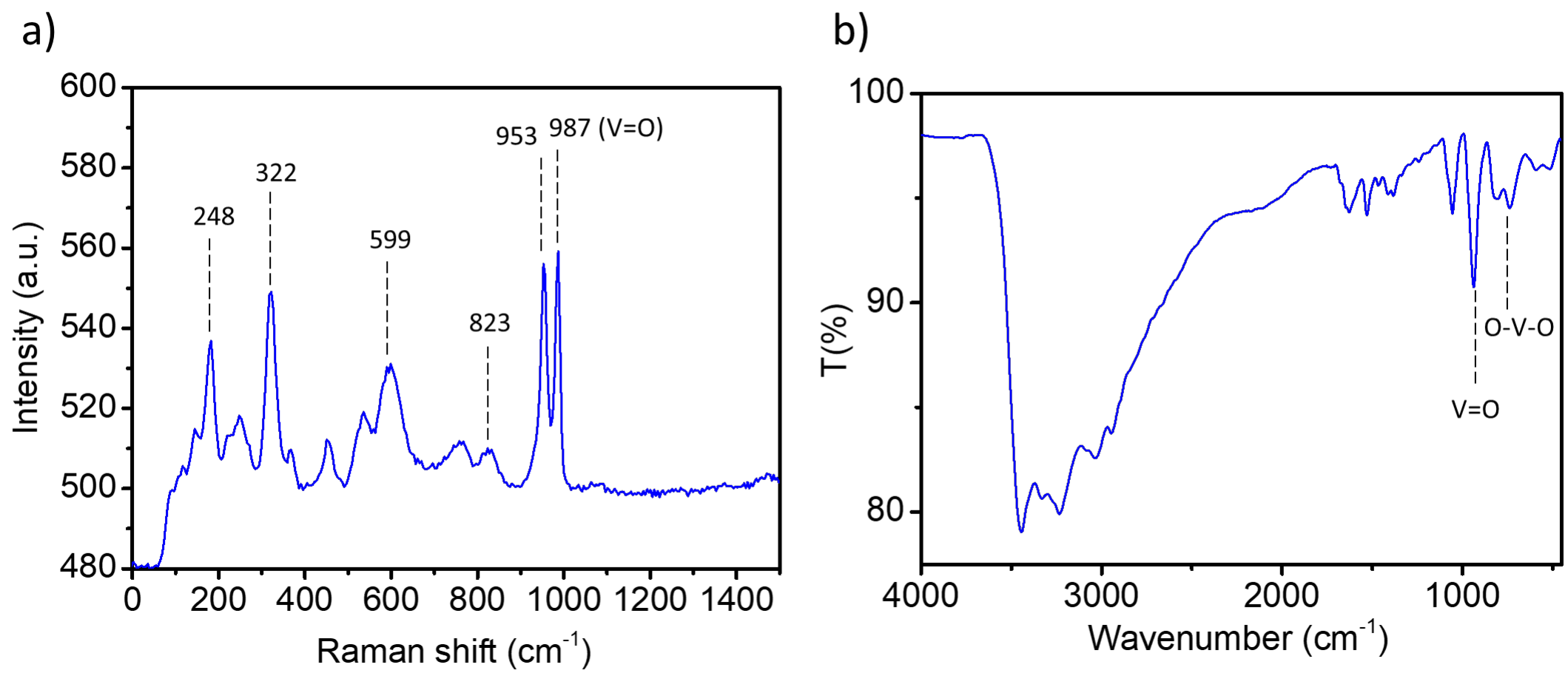

Figure S18. a) Raman measurements of **2** showing the typical peaks of $[V_{10}O_{28}]^{6-}$ corresponding to V-O vibrations. b) FT-IR measurements of **2**. The bands in the 3500-2000 cm^{-1} range correspond to water molecules and the 1056 (m) cm^{-1} band can be assigned to C-O bonds, which belong to the TRIS component. The peak at 938 (m) belongs to V=O bond, while the peaks at 803 (m), 738 (m) and 596 (m) correspond to V-O-V bonds in the $[V_{10}O_{28}]^{6-}$ polyoxometalate structure.[4]

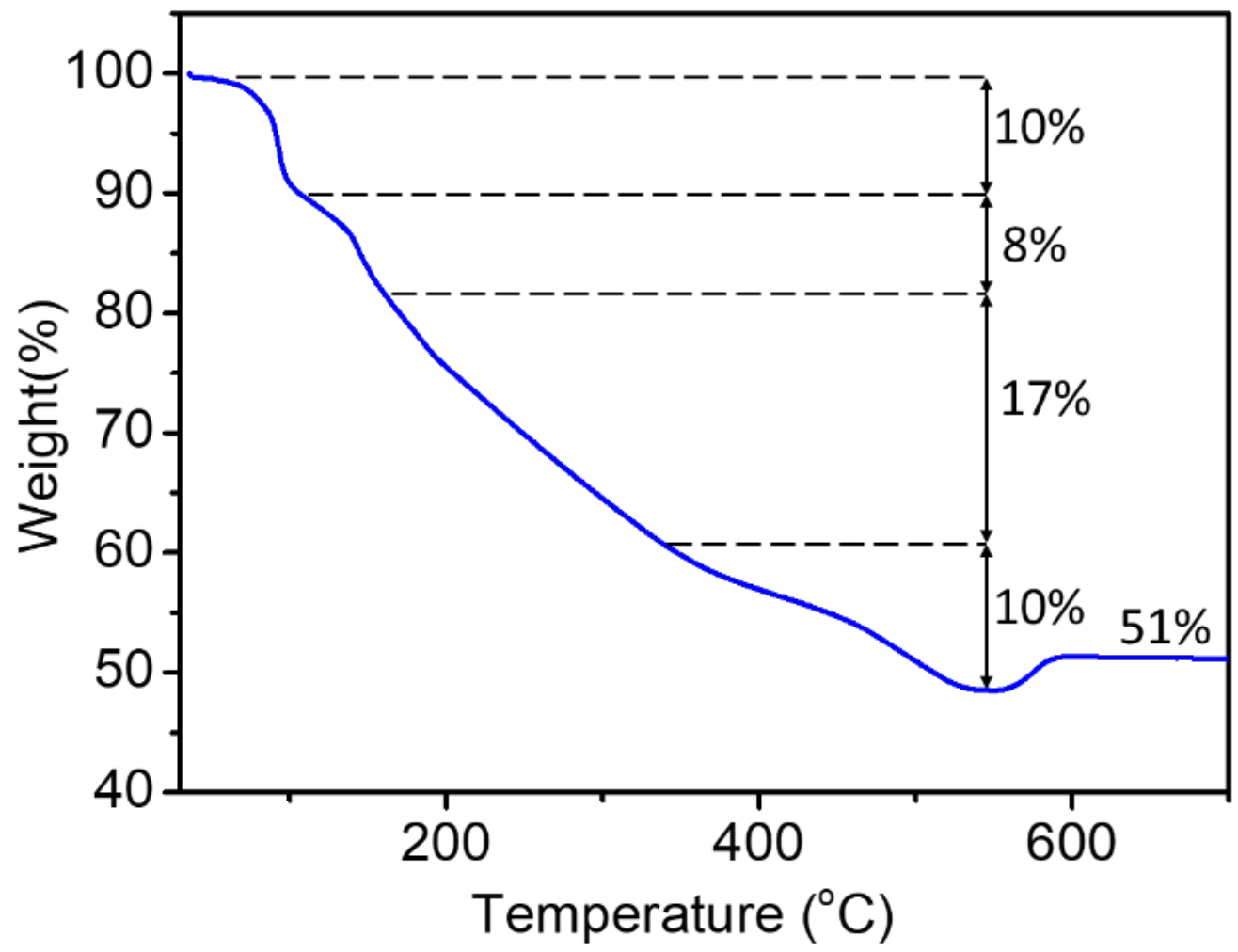

Figure S19. TGA of compound **2** (5°C/min in air). The weight loss observed below 96°C can be assigned to water molecules, while the weight loss between 109°C and 168°C corresponds to the loss of related K^+ moieties. The weight loss observed (16% and 10%) between 168 and 532°C confirms the presence of the organic moiety ($TRIS^+$). Above 532°C, degraded decavanadate remains (51%).[6]

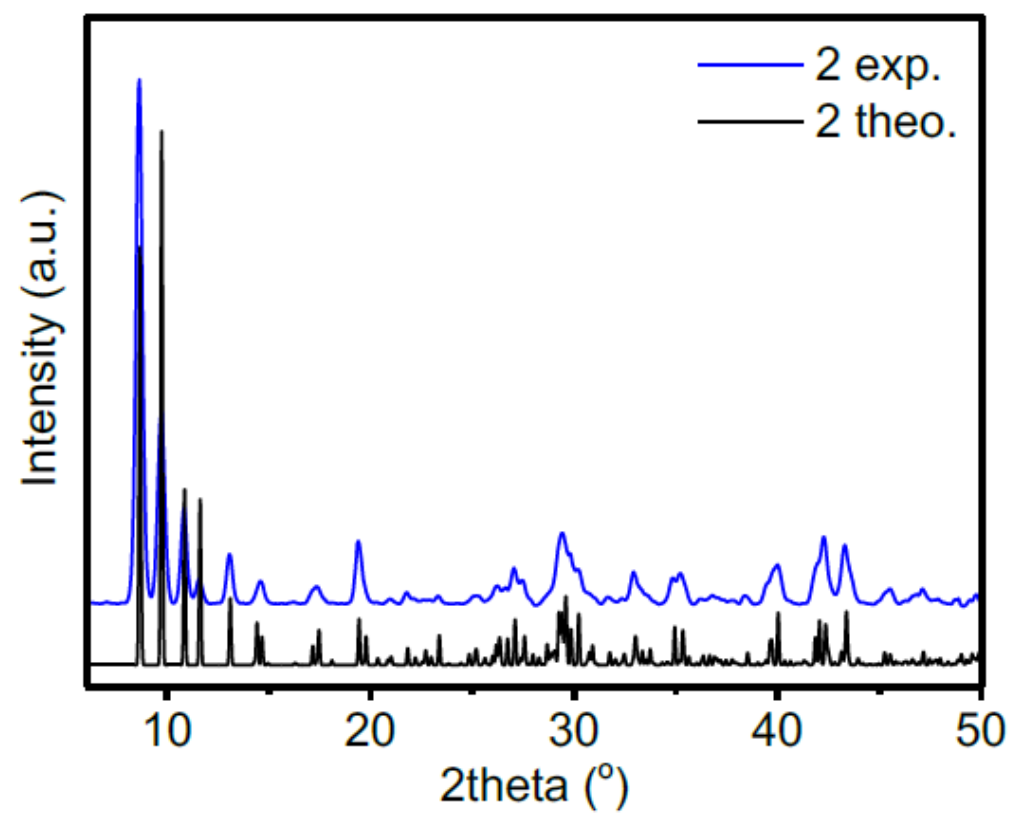

Figure S20. XRD measurements of compound **2** at 100 K. The blue line corresponds to the experimental XRD pattern of compound **2** crystals, while the black line corresponds to the theoretical simulation of the same crystals.

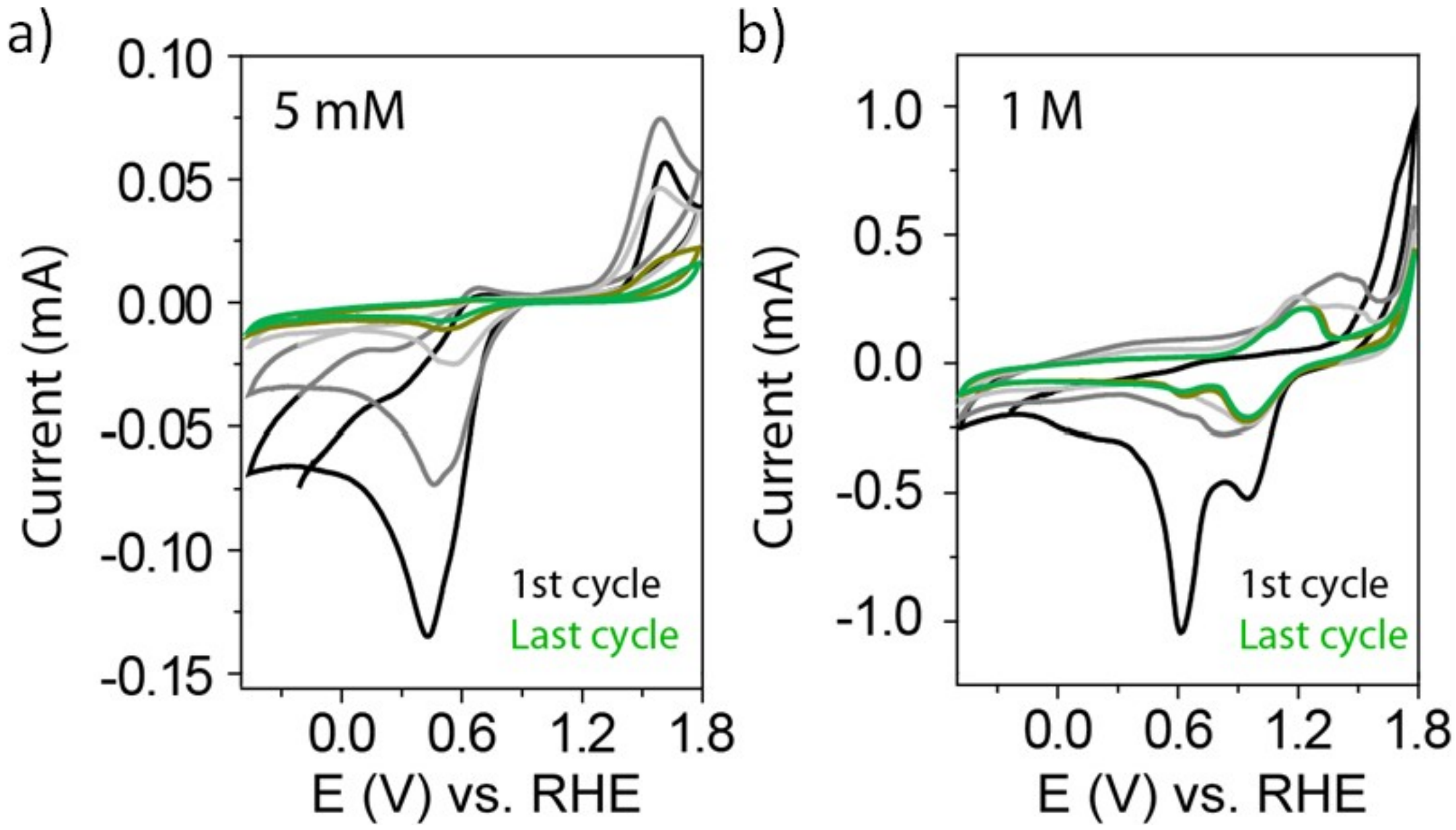

Figure S21. Cyclic voltammogram measurements of **1/CB** under nitrogen atmosphere at 50 mV/s in 5 mM of H_2SO_4 (a) and 1 M H_2SO_4 (b). First cycle is shown in black and the fifth (and last) in green. In a) the peak intensities decrease with cycling, whereas in (b), the signal stabilizes from the third cycle onward. Note that the potentials are referred to the Reversible Hydrogen Electrode (RHE).

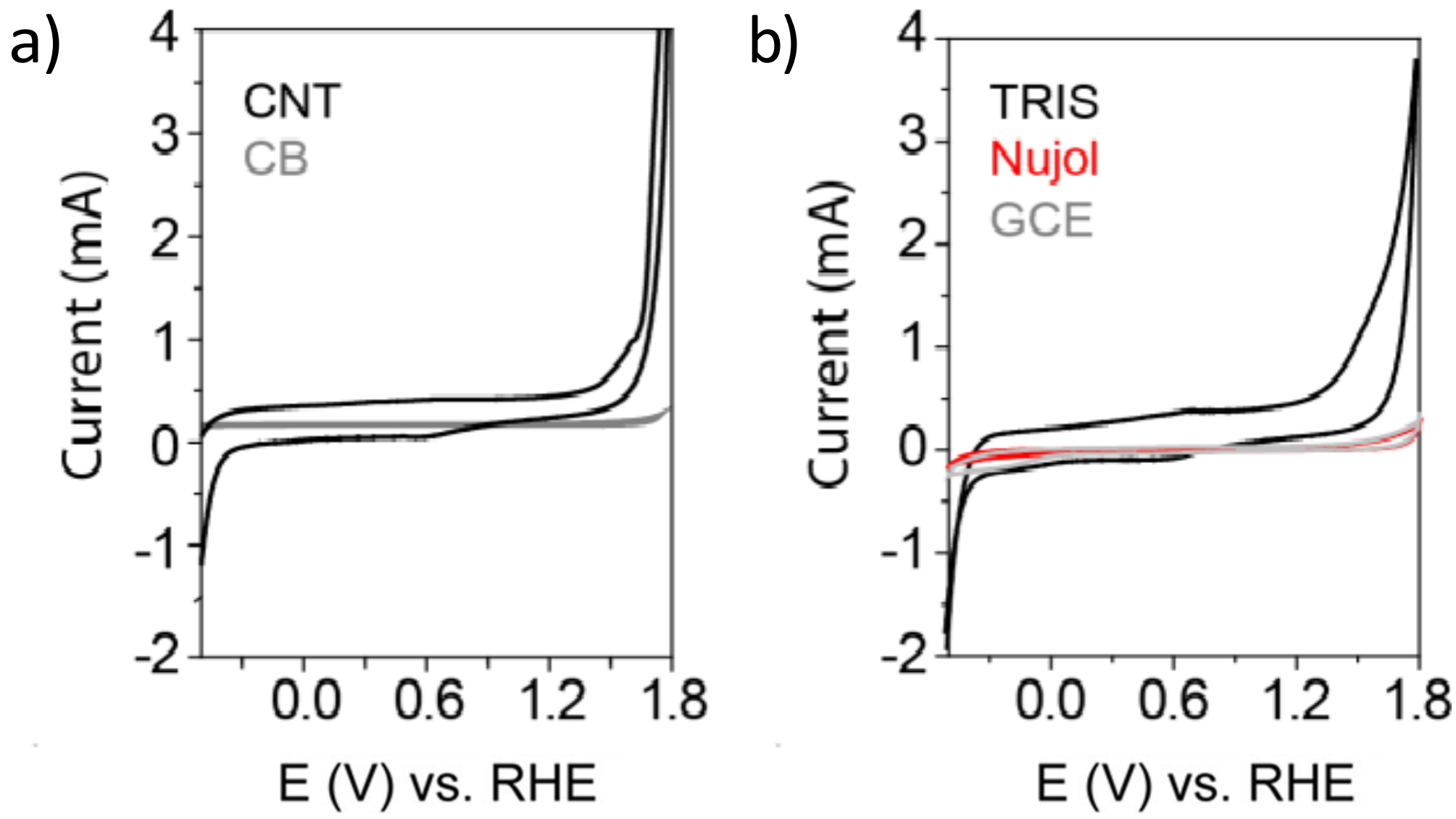

Figure S22. Cyclic voltammogram measurements under nitrogen atmosphere at 50 mV/s in 1M H_2SO_4 of a) CNT and CB and b) TRIS, Nujol and a clean GCE.

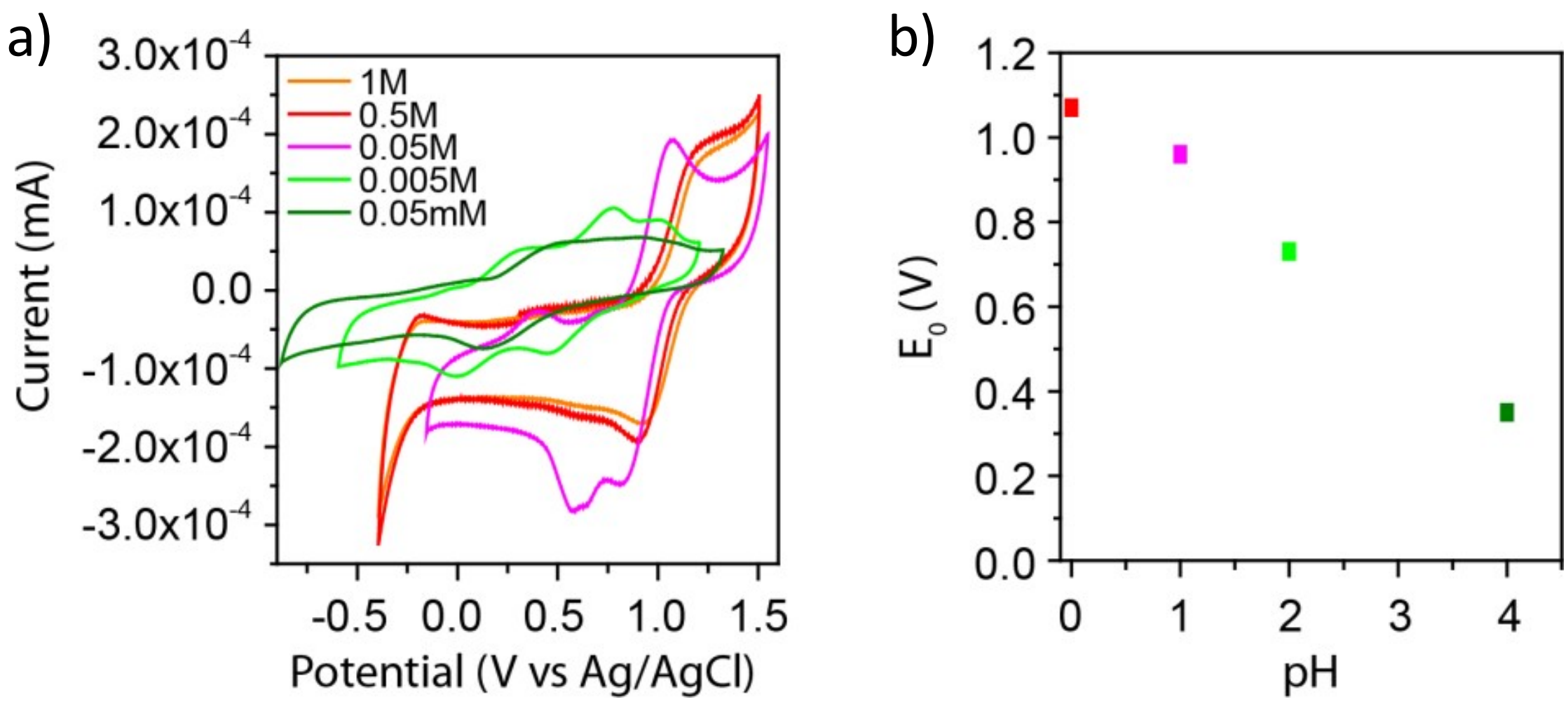

Figure S23. a) Cyclic voltammogram measurements for **1** (1 mM) under nitrogen atmosphere at 50 mV/s in H_2SO_4 aqueous degassed solutions at different concentrations. b) Changes observed in the formal redox potential ($E_0 = (E_a+E_c/2)$) of $[V_{10}O_{28}]^{6-}$ with pH variations indicate that protons are involved in the redox process: $POM^{m-} + ne^- + nH^+$ H $H_nPOM^{(m+n)-}$.[7]

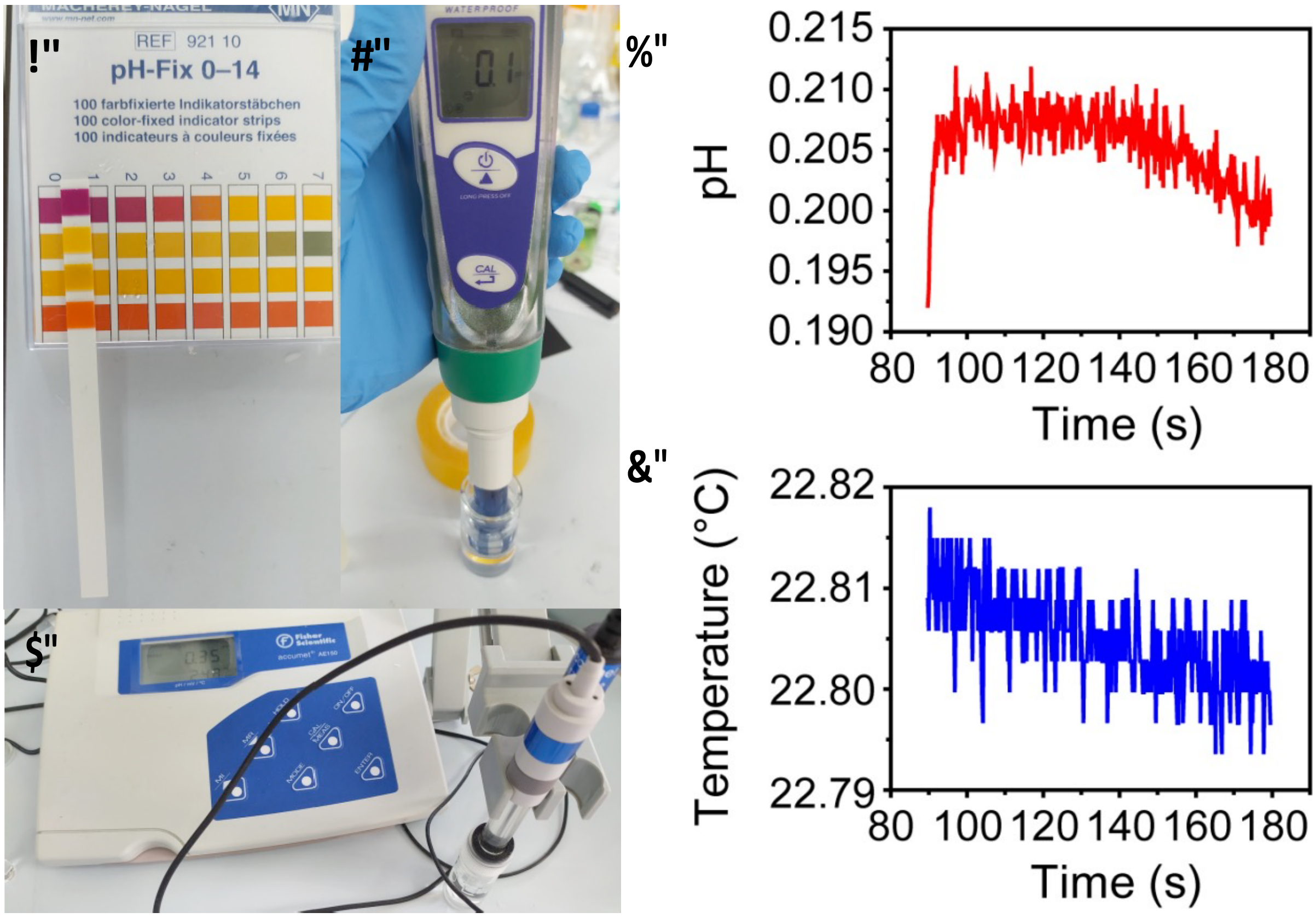

Figure S24. pH measurement of 1 M H_2SO_4 using: a) color-fixed indicator strips; b) and c) two different pH meters (XS Instrument and Fisher, respectively); and d) a Metrohm pH sensor, all indicating that the solution pH is lower than 0.3. In e), the temperature recorded during the electrochemical measurements is shown.

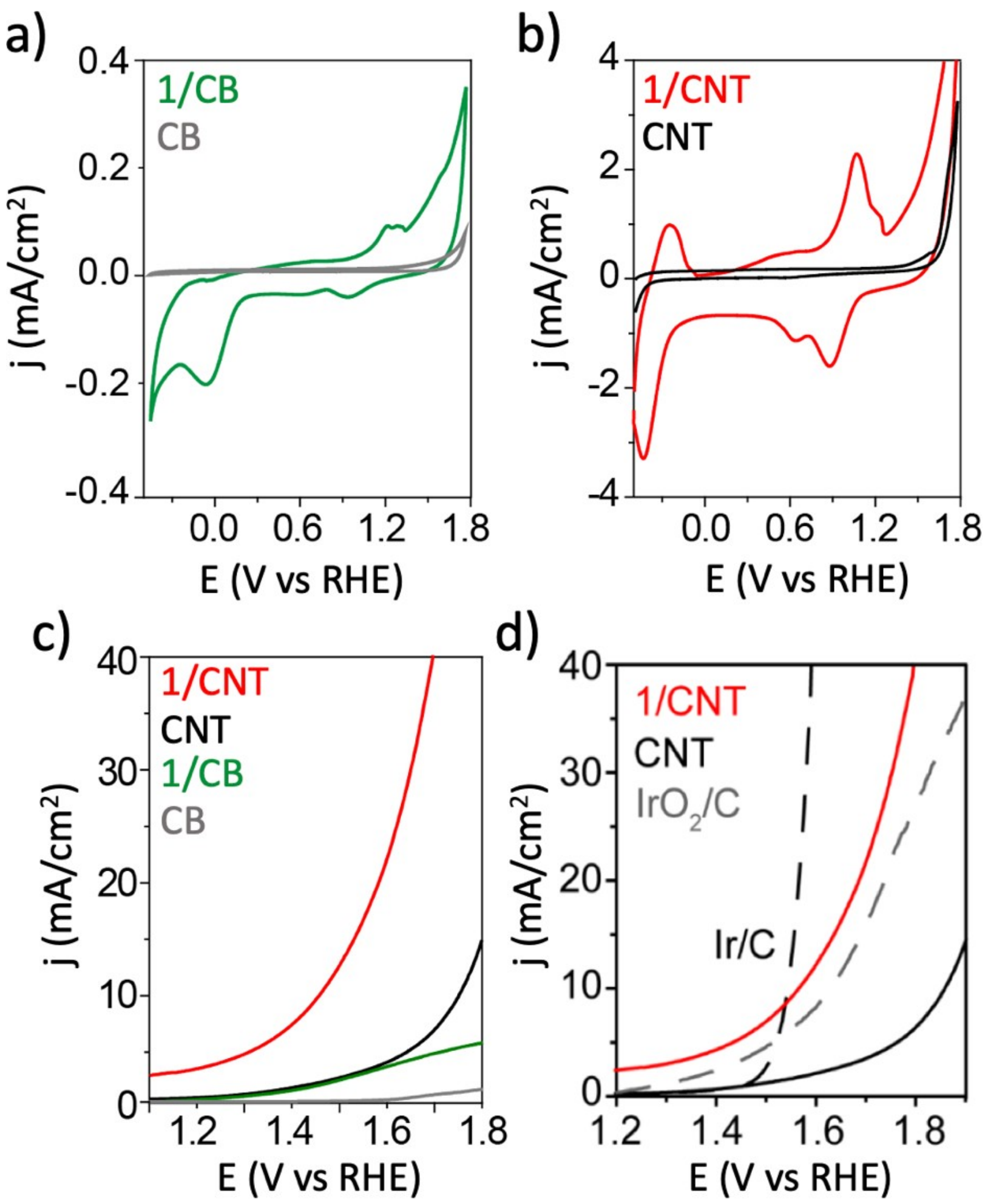

Figure S25. Comparison of the CV measurements under nitrogen atmosphere at 50 mV/s for a) 1/CB (green) *vs.* CB (grey); and b) **1/CNT** (red) *vs.* CNT (black). Comparison of the OER catalytic behaviour of c) **1/CNT** (red), CNT (black), 1/CB (green) and CB (grey) and d) **1/CNT** (red) *vs.* Ir/C (black dashed) and IrO_2/C (grey dashed) as standard electrocatalyst materials. H_2SO_4 (1 M) was used as the electrolyte in all cases. Potentials are referenced to the Reversible Hydrogen Electrode (RHE).

Table S11. Comparative overview for the performance of different POM-based OER electrocatalyst materials, where APS refers to "silane coupling agent", PBA to "Prussian Blue Analogue" and 3a and 3b to ($Co_4Mo_xW_y$).

**Material**	**Electrolyte**	**Onset Potential(V)**	**Overpotential (V) at 10 (mA/cm^2)**	**Tafel Slope (mV/dec)**	**Reference**
1/CNT	1M H_2SO_4	1.45	0.34	360	This work
Ru_4Si_2-APS/NF	0.2M Na_2PO_4	1.75	0.55	172	[8]
PBA@POM	1M KOH	1.52	0.44	23.45	[9]
3a[Cs]	50mM KPi buffer	1.25	0.19	216	[10]
3b[Cs]	50mM KPi buffer	1.25	0.19	228	[10]
Co_4POM	0.1M Na_3PO_4	1.6	0.37	203	[11]
POM/Pd/MoS_2	0.1M NaOH	1.45	0.55	190	[12]
$Fe_4^{III}(H_2O)_2(PW_9O_{34})_2]^{6-}$ (Fe_4)	1M H_2SO_4	*	*	*	[13]
CoW_{12}-based PIC	0.1M H_2SO_4	1.89	0.67	124	[14]

* The values are not provided in numerical form in the publication.

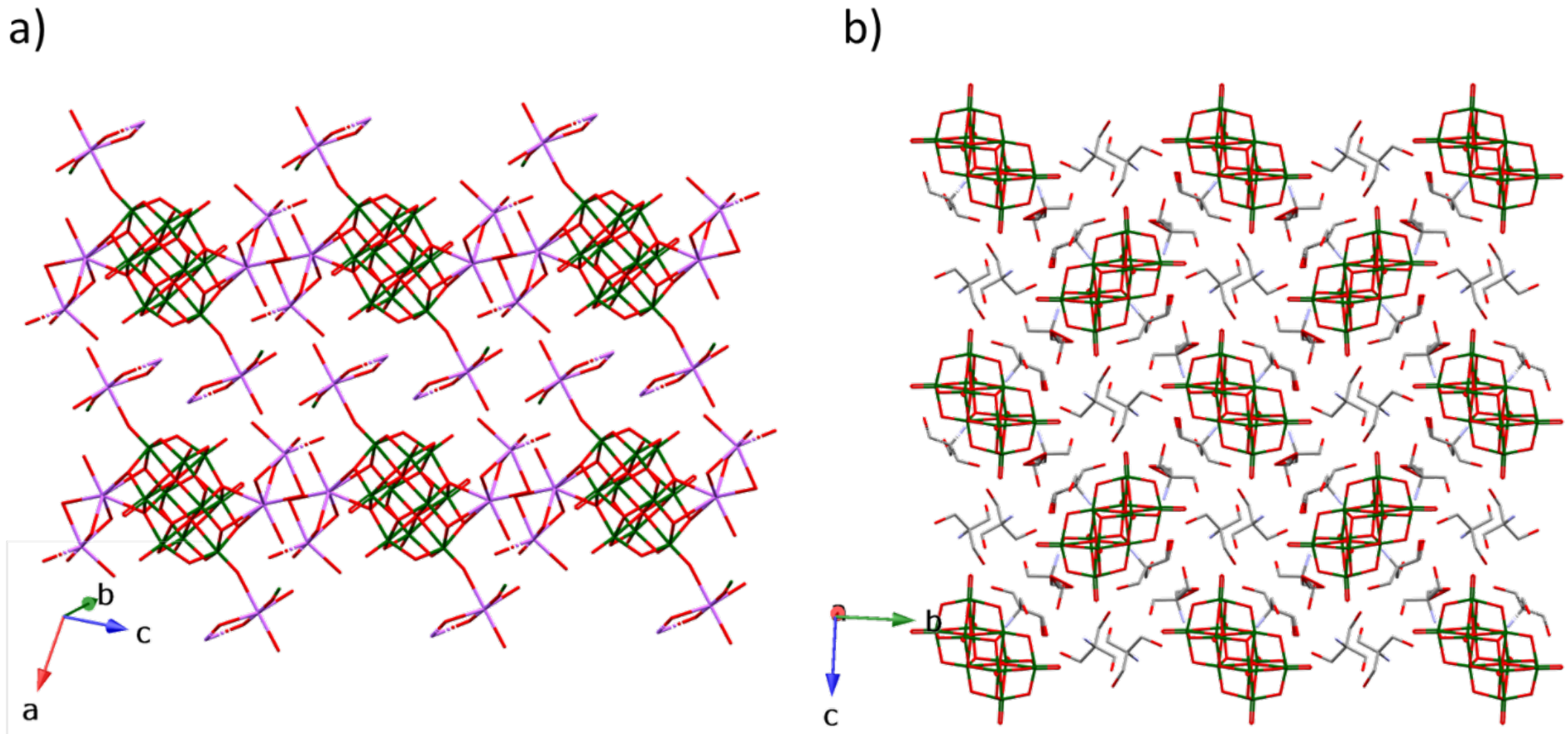

Figure S26. Representation of 3D packing of **a)** Na_6POM and **b)** $TRIS_6POM$. Both structures were obtained from the literature [1, 2]. In the case of (**a**), Na–Na bridges can be observed, whereas in (**b**) the TRIS molecules are isolated.

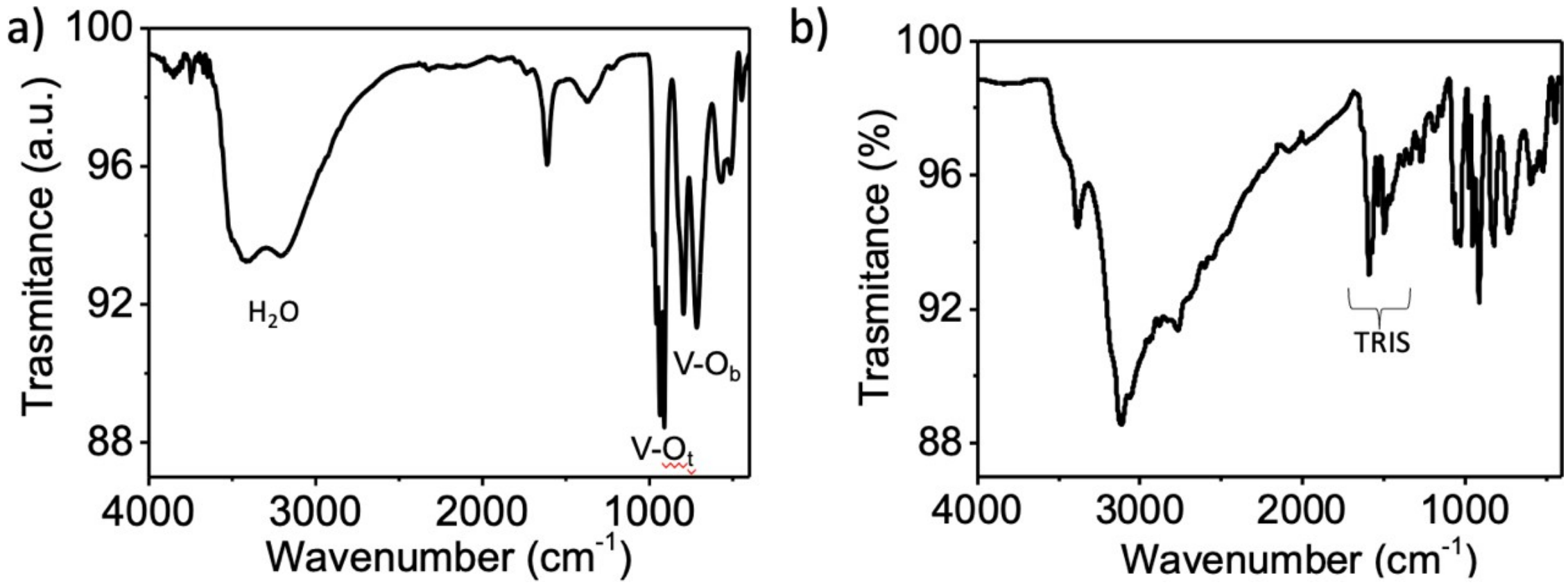

Figure S27. FT-IR measurements of a) Na_6POM and b) $TRIS_6POM$. In (a), the band around 3500 cm^{-1} corresponds to water molecules. The peak at 973 cm^{-1} (medium) is assigned to the V=O bond, while the peaks at 950 (strong), 936 (medium), 880 (medium), and 730 cm^{-1} (medium) correspond to V–O–V bonds in the $[V_{10}O_{28}]^{6-}$ polyoxometalate structure. For (b), the bands match those previously reported [4]

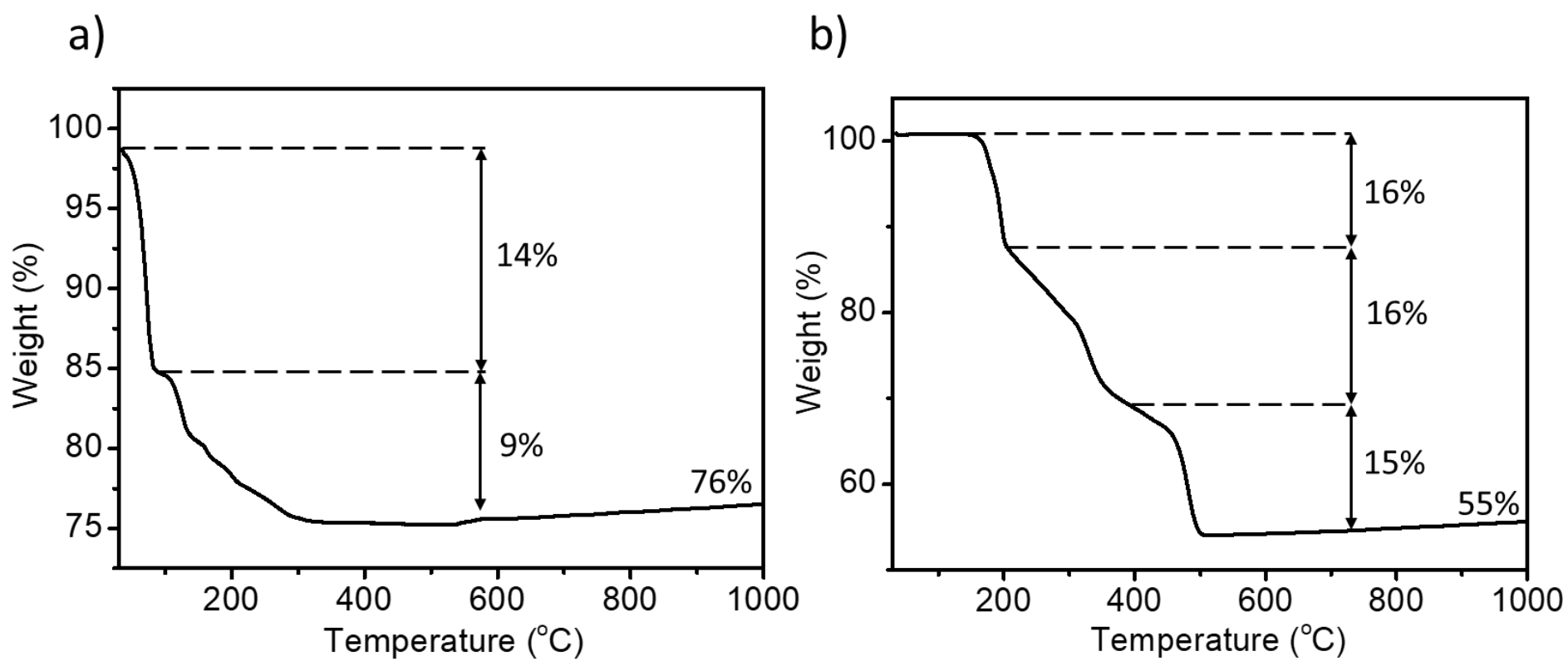

Figure S28. TGA of **a) Na_6POM** and **b) $TRIS_6$POM** (5°C/min in air). In (**a)** the weight loss observed below 50°C can be attributed to water molecules, while the weight loss between 103°C and 314°C corresponds to the release of related Na^+ moieties. Above 324°C, degraded decavanadate remains (76%). In (**b)** the three weight losses observed between 200 and 450°C can be assigned to the degradation of TRIS molecules in three different steps. Above 450°C, degraded decavanadate remains (55% weight).

Table S12. Comparison in M-M distances between **1** and **2**.

Compound	Bond	Distance (Å)	Overpotential (V) @ 10 mA/cm^2
1	Na2-Na2	3.8322 (17)	0.34
2	K2-K2	4.5885 (9)	1.9

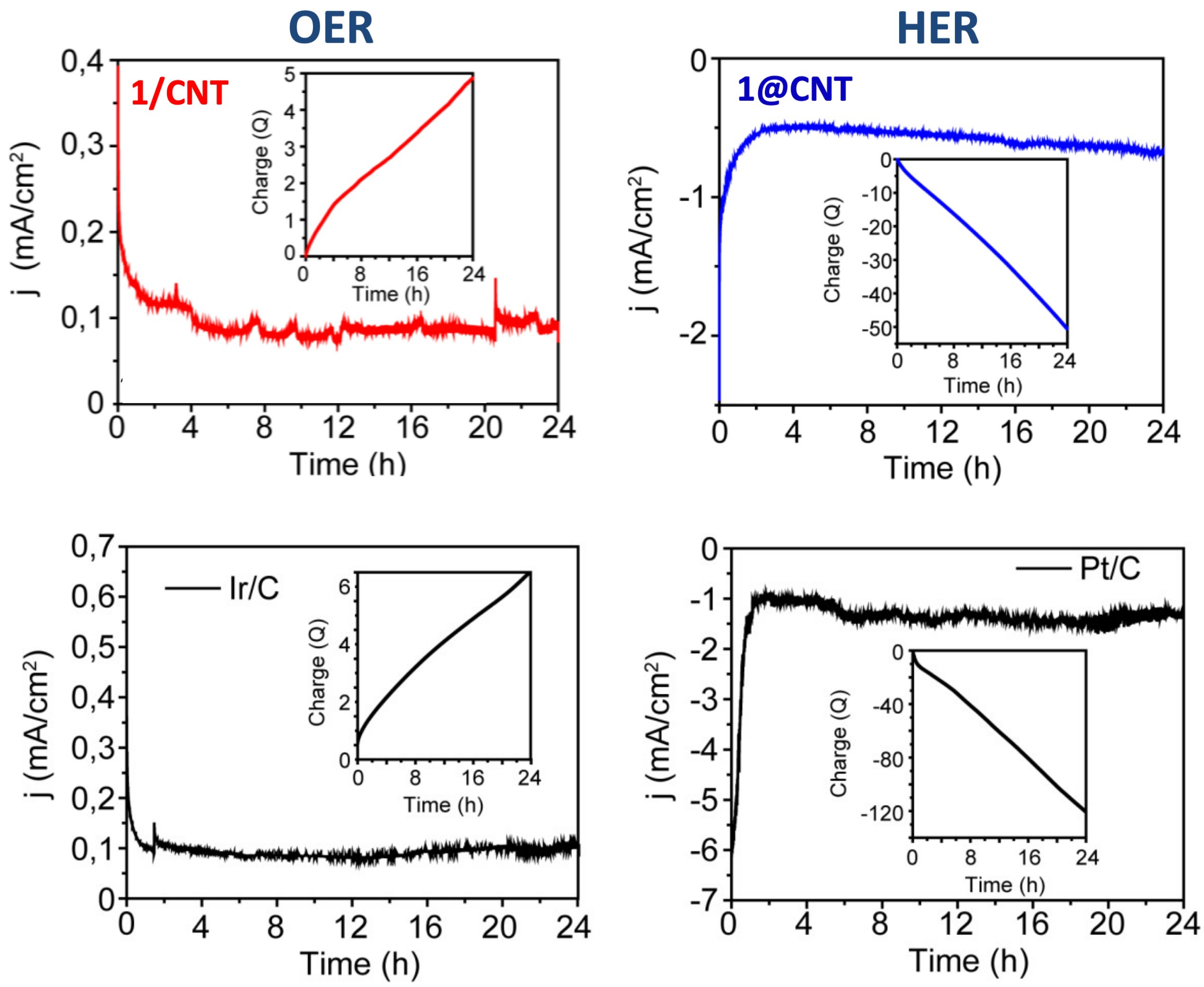

Figure S29. Comparison of the 24-hour chronoamperometry measurements for OER and HER. OER was performed using **1/CNT** at a constant potential of 1.48 V and compared to the Ir/C benchmark, while HER was carried out with **1@CNT** at –0.2 V and benchmarked against Pt/C.

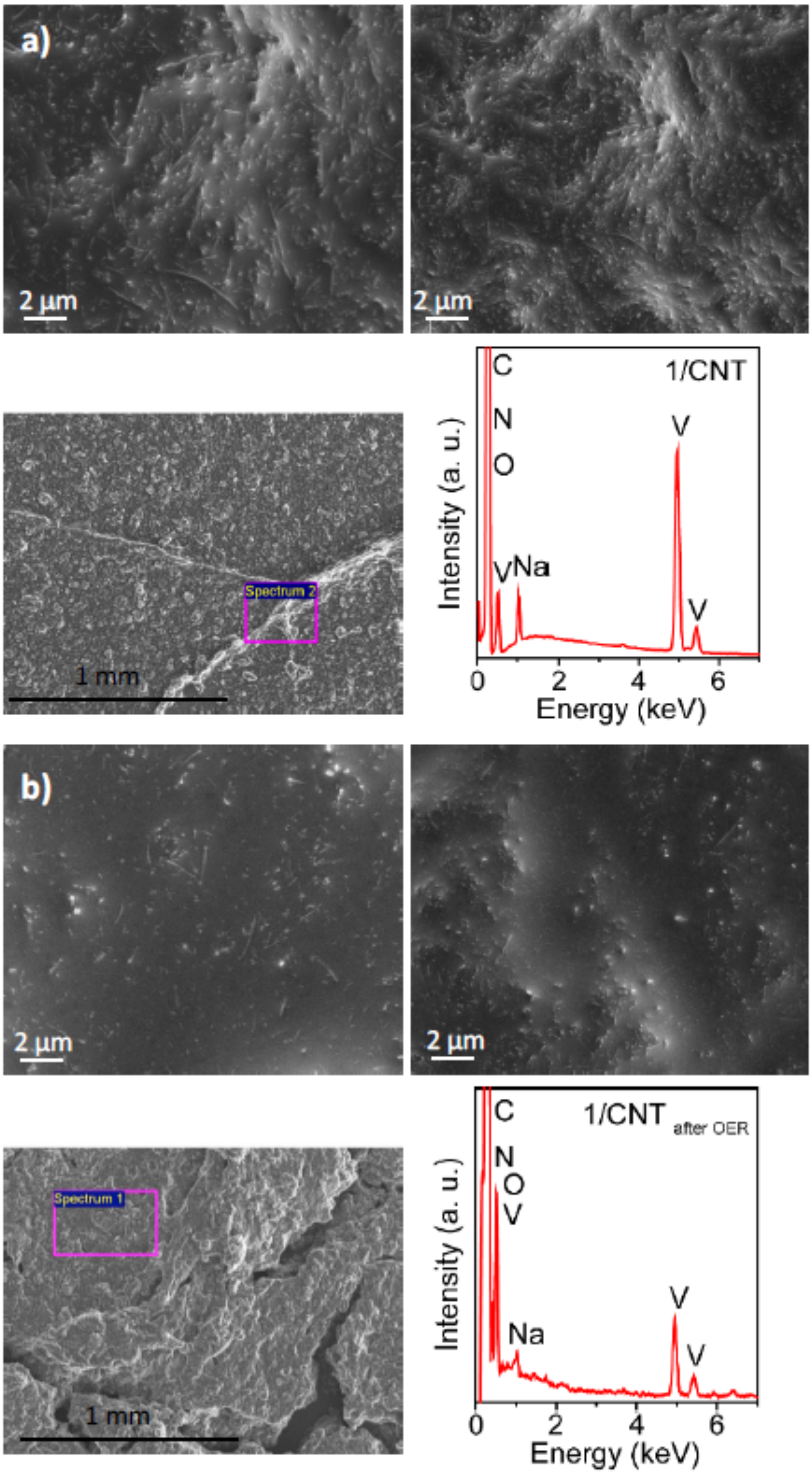

Figure S30. SEM images (20 kV) and EDX spectra of the **1/CNT** modified paste electrode before (a) and after (b) OER characterization, showing that leaching of **1** from the electrode to the aqueous electrolyte is prevented.

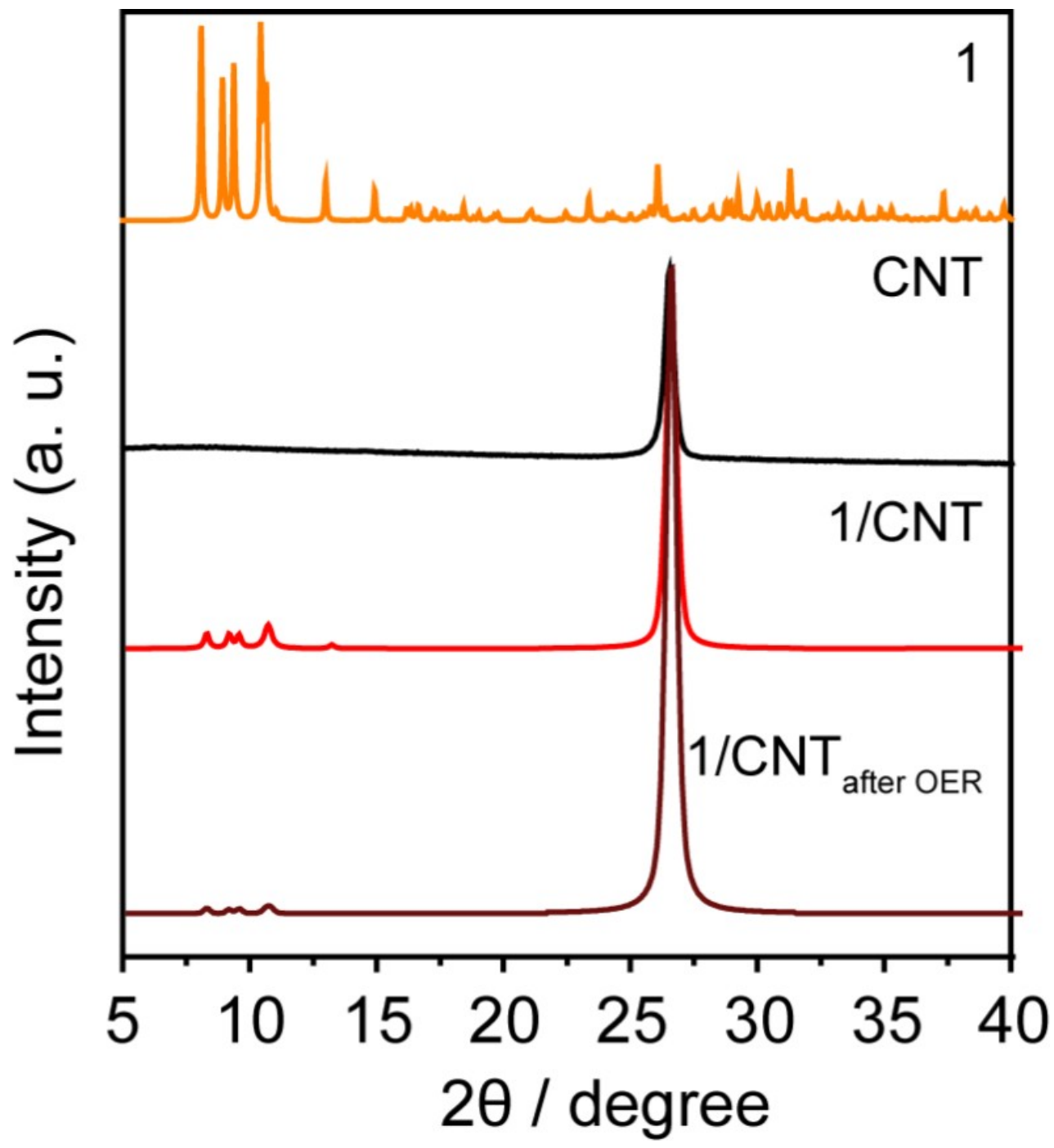

Figure S31. Powder XRD measurements of **1** (orange), **CNT** (black), **1/CNT** (red) and **1/CNT** after electrochemistry (brown) showing that 1 structure is maintain in **1/CNT** after harsh conditions: low pH (<0.3) and high potential (>1.2V).

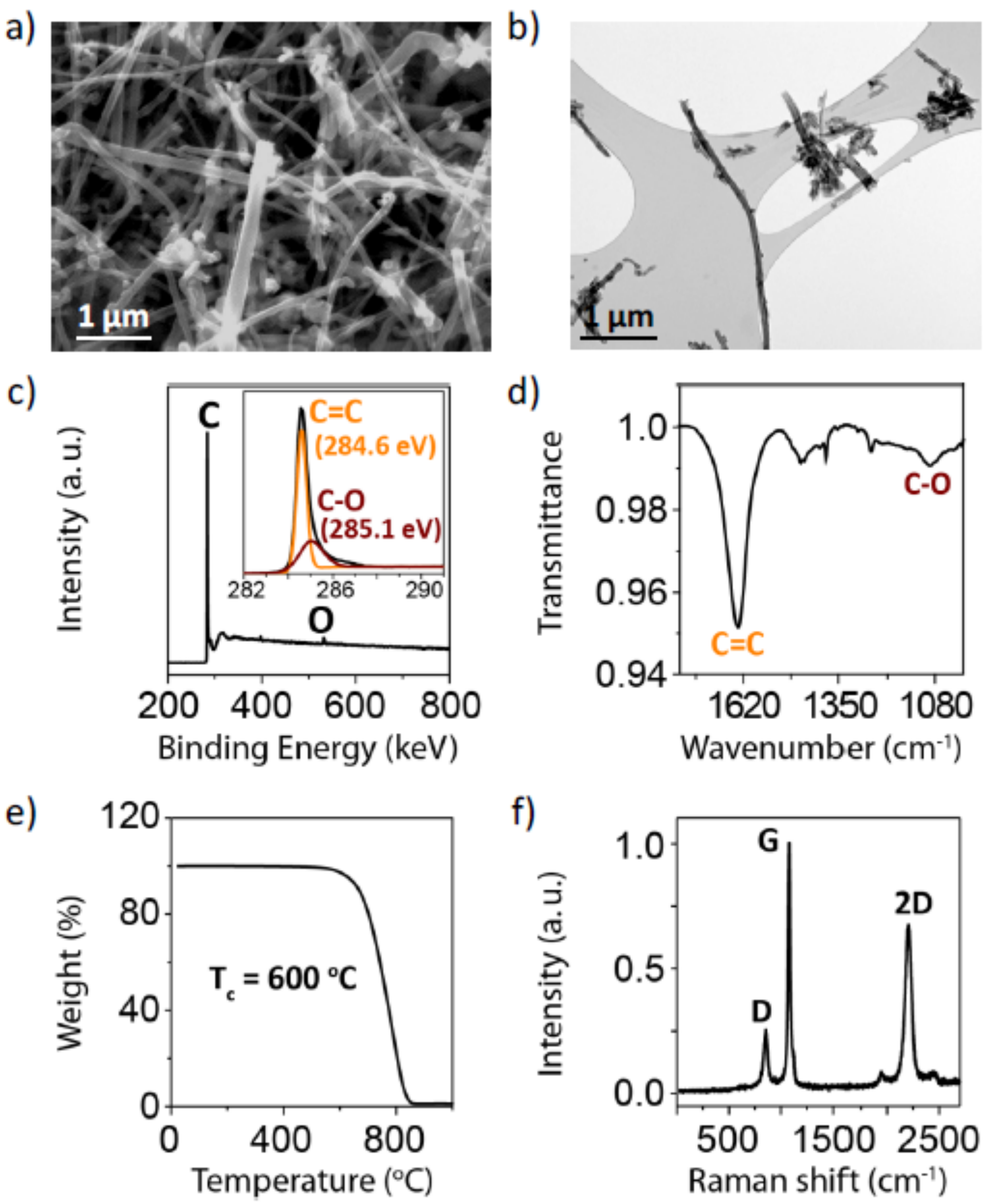

Figure S32. a) SEM and b) HR-TEM images at 20 kV and 200 kV, respectively, of CNT that have been shortened by ball milling. Both scale bars are 1µm. c) XPS wide-scan measurement of short CNT, showing O/C relation of 0.01, while C 1s high-resolution XPS measurements of CNT fitted with two components: C=C (284.6 eV) and C-C (285.1 eV) is shown in the inset. d) FTIR data for CNT assigning 1630 cm^{-1} (C=C), 1384 cm^{-1} (C-O bending), 1313 cm^{-1} (C-O stretching) and 1089 cm^{-1} (hydroxyl C-O stretching) peaks. e) TGA measurements of CNT at a scan rate of 5°C/min in air showing thermal decomposition of CNT above 600°C. f) Raman spectrum of CNT showing a D, G and 2D band at 1350, 1550 and 2700 cm^{-1}, having a I_D/I_G of 0.25.

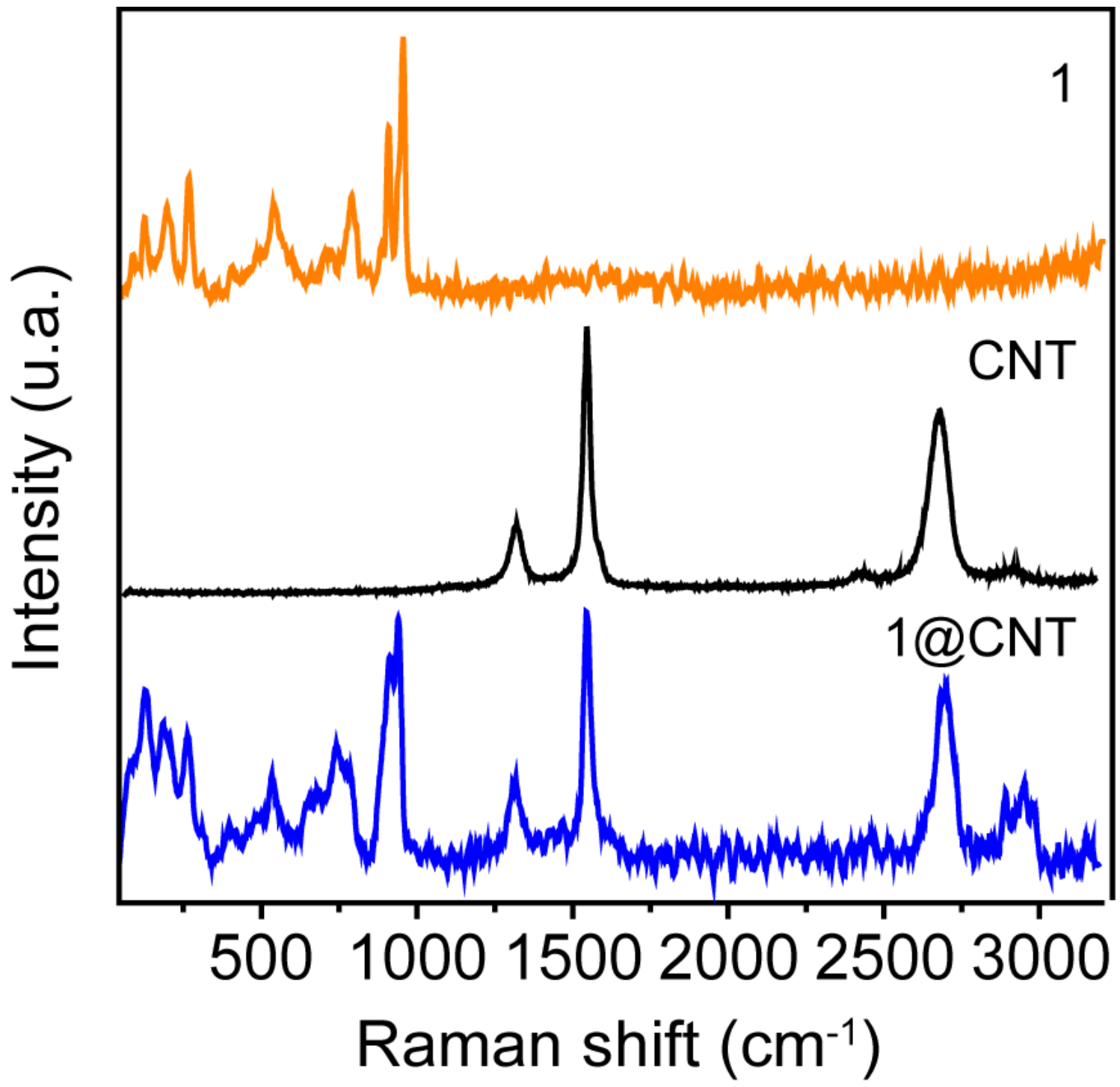

Figure S33. Raman measurements of **1** (orange), CNT (black) and **1@CNT** (blue) indicating that **1** structure is retained in **1@CNT**.

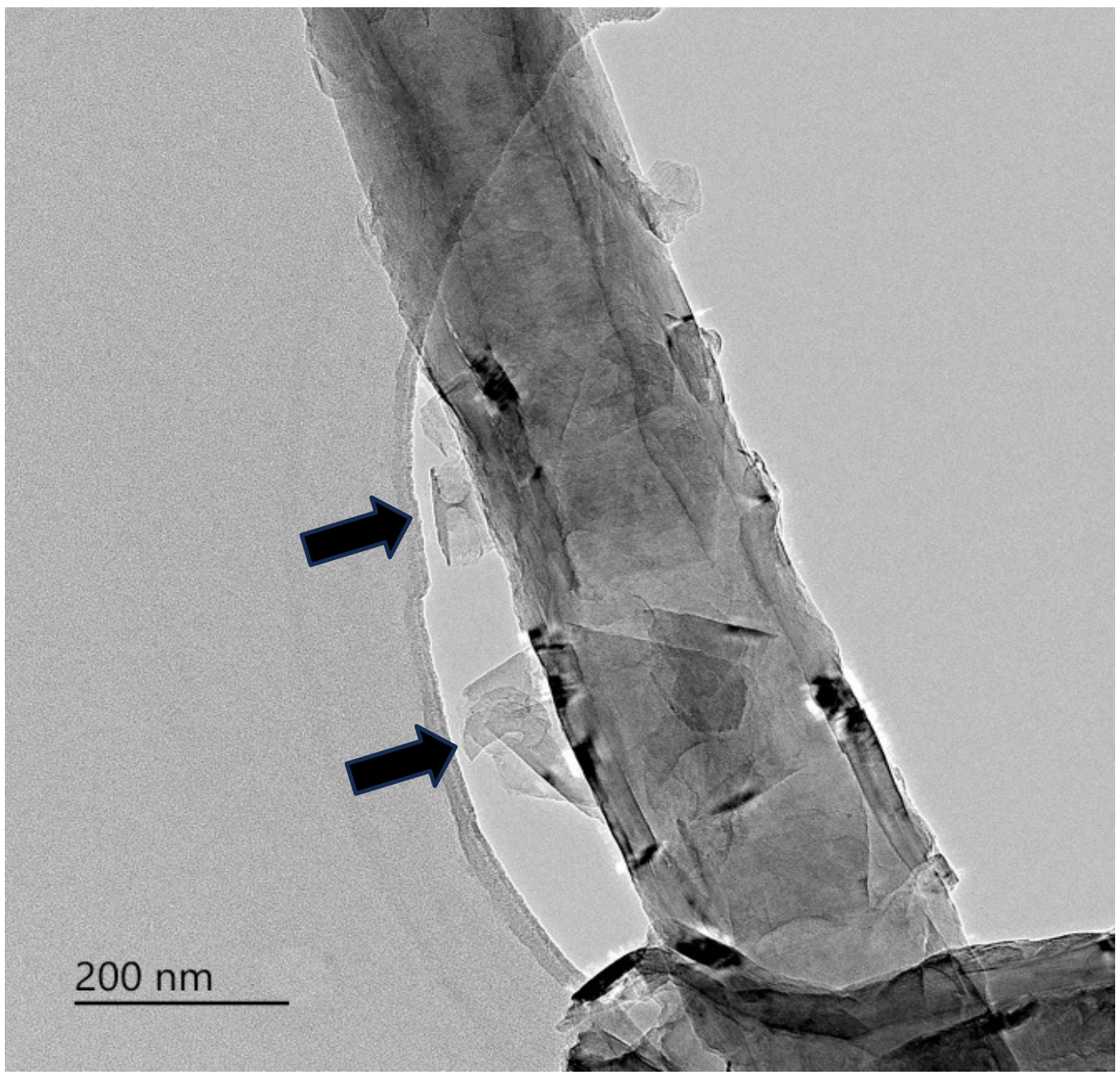

Figure S34. a) HR-TEM image at 200 kV of **1@CNT** supported on a copper grid.

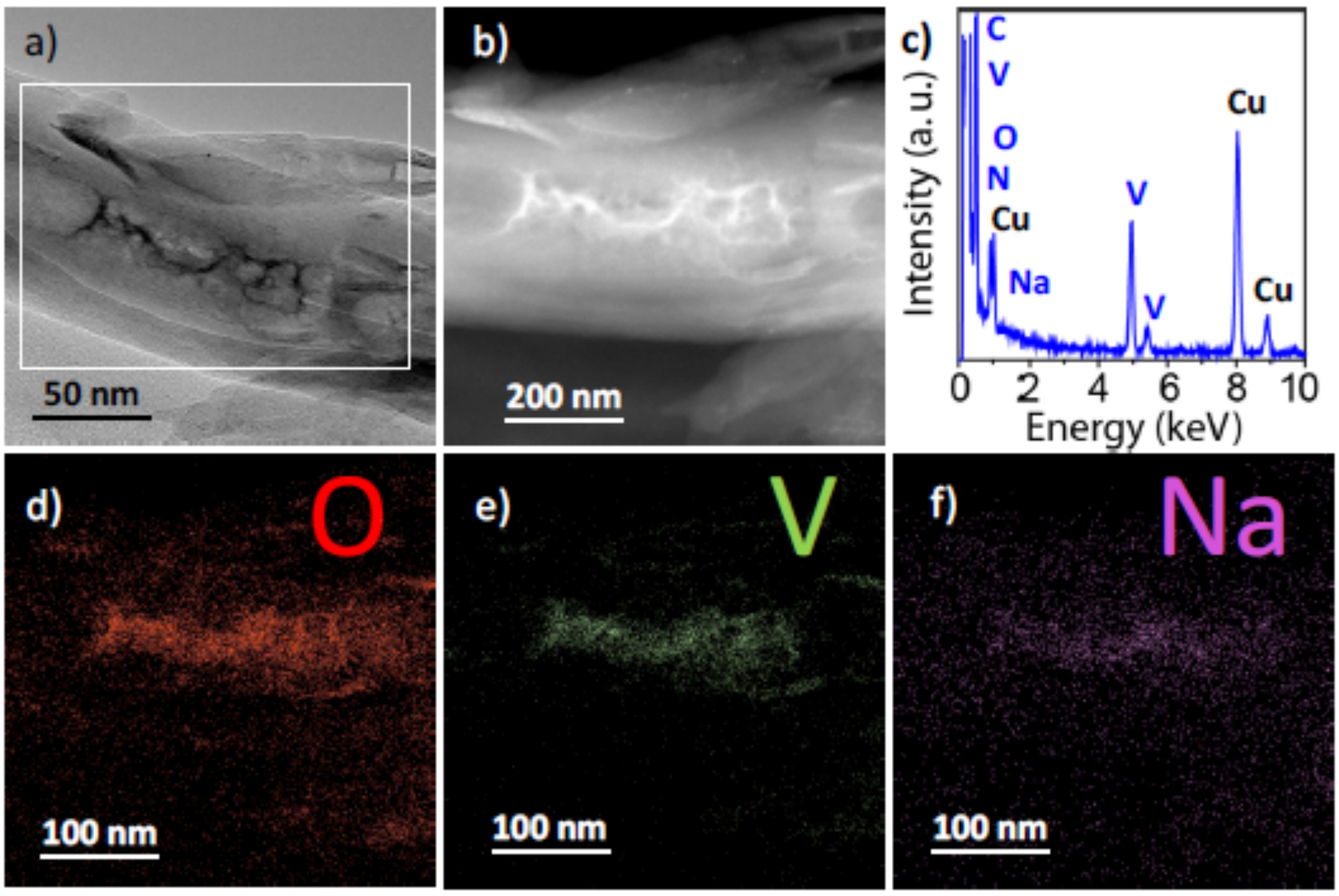

Figure S35. a) HR-TEM and b) dark-field STEM images at 200 kV, c) EDX spectrum of a selected area in (a); and STEM-EDS elemental mapping of (d) O, (e) V and (f) Na for **1@CNT** corresponding to the STEM-DF image in (b). Note that the presence of copper in (c) is due to the use of copper grids.

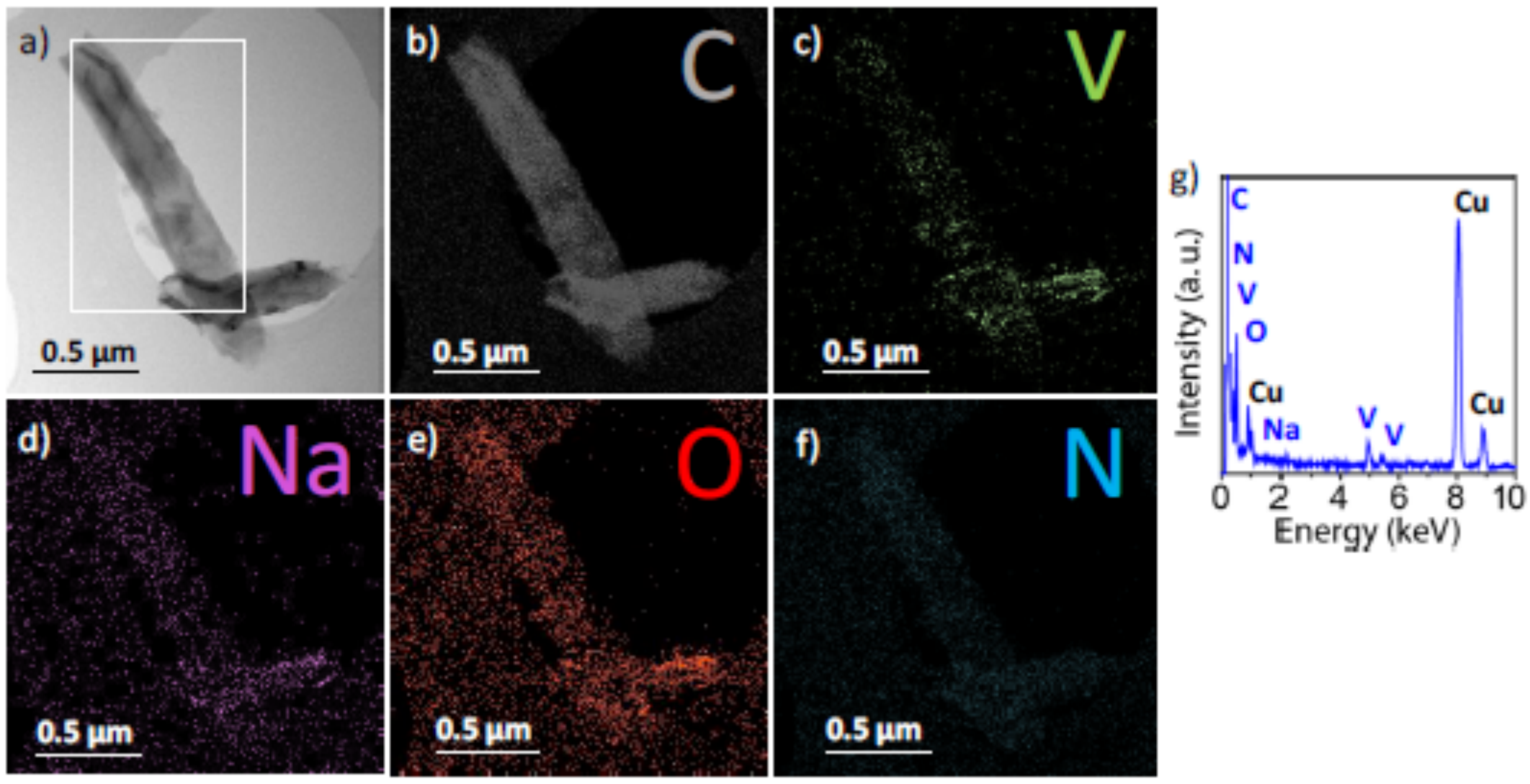

Figure S36. a) Bright-field STEM image at 200 kV, STEM-EDS elemental mapping of (b) C, (c) V, (d) Na, (e) O, (f) N and g) EDX spectrum of a selected area in (a) for **1@CNT**. Note that the presence of copper in (g) is due to the use of copper grids.

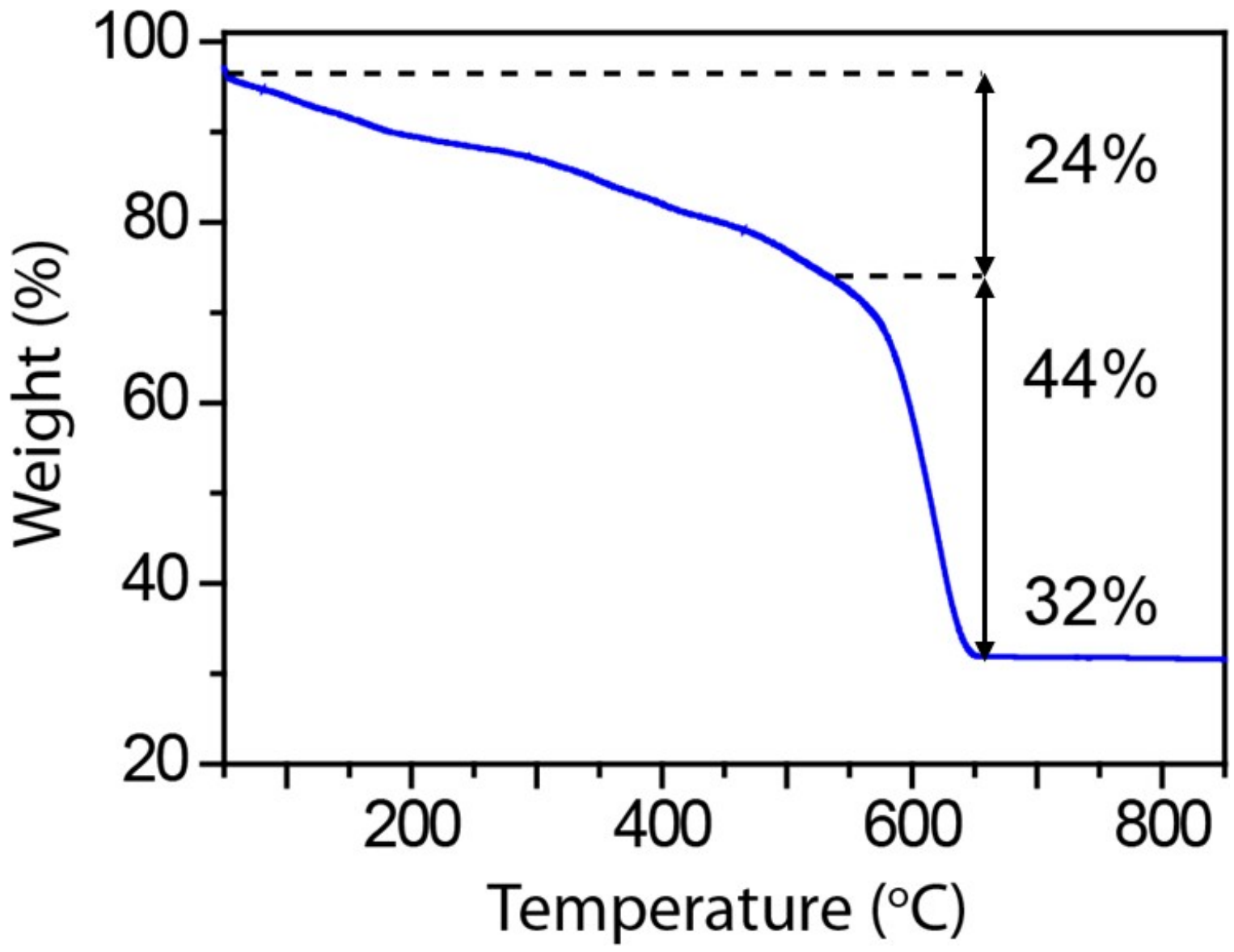

Figure S37. TGA measurements of **1@CNT** were performed in air up to 1000 °C with a heating rate of 5 °C/min. The weight loss observed below 400 °C can be attributed to strongly bound water molecules (associated with Na^{+} ions) and the organic moiety (TRIS). Above this temperature, the weight loss is associated with the decomposition of the $[V_{10}O_{28}]^{6-}$ clusters and CNT oxidation, yielding a residual weight of approximately 32%.

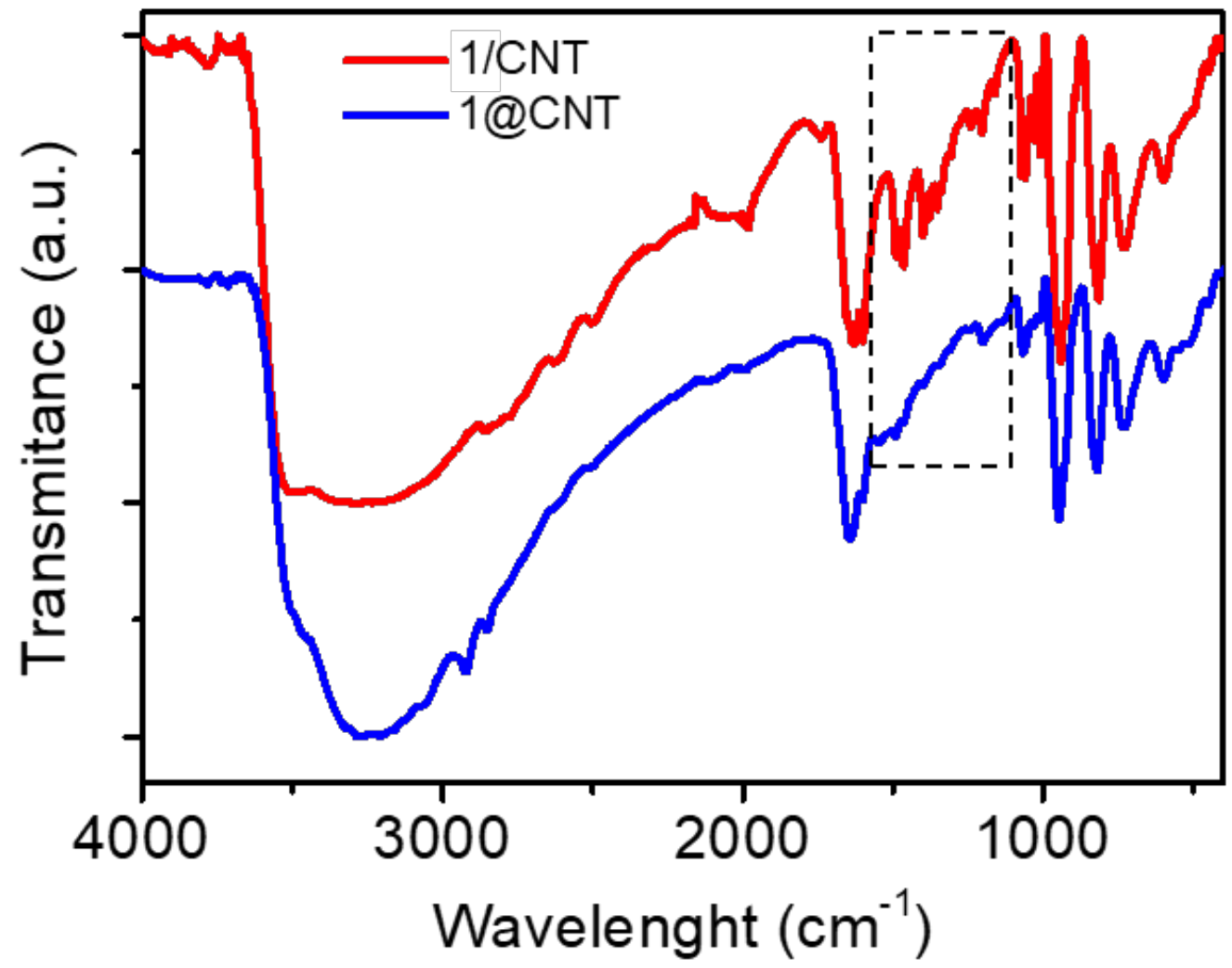

Figure S38. The selected region of the IR spectra shows peaks between 1500 and 1100 cm^{-1}, which correspond to stretching bands of ethers (such as Na–O–C and V–O–Na). These bands are observed only in **1/CNT** (physical mixture) but not in **1@CNT**, indicating the breaking of the Na–TRIS–Na chains.

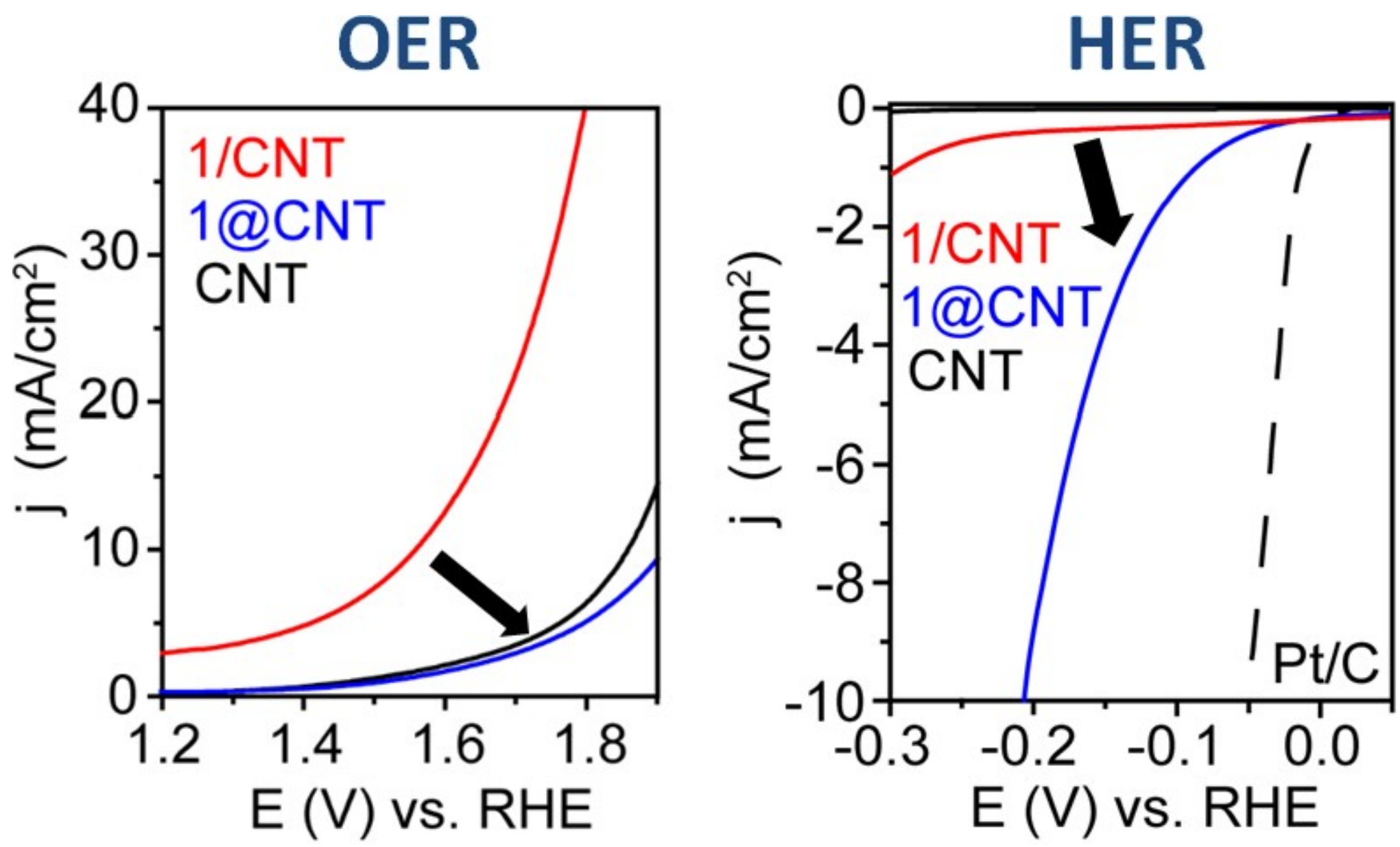

Figure S39. Comparison of the polarization curves of CNT (black), **1/CNT** (red), **1@CNT** (blue) for OER and HER. Pt/C was used as standard catalyst (dashed) for the HER. For all the electrochemical measurements, H_2SO_4 (1 M, previously degassed) was used as the electrolyte and the voltage potential is referred to the reversible hydrogen electrode (RHE).

Table S13. Comparative overview for the performance of different POM-based HER electrocatalyst materials.

**Material**	**Electrolyte**	**Onset Potential (mV)**	**Overpotential (mV) at -10 (mA/cm²)**	**Tafel Slope (mV/dec)**	**Reference**
1@CNT	1M H_2SO_4	-0.7	207	99	This work
h_MoN@BNCNT	0.5M H_2SO_4	-50	78	46	[15]
P_2W_{18}@rGF_ox	0.5M H_2SO_4	-	35	37	[16]
NENU-500	0.5M H_2SO_4	-100	237	96	[17]
$V_{10}O_{24}.nH_2O$	0.5M H_2SO_4	-0.1	118	101	[18]
Co_2P@Co_2P/Co-POM/NF	1M KOH	-	130	135	[19]

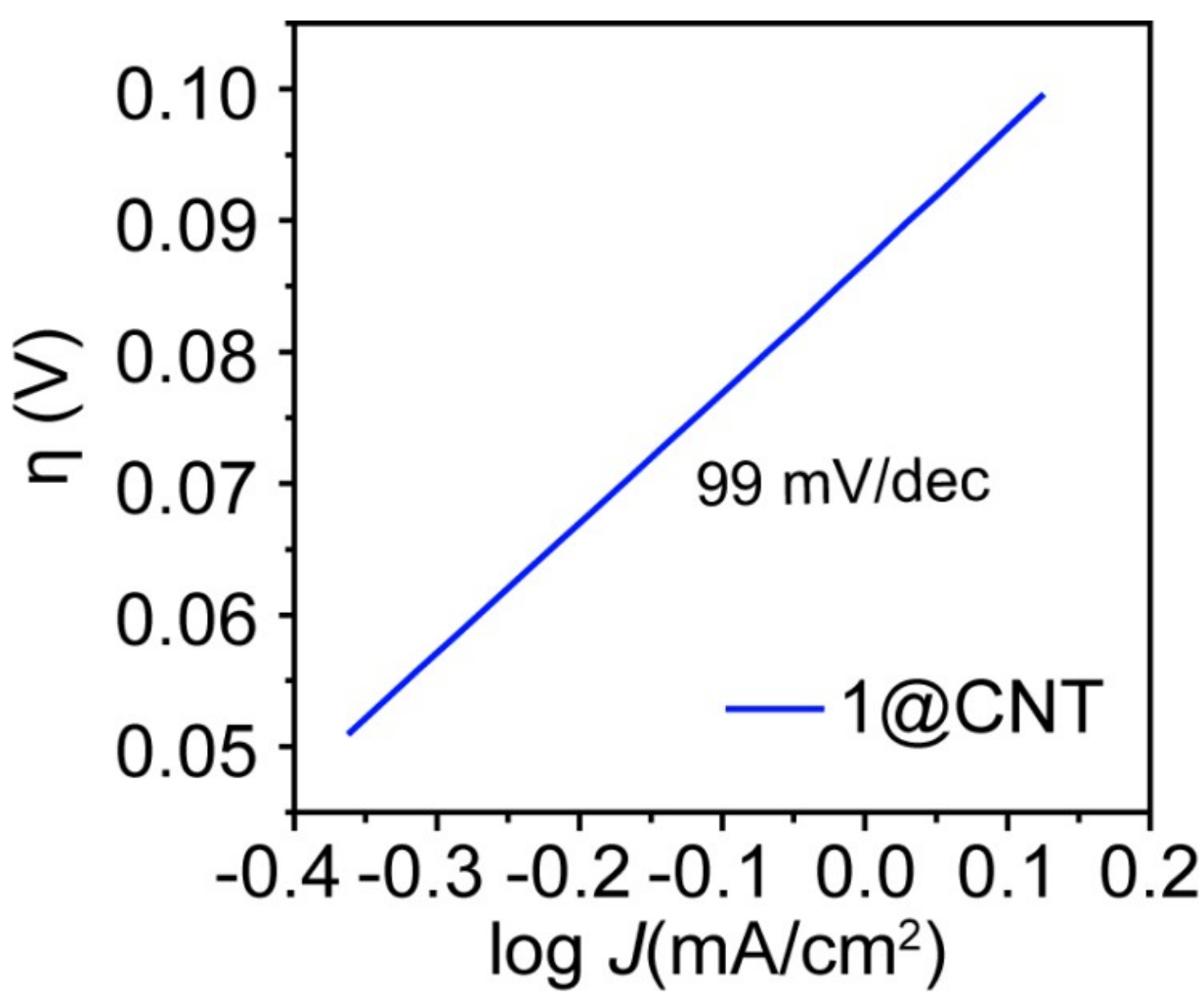

Figure S40. Tafel plot for the HER of **1@CNT**.

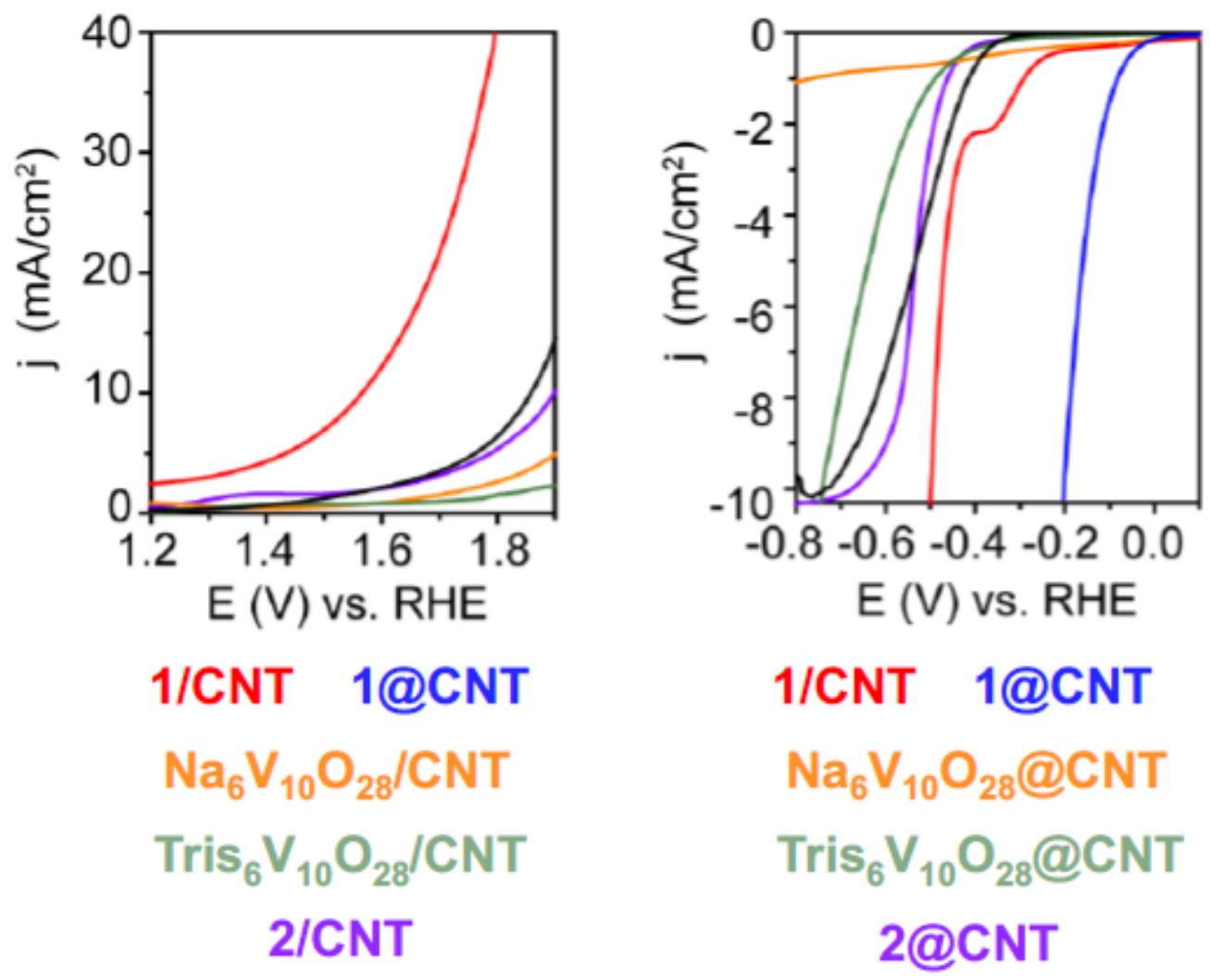

Figure S41. Electrocatalytic activity of the prepared vanadium-based POM in this work towards the OER (left) and HER (right).

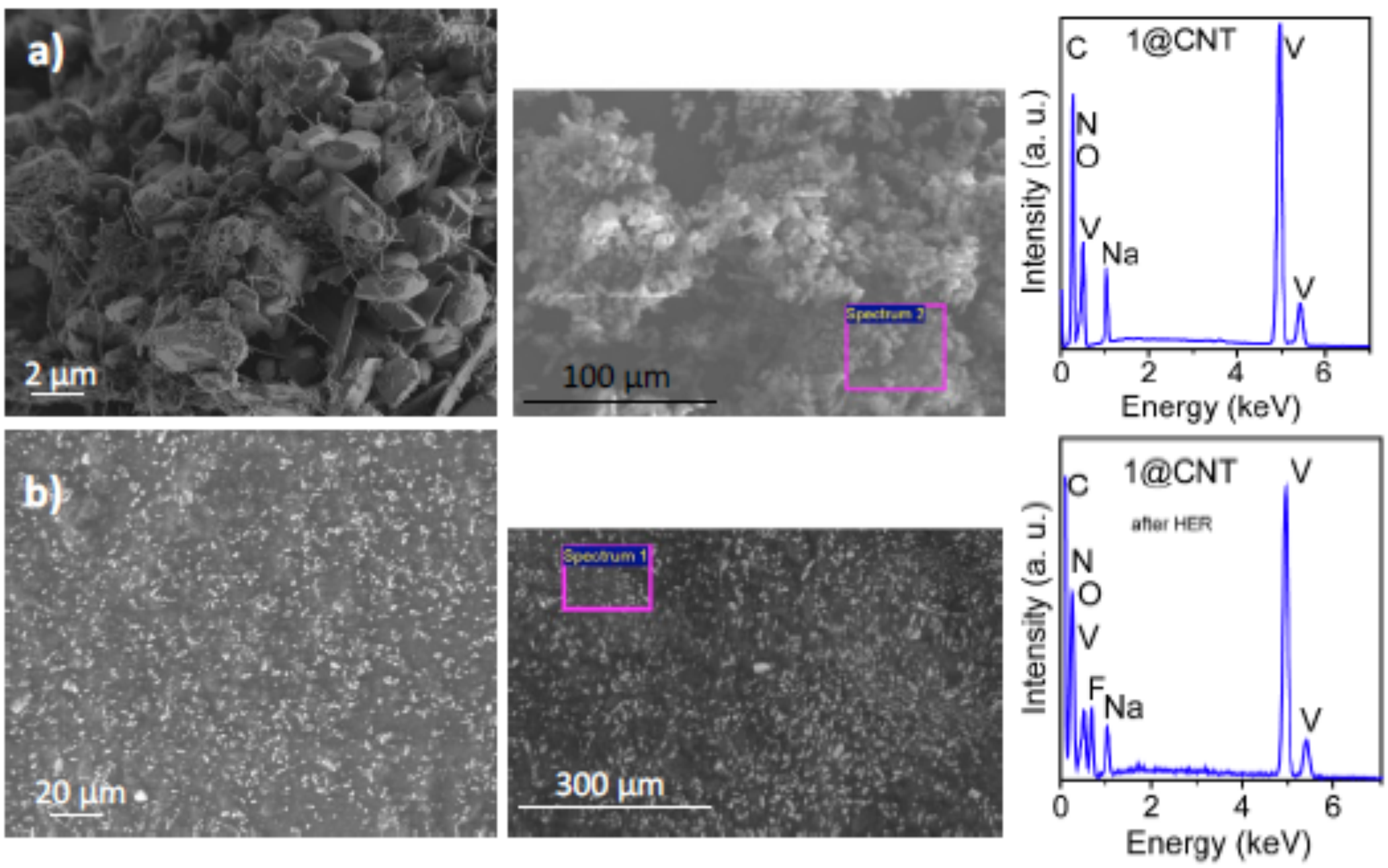

Figure S42. SEM images (20 kV) and EDX spectra of the **1@CNT** electrode before (a) and after (b) HER characterization, showing that leaching of **1** from the electrode into the aqueous electrolyte is prevented, and confirming the presence of V (8.86%), Na (2.55%), and N (6.17%).

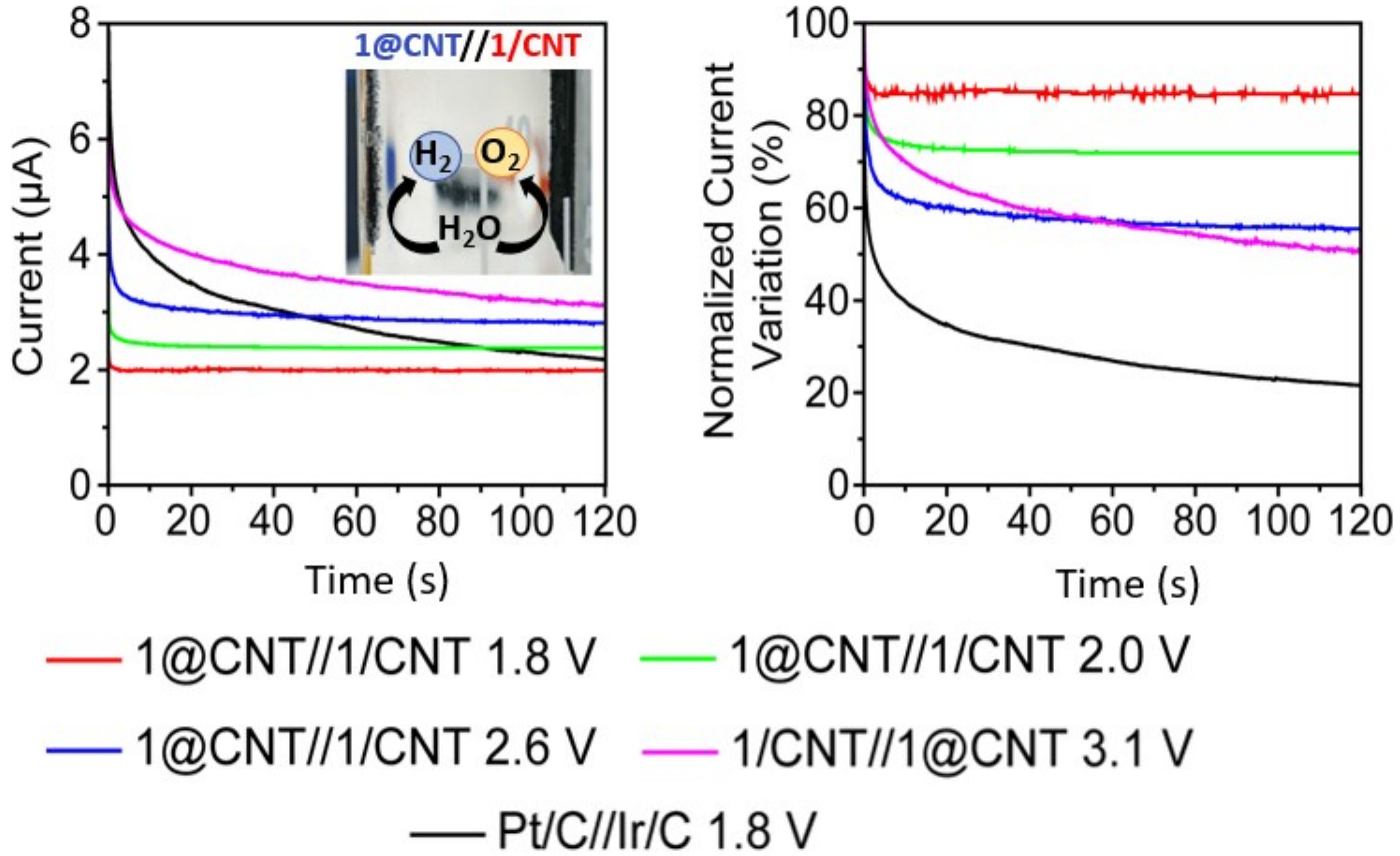

Figure 43. Comparison of the chronoamperometry measurements for **1@CNT//1/CNT** and Pt/C//Ir/C in a 2-electrode set-up under strong pH conditions (1M H_2SO_4 previously degassed).

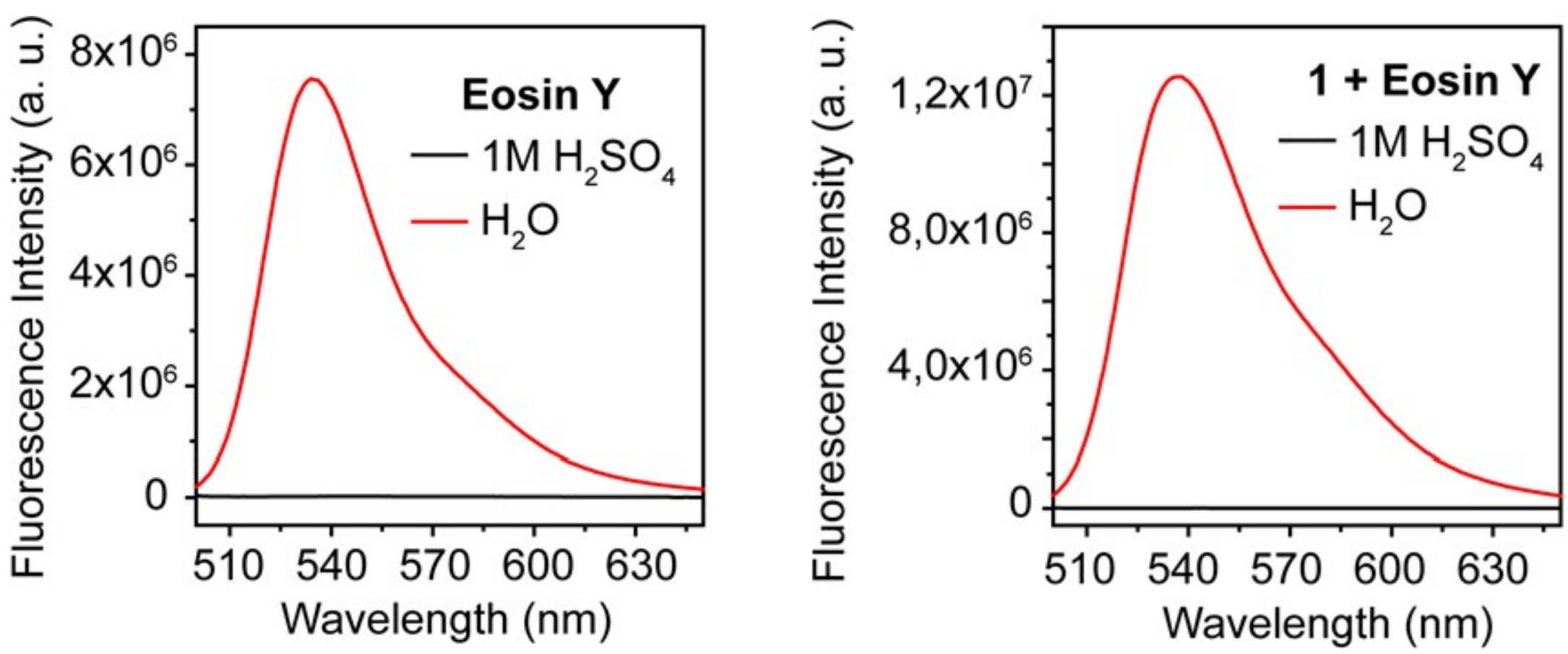

Figure S44. Fluorescence spectra of 10 µm eosin Y in in the pH 0 and 7 (1M H_2SO_4 and H_2O, respectively). Maximum emission at pH 7. Aqueous eosin YS changes from nonfluorescent at pH 0 to bright yellow fluorescent at pH 3, without any change in the excitation or emission spectra.[20] At the acidic medium where the electrochemical experiment in this study takes place, no fluorescence emission is shown.

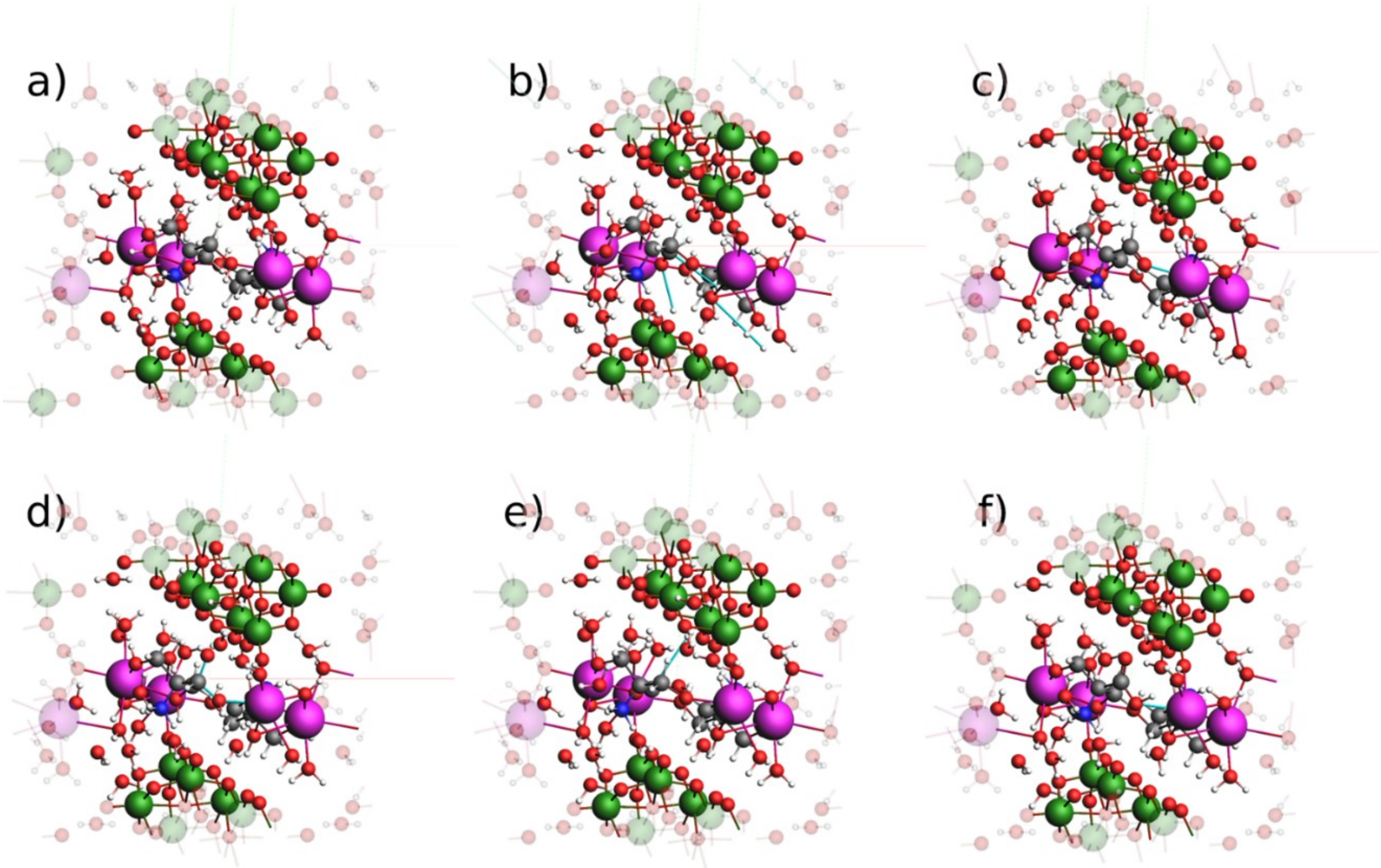

Figure S45. 2D periodic unit cell for the model calculations computed with the xtb-GFN1 Hamiltonian of a) Initial reference model, b) alcohol-aldehyde transformation triggered by the oxidation of a Na-bound water molecule with vanadate proton abstraction, c) aldehyde group interacting with a Na ion, d) diol group interacting with a Na ion, e) carboxylic formation through the α-H subtraction on a diol triggering oxygen oxidation with vanadate proton abstraction, f) carboxylic group near the Na ion. The turquoise bonds highlight transformations such as hydrogen transfer, direct interactions with Na atoms or water displacement. Oxygen atoms are rendered in red, carbon atoms in grey, and hydrogen atoms in white. Vanadium atoms and sodium atoms appeared as large spheres in green and pink respectively.

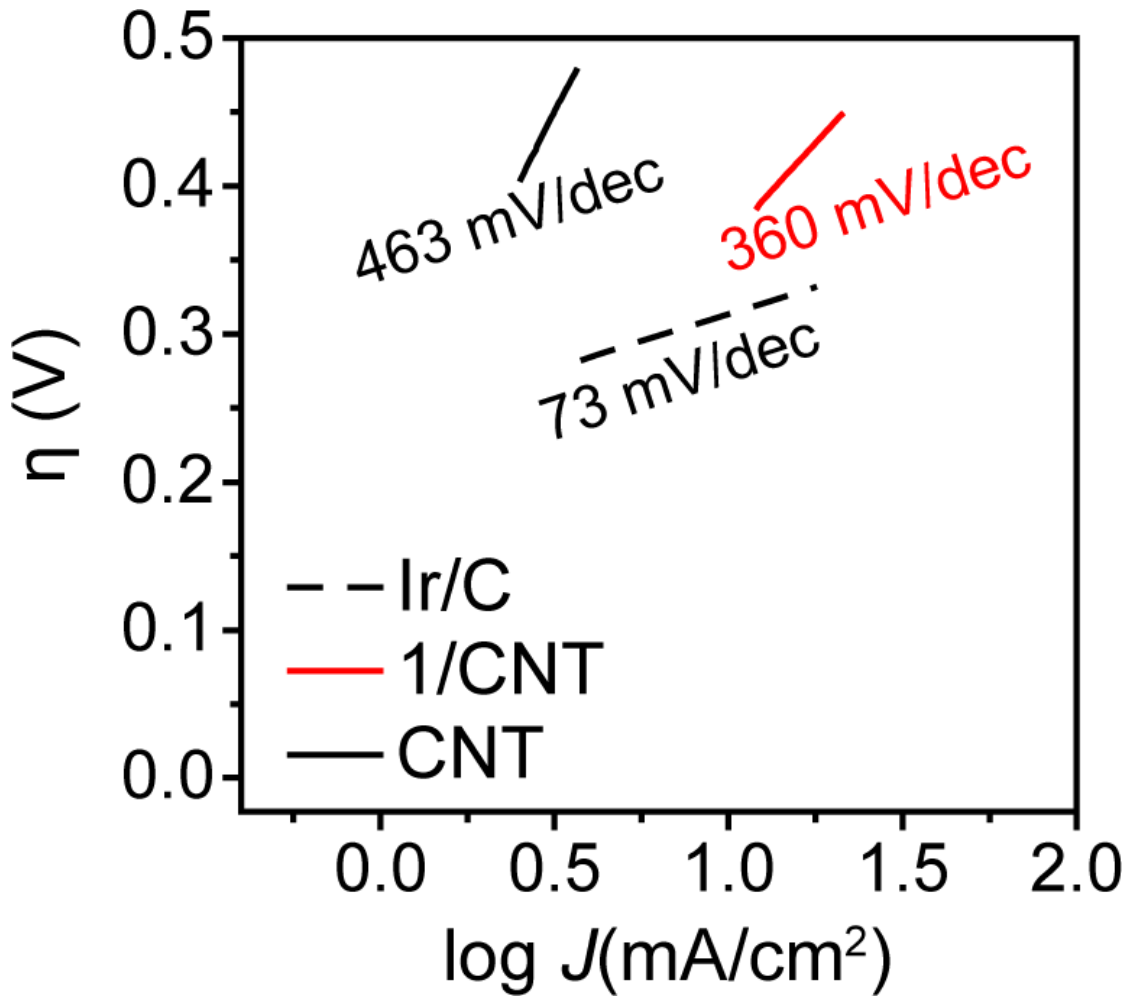

Figure S46. Tafel plot of OER of **Ir/C**, **1/CNT** and **CNT**

.

References

[1] J. M. Missina, L. B. P. Leme, K. Postal, F. S. Santana, D. L. Hughes, E. L. de Sá, R. R. Ribeiro, G. G. Nunes, Accessing decavanadate chemistry with tris (hydroxymethyl) aminomethane, and evaluation of methylene blue bleaching, *Polyhedron* **2020**, *180*, 114414.

[2] A. Durif, M. T. Averbuch-Pouchot, J. C. Guitel, Structure d'un décavanadate d'hexasodium hydraté, *Acta Crystallogr. B* **1980**, *36*, 680.

[3] A. P. Ginsberg, Ed., *Inorganic Syntheses: Volume 27*, McGraw Hill, New York, 1990.

[4] M. Aureliano, C. A. Ohlin, M. O. Vieira, M. P. M. Marques, W. H. Casey, L. A. E. B. de Carvalho, Characterization of decavanadate and decaniobate solutions by Raman spectroscopy, *Dalton Trans.* **2016**, *45*, 7391.

[5] J. Martín-Caballero, A. San José Wéry, S. Reinoso, B. Artetxe, L. San Felices, B. El Bakkali, G. Trautwein, J. Alcañiz-Monge, J. L. Vilas, J. M. Gutiérrez-Zorrilla, A Robust Open Framework Formed by Decavanadate Clusters and Copper(II) Complexes of Macrocyclic Polyamines: Permanent Microporosity and Catalytic Oxidation of Cycloalkanes, *Inorg. Chem.* **2016**, *55*, 4970.

[6] S. Yerra, B. K. Tripuramallu, S. K. Das, Decavanadate-based discrete compound and coordination polymer: Synthesis, crystal structures, spectroscopy and nano-materials, *Polyhedron* **2014**, *81*, 147.

[7] F. M. Zonoz, Electrochemistry investigation of the monolacunary and their transition metal substituent Keggin-type polyoxometalates, *Electrocatalysis* **2016**, *7*, 215.

[8] Y. Ding, H. Li, Y. Hou, Robust polyoxometalate-loaded nickel foam for electrocatalytic oxygen evolution reaction, *Mater. Lett.* **2018**, *221*, 264.

[9] Y. Wang, Y. Wang, L. Zhang, C.-S. Liu, H. Pang, PBA@ POM hybrids as efficient electrocatalysts for the oxygen evolution reaction, *Chem. – Asian J.* **2019**, *14*, 2790.

[10] M. Martin-Sabi, J. Soriano-López, R. S. Winter, J.-J. Chen, L. Vilà-Nadal, D.-L. Long, J. R. Galán-Mascarós, L. Cronin, Redox tuning the Weakley-type polyoxometalate archetype for the oxygen evolution reaction, *Nat. Catal.* **2018**, *1*, 208.

[11] J. J. Stracke, R. G. Finke, Distinguishing homogeneous from heterogeneous water oxidation catalysis when beginning with polyoxometalates, *ACS Catal.* **2014**, *4*, 79.

[12] M. Guillen-Soler, N. V. Vassilyeva, E. P. Quirós-Díez, J. M. Vila-Fungueiriño, A. Forment-Aliaga, M. del C. Gimenez-Lopez, A Hierarchical Polyoxometalate/Pd/Mos$_2$ Hybrid: Developing an Efficient Novel Bifunctional Catalyst for Water Splitting, *Adv. Sustain. Syst.* **2024**, *8*, 2300607.

[13] K. Azmani, M. Besora, J. Yu, A.-L. Teillout, P. de Oliveira, I.-M. Mbomekallé, J. Soriano-López, J. M. Poblet, J.-R. Galán-Mascarós, Water Oxidation Electrocatalysis in Acidic Media with Fe-Containing POMs/Carbon Composites, *Inorg. Chem.* **2025**, *64*, 4260.

[14] Y. Shimoyama, N. Ogiwara, Z. Weng, S. Uchida, Oxygen evolution reaction driven by charge transfer from a Cr complex to Co-containing polyoxometalate in a porous

ionic crystal, *J. Am. Chem. Soc.* **2022**, *144*, 2980.

[15] D. M. Fernandes, M. P. Araújo, A. Haider, A. S. Mougharbel, A. J. S. Fernandes, U. Kortz, C. Freire, Polyoxometalate-graphene Electrocatalysts for the Hydrogen Evolution Reaction, *ChemElectroChem* **2018**, *5*, 273.

[16] M. Jahan, Z. Liu, K. P. Loh, A Graphene oxide and copper-centered metal organic framework composite as a tri-functional catalyst for HER, OER, and ORR, *Adv. Funct. Mater.* **2013**, *23*, 5363.

[17] J.-S. Qin, D.-Y. Du, W. Guan, X.-J. Bo, Y.-F. Li, L.-P. Guo, Z.-M. Su, Y.-Y. Wang, Y.-Q. Lan, H.-C. Zhou, Ultrastable polymolybdate-based metal–organic frameworks as highly active electrocatalysts for hydrogen generation from water, *J. Am. Chem. Soc.* **2015**, *137*, 7169.

[18] K. K. Dey, S. Jha, A. Kumar, G. Gupta, A. K. Srivastava, P. P. Ingole, Layered vanadium oxide nanofibers as impressive electrocatalyst for hydrogen evolution reaction in acidic medium, *Electrochimica Acta* **2019**, *312*, 89.

[19] L. Zhang, X. Ding, M. Cong, Y. Wang, X. Zhang, *Int. J. Hydrog.* Self-adaptive amorphous Co2P@ Co2P/Co-polyoxometalate/nickel foam as an effective electrode for electrocatalytic water splitting in alkaline electrolyte, *Energy* **2019**, *44*, 9203.

[20] W. Khalid Hamood Al-Behadili, Y. M. Jawad, H. J. Hasan, Study the Effect of PH on Absorption and Fluorescence Spectra of Eosin Y Dye in Ethanol, *Asian J. Green Chem.* **2023**, *7*, 163.
